# High-risk multimorbidity patterns on the road to cardiovascular mortality

**DOI:** 10.1186/s12916-020-1508-1

**Published:** 2020-03-10

**Authors:** Nina Haug, Carola Deischinger, Michael Gyimesi, Alexandra Kautzky-Willer, Stefan Thurner, Peter Klimek

**Affiliations:** 1 Section for Science of Complex Systems, CeMSIIS, Medical University of Vienna, Spitalgasse 23, Vienna, A-1090 Austria; 2 Complexity Science Hub Vienna, Josefstädter Straße 39, Vienna, A-1080 Austria; 3 IIASA, Schloßplatz 1, Laxenburg, A-2361 Austria; 4 Santa Fe Institute, 1399 Hyde Park Road, Santa Fe, 85701 NM USA; 5 Gender Medicine Unit, Division of Endocrinology and Metabolism, Department of Internal Medicine III, Medical University of Vienna, Spitalgasse 23, Vienna, A-1090 Austria; 6 Gesundheit Österreich GmbH, Stubenring 6, Vienna, A-1010 Austria

**Keywords:** Disease trajectories, Comorbidities, Metabolic syndrome, Cardiovascular disease, Machine learning

## Abstract

**Background:**

Multimorbidity, the co-occurrence of two or more diseases in one patient, is a frequent phenomenon. Understanding how different diseases condition each other over the lifetime of a patient could significantly contribute to personalised prevention efforts. However, most of our current knowledge on the long-term development of the health of patients (their disease trajectories) is either confined to narrow time spans or specific (sets of) diseases. Here, we aim to identify decisive events that potentially determine the future disease progression of patients.

**Methods:**

Health states of patients are described by algorithmically identified multimorbidity patterns (groups of included or excluded diseases) in a population-wide analysis of 9,000,000 patient histories of hospital diagnoses observed over 17 years. Over time, patients might acquire new diagnoses that change their health state; they describe a disease trajectory. We measure the age- and sex-specific risks for patients that they will acquire certain sets of diseases in the future depending on their current health state.

**Results:**

In the present analysis, the population is described by a set of 132 different multimorbidity patterns. For elderly patients, we find 3 groups of multimorbidity patterns associated with low (yearly in-hospital mortality of 0.2–0.3%), medium (0.3–1%) and high in-hospital mortality (2–11%). We identify combinations of diseases that significantly increase the risk to reach the high-mortality health states in later life. For instance, in men (women) aged 50–59 diagnosed with diabetes and hypertension, the risk for moving into the high-mortality region within 1 year is increased by the factor of 1.96 ± 0.11 (2.60 ± 0.18) compared with all patients of the same age and sex, respectively, and by the factor of 2.09 ± 0.12 (3.04 ± 0.18) if additionally diagnosed with metabolic disorders.

**Conclusions:**

Our approach can be used both to forecast future disease burdens, as well as to identify the critical events in the careers of patients which strongly determine their disease progression, therefore constituting targets for efficient prevention measures. We show that the risk for cardiovascular diseases increases significantly more in females than in males when diagnosed with diabetes, hypertension and metabolic disorders.

## Background

Noncommunicable (or chronic) diseases (NCDs) such as dorsalgia, hypertension, respiratory diseases or diabetes affect a large fraction of the world’s population and decrease the quality of life of people burdened by them [1]. For example, in 2004, almost 50% of the population of the USA was suffering from at least one chronic condition [2], and in 2014, approximately 25% of the adults had been diagnosed with more than one NCD [3], with both numbers strongly increasing with age. Besides having a negative impact on people’s well-being, NCDs also pose a substantial burden on the healthcare systems of countries [4, 5]. Apart from combatting common risk factors such as unhealthy lifestyle and diet, which many NCDs are attributed to [6], early detection and management play a key role in decreasing the burden of chronic diseases [7]. Personalised (or stratified) medicine promises to make care more efficient by tailoring treatments individually to patients while taking their individual risk profile for potential future diseases into account [8]. A necessary precondition to enter into this new era of medicine is a deepened understanding of how the long-term developments of multiple diseases condition each other.

The field of network medicine holds as central tenet that diseases cannot be studied independently from each other but arise from complex interactions between molecular units in the human body in terms of discretised structures such as protein–protein, metabolic, regulatory and RNA networks [9]. For example, the fact that many diseases tend to be comorbid, i.e. that they tend to co-occur in patients, can be understood based on the disease-causing genes or pathways shared by the comorbid diseases [10–12]. However, not all diseases linked by shared genes exhibit significant comorbidities, as different mutations of the same gene can have different consequences. Conversely, not all comorbidities can be explained from molecular data. One reason is that comorbidities can also be the result of the exposure to the same environmental risk factors, for example, due to a particular lifestyle [13]. In addition, present data on molecular interactions is far from being complete [14].

To obtain a more empirically based understanding of disease comorbidity, observational healthcare data regarding hospital admissions, pharmaceutical prescriptions and doctor visits has been leveraged for the purpose of disease prediction [15, 16]. Using so-called phenotypic comorbidity networks [17–20]—networks where nodes represent diseases and two nodes are linked if the two corresponding diseases are comorbid—it was shown that diseases which are comorbid to many other diseases have a higher mortality and that patients tend to develop diseases in close network proximity to the ones they already have. The latter fact opens up the possibility to predict future diseases of patients based on their medical history. This has been accomplished with approaches that include collaborative filtering [21, 22], frequent itemsets [23, 24], learning of transition probabilities between states represented by binary vectors [25, 26], deep learning [27, 28] and point processes [29].

Next to predicting future disease, longitudinal data on hospital admissions allows the identification of previously unknown patterns in the temporal sequence of diseases (disease trajectories) patients are diagnosed with. By analysing the temporal order of diagnosis pairs co-occuring in a significant number of patients with a particular directionality in time, it was possible to identify such trajectories with a length of up to four diseases [30]. Alternative methodological approaches to identify typical disease trajectories include dynamic time warping [31], a method originally developed for speech recognition, or the detection of putative causal relations between pairs of diseases using information-theoretic approaches [32]. Recently, non-negative matrix factorisation was used to extract multimorbidity patterns out of a large data set of electronic health records, and these were then used to describe long-term disease trajectories of patients [33]. The major challenge all these works are confronted with is the high number of different diagnosis codes, leading to a combinatorial explosion of the number of possible disease combinations, many of which occur only in a single patient.

**Fig. 1:**
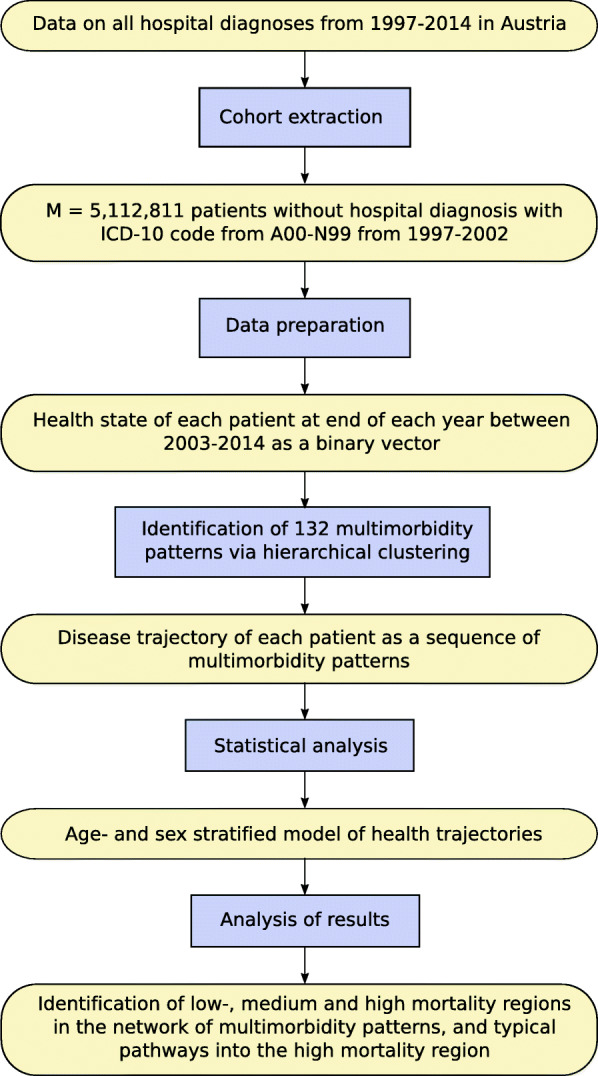
Workflow of the research presented in this article. Rounded boxes represent the input or results, and rectangles represent the steps of the analysis

Here, we present an alternative approach to characterise the population of an entire country in terms of its long-term disease trajectories. An illustration of our work flow is shown in Fig. 1. Based on a longitudinal data set covering all intramural stays in Austria from 1997 to 2014, see the “Methods” section, we extract the cohort of all patients who have not been assigned a diagnosis with ICD-10 code from A00–N99 between 1997 and 2002. This ensures that all patients have the same initial health state (absence of diagnoses over 6 years). We then represent the health state of each patient at the end of each year from 2003 to 2014 by a binary vector, a list of zeros and ones that encode which diseases a patient has (or has not) been diagnosed so far. The set of all health states (disease vectors) is then partitioned into a set of multimorbidity patterns, called clusters, by using a hierarchical clustering algorithm called DIVCLUS-T [34]. Each cluster is defined by a set of diseases which each patient in that cluster has been diagnosed with (inclusion criteria) and a set of diseases each patient in that cluster has *not* been diagnosed with (exclusion criteria). As time proceeds, patients acquire new diseases and consequently can change the cluster they belong to, with probabilities depending on their age and sex. The sequence of clusters a patient belongs to throughout the years describes his or her disease trajectory. We construct a multiplex network where nodes represent clusters and layers correspond to different patient sex and age groups. Directed links in the different layers are weighted according to the rate at which patients of the corresponding sex and age group change between the clusters corresponding to the connected nodes.

Our approach provides a statistical model for the disease progression of patients which takes into account their sex, age and entire observed history of hospital diagnoses. In particular, it can be used to explore differences between men and women in the risk for developing certain sets of diseases given the same set of pre-existing conditions. We characterise the different clusters in terms of their age and sex composition, as well as the in-hospital mortality of patients assigned to them. Elderly patients are often found in one of three network regions with different in-hospital mortalities. We find two regions with low and medium mortality, where patients have been diagnosed with eye diseases and arthropathies, respectively. However, most patients are found in a set of strongly connected clusters in the network characterised by hypertension (ICD-10 I10–I15), heart diseases (I20–I52) and high in-hospital mortality. We identify clusters which are visited by many patients prior to entering into this high-mortality cardiovascular disease region. We see them as targets for aggressive risk management in order to prevent or delay the onset of cardiovascular disease. The fact that the high-risk clusters are characterised by known risk factors for cardiovascular disease like obesity, metabolic disorders and diabetes, and the fact that their effect is found to be stronger in women compared with men, serves as a validation of our approach. Beyond these more or less obvious results, our approach identifies dozens of other transitions with significant risk differences between men and women (disregarding sex-specific diseases).

## Methods

### Data

We analyse a data set provided by the Austrian Federal Ministry for Health, covering all approx. 45,000,000 hospital stays of about 9,000,000 individuals in Austria during the 17 years from 1997 to 2014. Here, the term hospital also includes facilities of long-term care such as rehabilitation centres or psychiatric hospitals [35]. For each hospital stay, the data includes the sex (male/female) and age group (5-year interval) of the patient, the main and side diagnoses associated with the stay in the form of level-3 ICD-10 codes [36] and the admission and release date and the release type (e.g. normal release, transfer or death); 54% of all death cases in Austria between 1997 and 2014 are recorded in the data set, i.e. happened during a stationary hospital stay. Depending on the year, the maximum number of diagnoses assigned during 1 hospital stay fluctuates between 30 and 40, the mean number of diagnoses assigned during 1 stay fluctuates between 2.6 and 2.7, and the median number is always 3. We restrict our analysis to 1074 codes from A00 to N99, grouped into 131 blocks as defined by the WHO, see Additional file 1: Table S1. Our study population consists of the number of individuals *M*=5,112,811 patients not assigned a diagnosis from the range A00 to N99 during a hospital stay in Austria in the period from 1997 to 2002 inclusively. Note, however, that all patients appearing in the data set were assigned at least 1 diagnosis code between A00 and Z99 between 1997 and 2014.

### Clustering

The (observed) health state of a patient at a given point in time consists of the set of all diseases they have been diagnosed with until that point. We measure the health state of each patient at the end of each year in the observation period 2003–2014. Using a divisive clustering algorithm called DIVCLUS-T [34], we partition the set of all observed health states into *K*=132 clusters, see Additional file 1: Section S1. The obtained clusters represent typical multimorbidity patterns which can be used to describe the health states of patients on a coarse-grained level. Each cluster is characterised by a set of diseases each patient in that cluster has been diagnosed with (inclusion criteria) and a set of diseases each patient in that cluster has *not* been diagnosed with (exclusion criteria). We characterise the clusters according to the sex distribution and mean age, as well as (in-hospital) mortality, defined as the percentage of patients in that cluster who die in-hospital per year.

### Analysis of cluster transitions

The disease trajectory of a patient can now be represented by a temporal sequence of multimorbidity patterns which describe the health state of this patient at different points in time. We use the terms health state and disease history interchangeably. If their health state is assigned to cluster *j* in a given year, and to cluster *k* in the next year, then we say that the patient has stepped from cluster *j* to cluster *k*. By construction, for each pair of clusters, there is at least one disease which is an inclusion criterion in one and an exclusion criterion in the other cluster. As we represent the health states of patients by all diseases which they have been diagnosed with so far, a patient cannot step from a cluster in which a certain disease is an inclusion criterion to one where this disease is an exclusion criterion. Therefore, steps between two clusters are only possible in one direction.

The cohort is stratified by sex and 10-year age groups. For a given sex *g* and age group *a*, we measure the probability that a patient is found in cluster *j*, given that he or she was in cluster *k* 1 year earlier: the cluster transition rate *q*_*g,a,k,j*_.

From all patients in a given set of clusters, we define progredient patients as those that are found outside of this set of clusters after 3 years. Differences in comorbidities between progredient and non-progredient patients are evaluated by means of Fisher’s exact test; for multiple testing, we adjust using the Bonferroni correction.

The cluster transition rates specify a probabilistic model for the disease progression of patients, see Additional file 1: Section S4. The model can be represented by a multilayer network, a set of nodes with multiple directed links between them. In the network, nodes correspond to the disease clusters (health states) and links between clusters to the transition rates. Each age and sex group specifies one network layer with link weights given by the rates *q*_*g,a,k,j*_.

## Results

### Disease clusters

Inclusion and exclusion criteria of all 132 obtained clusters are listed in Additional file 1: Tables S2–S132. There and in the main text, the symbols ✗ and ✓ next to a diagnosis block indicate that this block is an exclusion and an inclusion criterion, respectively, for that cluster. Initially, all patients are free of any (known) prior diagnoses, and their trajectories start in cluster 0 (the “healthy cluster”), where all diagnoses are exclusion criteria. In Additional file 1: Figure S3, we show the cluster sizes, meaning the total number of health states (disease vectors), assigned to each cluster, colour-coded according to the sex distribution within the clusters. There are several clusters only containing patients of 1 sex; this is explained by sex-specific inclusion criteria such as diseases of male genital organs (N40–N51). Other clusters with strong sex imbalance include cluster 70, where 82% of the patients are male. For this cluster, the inclusion criteria are mental and behavioural disorders due to psychoactive substance use (F10–F19) and diseases of the liver (K70–K77).

**Fig. 2:**
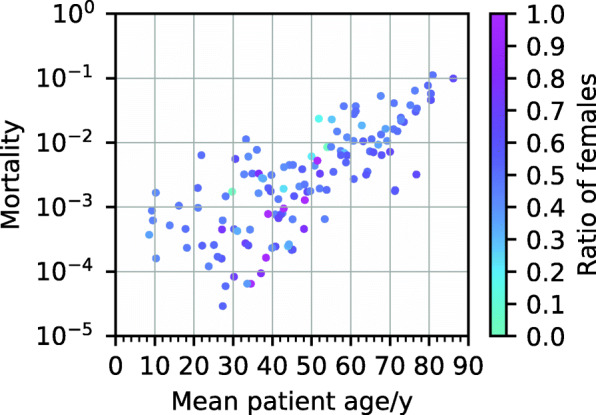
Scatter plot of mortality versus mean age in the different clusters with colours indicating the sex distribution in the corresponding cluster

**Fig. 3:**
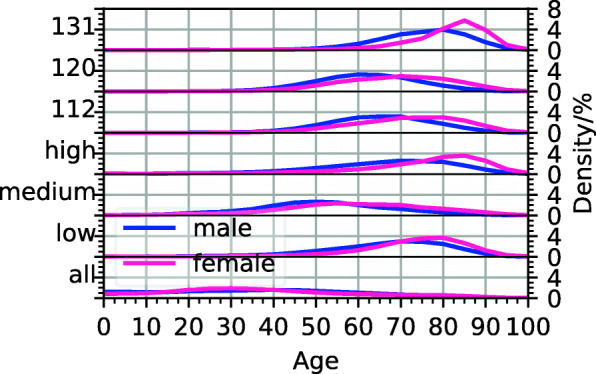
Age distributions of males and females in all clusters and in the low-, medium- and high-mortality regions (below), and in the high-risk clusters 112 (diabetes and hypertension) and 120 (diabetes, hypertension and metabolic disorders) and the high-mortality sink state, cluster 131 (hypertension, heart diseases, diseases of the arteries, arterioles and capillaries and renal failure) (above)

Clusters differ strongly in their age composition. For most clusters, the age distribution is centred around ages 60–80, see Additional file 1: Figure S1. In Fig. 2, we compare the mean age with the mortality across clusters. Mortality tends to increase with mean age; however, the mortalities in 2 clusters with the same average age can differ by more than a factor of 100. With 11%, cluster 131 has the highest mortality. This cluster has no exclusion criteria and includes hypertensive diseases (I10–I15); heart diseases (I30–I52); diseases of the arteries, arterioles and capillaries (I70–I79); and a diagnosis of renal failure (N17–N19). Moreover, 67% of the health states assigned to that cluster include metabolic disorders (E70–E90), and 49% include diabetes mellitus (E10–E14). The age profile of cluster 131 is shown in Fig. 3; women in that cluster tend to be older than men.

Most clusters associated with high mortality have hypertensive diseases (I10–I15) and heart diseases (I30–I52) as inclusion criteria. Clusters 9, 47 and 48, which also show high mortality, contain patients who have received diagnoses of heart diseases, but none of hypertension. Other clusters with high mortality are cluster 11, including cerebrovascular diseases (I60–I69) but excluding hypertension, and clusters 55, 56, 74 and 109, which include malignant neoplasms (C00–C97). Although we cannot rule out the possibility that some patients in cluster 11 were not diagnosed with hypertension despite suffering from it, we note that this cluster has a significantly different age profile than cluster 107, where both cerebrovascular diseases (I60–I69) and hypertension (I10–I15) are inclusion criteria. More precisely, 18% of the patients in cluster 11 are aged less than 40, compared to only 2% in cluster 107. Moreover, 17% of the patients in cluster 11 have been diagnosed with subarachnoid haemorrhage (I80), compared to 7% in cluster 107.

### Disease trajectories

**Fig. 4:**
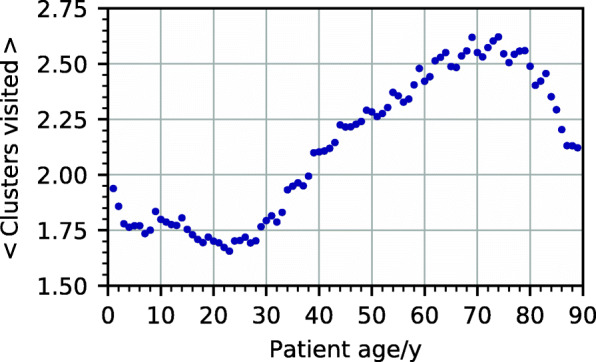
Average number of clusters visited by patients during the observation period depending on their age at the beginning of the observation period

**Fig. 5:**
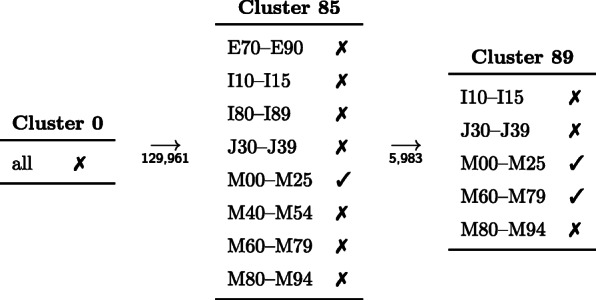
The reduced trajectory (0, 85, 89), which is the most frequent reduced trajectory of length 3 and followed by 5983 patients of the cohort. The numbers underneath the arrows give the total number of patients who step from cluster 0 to cluster 85 and from cluster 0 to cluster 85 to cluster 89, respectively

We define the *reduced* disease trajectory of a patient as the disease trajectory with all repetitions removed; the length of a reduced trajectory is the number of different health states it contains. For example, if patient *i* has the disease trajectory (0,1,1,1,5,5,3), then the reduced disease trajectory is (0,1,5,3), which has length 4. Additional file 1: Figure S7 shows the distribution of the average length of the reduced disease trajectory of patients during the observation period. Almost 25% of the patients stay in cluster 0 during the entire time span (they only received codes from the ICD chapter O–Z); 2% visit 4 or more clusters. Figure 4 shows how the length of the reduced disease trajectory of patients varies with age. Adolescent patients visit the least number of clusters, and patients around the age of 70 visit the highest number of clusters. Two reasons might contribute to this decline. First, mortality strongly increases after the age of 80, meaning that these elderly patients have less time to collect high numbers of hospital visits. Second, there might be selection bias at work. Patients of our cohort were not assigned hospital diagnoses from A00 to N99 during 1997–2003. Elderly patients fulfilling these criteria are, therefore, less likely to suffer from chronic diseases, leading to less hospital visits. The most frequent reduced trajectory of length 3 is (0, 85, 89) and is followed by 5983 individuals. Patients with this reduced trajectory first acquire a diagnosis of arthropathies (M00–M25) and subsequently 1 of soft tissue disorders (M60–M79), see Fig. 5.

We now describe the results for typical disease trajectories involving diagnoses of cerebrovascular diseases, malignant neoplasms and mood disorders.

#### Cerebrovascular diseases (I60–I69)

In total, 199,681 patients (3.9%) were diagnosed with cerebrovascular diseases. The distribution of the length of their reduced trajectories is shifted towards higher values compared to the total cohort, with more than 50% of the patients following reduced trajectories of length greater than 2, see Additional file 1: Figure S7. The most frequent reduced trajectory of length 3, followed by 1447 patients, is (0, 114, 123), meaning that patients from the “healthy cluster” 0 (all diseases are exclusion criteria) move to cluster 114 with hypertensive diseases (I10–I15) and heart diseases (I30–I52) to cluster 123 where cerebrovascular diseases (I60–I69) changed from an exclusion (cluster 114) to an inclusion criterion (cluster 123).

#### Malignant neoplasms (C00–C97)

A total of 312,787 patients (6.1%) received diagnoses of malignant neoplasms. More than 50% of them only visit 2 different clusters, and 44% (69,552) of those patients have the reduced trajectory (0, 55), i.e. from the healthy cluster to a cluster where cancer (C00–C97) is the only inclusion criterion. The most frequent reduced trajectory of length 3, followed by 3093 cancer patients, is (0, 55, 109) where patients acquire hypertensive diseases (I10–I15) after cancer.

#### Mood (affective) disorders (F30–F39)

A total of 210,589 patients (4.1%) of the study cohort are diagnosed with mood or affective disorders during the observation period. Among them, 9.5% end their trajectory in cluster 71 where, additional to mood disorders, they are diagnosed with mental and behavioural disorders due to psychoactive substance use (F10–F19). The most common reduced trajectory of length 3 in this subcohort, followed by 1936 patients, is (0, 64, 71). These patients first acquire a diagnosis from the block F10–F19 and subsequently 1 from F30–F39.

### Disease cluster multiplex network

**Fig. 6:**
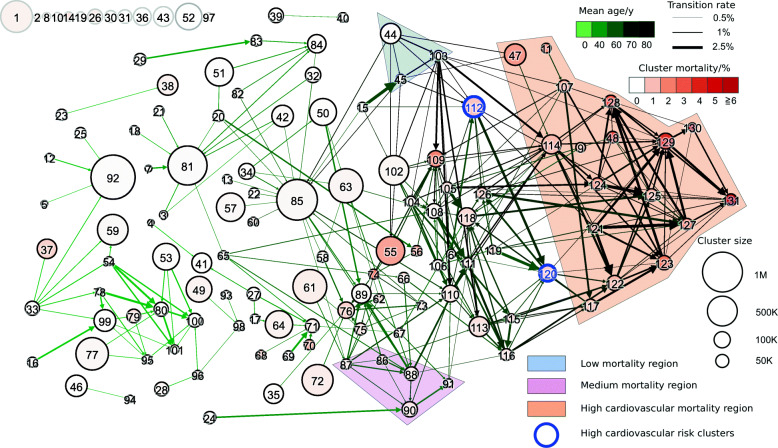
Visualisation of the multilayer network. Nodes represent clusters, and directed edges between the nodes are weighted according to the rate at which patients move between them. The direction of the edges is always from the node with lower to the one with higher label; in most cases, this is from left to right. The edges are coloured depending on the average age of the transitioning patients: light (dark) green edges indicate their low (high) average age. Node size is proportional to the size of the corresponding cluster. The node colour indicates the cluster mortality, with white standing for low and red standing for high mortality. For clarity, in the visualisation, we removed cluster 0 (the “healthy cluster”) which is connected to all other nodes. The layout has been generated by a force-directed algorithm after which nodes have been displaced from left to right by a distance proportional to the average age associated with its links, i.e. patient age tends to increase from left to right. To improve readability, we only display a filtered version of the network with the links representing statistically robust transition rates greater than 0.5*%*. Nodes which are isolated under this threshold have been aligned in the upper left corner. For elderly patients, we observe network regions of low, medium and high mortality. Patients access the high-mortality regions via the high-risk clusters 112 and 120 (blue circles)

Figure 6 shows a visualisation of the obtained multilayer network of patient health states. Nodes correspond to clusters, and their size is the number of observations in that cluster; the node colour corresponds to annual cluster mortality on a scale from 0% (white) to 6% and above (red). For this picture, all network layers have been collapsed into one by omitting stratification according to sex and age. Thickness of the links is therefore proportional to the rate at which patients change between the clusters they connect, irrespective of sex and age. To increase clarity, we only show the links which correspond to the transition rates of more than 0.5% and which are traversed by a sufficiently high number of patients to make the calculated rates robust against statistical fluctuations, see Additional file 1: Section S3 for our notion of robustness. The network layout has been chosen such that the average age of patients in the clusters increases from left to right. Clusters are labelled such that the direction of the links always goes from the cluster of lower to the one with higher label which typically means younger to older age, or from left to right. Self-loops are omitted.

**Fig. 7:**
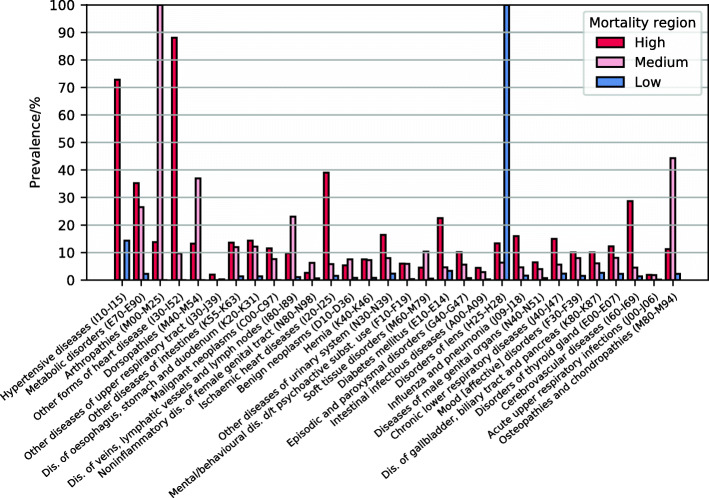
Comparison of the prevalences of the 30 most frequent diseases in the high-, medium- and low-mortality regions for the elderly

There exist several subsets of clusters which are highly interconnected, meaning that patients move frequently between them, see Additional file 1: Figure S2. Clusters 102–131 share hypertensive diseases (I10–I15) as a common inclusion criterion, and most patients assigned to them are aged older than 60 years, see Additional file 1: Figure S1. Clusters 114, 121–125 and 127–131 additionally include heart diseases (I30–I52) and are characterised by high mortality (2–11%). Together with clusters 9, 11, 47, 48 and 107, which also have high patient mortality attributable to cardiovascular diseases, we therefore refer to these clusters as the high-cardiovascular mortality region of the multilayer network, highlighted orange in Fig. 6. In particular, cluster 131, which has the highest mortality of all clusters, is part of the (cardiovascular) high-mortality region. This cluster has no outgoing links in the cluster multilayer network and therefore can be regarded as a sink state of our model, meaning that patients entering this cluster do not leave it any more. Note that the fact that such a sink state exists is a consequence of our choice of clustering algorithm and the representation of patients by their disease histories. Another densely connected block consists of clusters 86–91 and is characterised by diagnoses of arthropathies (M00–M25). With the exception of clusters 85 and 89, they all have an average age greater than 55 years and mortalities of 0.3–1 *%*. We refer to this block as the medium-mortality region for the elderly, highlighted pink in Fig. 6. Clusters 44, 45 and 103 share disorders of lens (H25–H28) as a common inclusion criterion. Patients included in them have mean age greater than 70 years and mortalities of 0.2–0.3%. We call these clusters the low-mortality region for the elderly, highlighted blue in Fig. 6. Age profiles of the 3 discussed regions are shown in Fig. 3, and in Fig. 7, we show the prevalences of the 30 most frequent diagnosis blocks in them.

### High cardiovascular risk clusters

We identify those clusters via which most patients enter the high-cardiovascular mortality region. To this end, we rank all other clusters according to the number of patients entering the high-mortality region via them. Most patients step into the high-mortality region directly from the “healthy” cluster 0, meaning that (during the observation period) they did not receive hospital diagnoses before, see Additional file 1: Figure S4. The four other clusters ranked highest are clusters 102, 112, 118 and 120. Apart from cluster 118, which has ischaemic heart diseases (I20–I25) as an inclusion criterion, these clusters do not include cardiovascular diseases and are characterised by patient mortalities of less than 1.5%. As inclusion criteria, they have combinations of diabetes mellitus (E10–E14), metabolic disorders (E70–E90) and hypertensive diseases (I10–I15). The rates at which patients step into the high-mortality region from clusters 112 to 120 are significantly increased compared with all other patients of the same age and sex. Moreover, we find this increase to be stronger in females than in males. The risk of stepping into the high-mortality region within 1 year from cluster 112 (diabetes and hypertension) is increased by a factor of 1.96 ± 0.11 (2.60 ± 0.18), 1.57 ± 0.06 (1.71 ± 0.10) and 1.38 ± 0.05 (1.49 ± 0.05) for men (women) aged 50–59, 60–69 and 70–79, respectively, compared to all patients of the same sex and age group. For cluster 120 (diabetes, metabolic disorders and hypertension), the corresponding values are 2.09 ± 0.12 (3.04 ± 0.18), 1.58 ± 0.08 (2.17 ± 0.10) and 1.47 ± 0.07 (1.59 ± 0.07). Here, the numbers to the right of the symbol “ ±” quantify the extent of the 95% confidence interval, see Additional file 1: Section S3. Among the progredient male patients aged 50–59 in the high-risk clusters, 16 ± 4% have been diagnosed with chronic obstructive pulmonary disease (J44), compared with 8.3 ± 0.6% of those who remain stable in the high-risk clusters without proceeding into the high-mortality region. Of the progredient female patients aged 60–69, 23 ± 3% have received a diagnosis of obesity (E66) compared with 14.3 ± 0.8% of the stable ones. For men of the same age group, the corresponding numbers are 11 ± 2% and 10.1 ± 0.8%, respectively. Note that although the increase in cardiovascular risk for patients in the high-risk clusters is stronger in females than in males, the absolute risk of cardiovascular disease is still higher for males. For additional details, we refer to Additional file 1: Tables S133–S134. Due to their low mortality and lack of cardiovascular diseases as inclusion criteria but strong links into the high-mortality region, we interpret clusters 112 (diabetes and hypertensive diseases) and 120 (diabetes, metabolic disorders and hypertensive diseases) as high-risk (cardiovascular) clusters, circled blue in Fig. 6. Their age profiles are shown in Fig. 3.

## Discussion

We presented a novel method to describe disease trajectories as observed over 17 years from a population-wide data set on hospital diagnoses. The method is based on classifying observed medical histories of patients using a small number of multimorbidity patterns (clusters) extracted from the data by means of a hierarchical clustering algorithm. Analysing the transition rates between the clusters, we create a sex- and age-stratified model of disease trajectories.

As expected, many of the clusters are specific to certain age groups; however, clusters populated by patients of similar age can differ significantly. Particularly, we show that elderly patients can be found in regions of low, medium and high mortality. The high-mortality region is characterised by the co-occurrence of multiple chronic conditions ranging from hypertension, obesity, diabetes, metabolic disorders, cardiovascular diseases and coronary heart diseases. This contrasts to the low-mortality region, where prevalences of these diseases are drastically lower and patients typically only suffer from diseases of the eye. Although morbidity and mortality attributable to cardiovascular diseases decreased in the last decade (with greater improvement in men), they remain the predominant causes of mortality in both sexes. On the other hand, NCDs like obesity, metabolic disorders, diabetes and renal diseases increased in men and women, partly due to population ageing. Clusters 129–131, which have the highest mortality, also include renal failure (N17–N19), and about 50% of the patients contained in them also have been diagnosed with diabetes mellitus (E10–E14). The comorbid prevalence of diabetes and renal disease and failure markedly increases cardiovascular morbidity and mortality. Patients with renal diseases hardly achieve the targets for blood pressure and glycemic control. However, the use of new classes of antidiabetic drugs may change the paradigm of unescapable cardiorenal risk in the future [37].

We identified two high-risk clusters via which many patients enter the high-cardiovascular mortality region. These clusters are characterised by a high prevalence of diseases like metabolic disorders, obesity, diabetes and hypertension, but low prevalence of cardiovascular diseases and low in-hospital mortality. Patients whose disease state is assigned to one of these clusters have a significantly increased risk of stepping into the high-mortality region within 1 year when compared to patients of the same age and sex but without recorded preconditions. This is in line with the findings that patients with diseases subsumed under the term “metabolic syndrome” have an almost threefold increased mortality because of cardiovascular or coronary heart disease in an 11.4-year follow-up [38]. Unfortunately, the diseases which characterise the high-risk clusters are often undetected and only diagnosed because of acute complications caused by the underlying disorders. For example, in Denmark, 35% of the patients with newly diagnosed diabetes featured diabetic complications around diagnosis [39]. As patients usually suffer from diabetes for many years before the diagnosis, screening and prevention programmes are necessary at least for subjects at high risk. It is highly recommended that patients with the clinical features hypertension, vascular diseases, dyslipidemia, prediabetes or abdominal obesity should as early as possible and all (other) subjects older than 45 years undergo diabetes screening at regular intervals [40]. Such procedure could help to identify more patients at risk earlier and to implement secondary prevention of long-term complications and assure guideline-based therapy.

In male patients aged 50–59 within the high-risk clusters, chronic obstructive pulmonary disease (COPD) significantly increases the risk of progressing into the high-cardiovascular mortality region compared to controls without COPD. The frequent comorbidity of COPD and cardiovascular diseases—especially in those aged under 65 years—has already been discussed in the literature. Besides smoking, also physical inactivity, air pollution and low maximally attained lung volume are thought to be shared risk factors. Yet, cardiovascular disease remains underrecognised and undertreated in patients with COPD [41]. Targeted screening for cardiovascular disease in patients with COPD, especially those mid-to-late middle-aged, has therefore been advocated for [41].

Women aged 60–69 in the high-risk clusters who proceeded into the high-cardiovascular mortality region within a 3-year follow-up have obtained diagnoses of obesity (E66) significantly more often than those who did not proceed. For men of the same age group in the high-risk clusters, we find the risk-increasing effect of obesity to be insignificant. Diabetic women who are overweight or obese have a significantly higher cardiovascular risk than their normal weight counterparts [42, 43]. When compared to females, increased risk for diabetic complications occurred at higher BMI levels in males [43].

Importantly, we find the relative risk of cardiovascular disease for patients associated with one of the high-risk clusters compared to healthy controls to be significantly larger in females than in males. Both diabetes and renal failure appear to attenuate the generally positive effect of female sex on life expectancy: these disorders seem to increase the risk in women to a greater extent, thereby equalising mortality risks [44–46]. This may be ascribed to sex-dimorphic changes in the risk factor burden and environmental factors. Recent studies showed that NCDs like obesity or diabetes are heterogenous entities with different outcomes among groups of patients and that sex and age can modify the risks. In case of obesity, the fat distribution pattern and central obesity-linked inflammation play a greater role than BMI class itself for cardiometabolic risk [47]. In regard to diabetes, even among the predominant class of type 2 diabetes, several specific diabetes subtypes with different outcome prediction are described and validated in European populations [48, 49]. Although our data set cannot distinguish subgroups based on genetics, biomarkers or clinical characteristics other than hospital diagnosis, we may provide additional evidence of the importance of personalised care to modify future risks. Among patients with type 2 diabetes, which comprises more than 90% of all diabetes cases, women with type 2 diabetes were shown to be more likely obese, hypertensive and have hypercholesterolaemia but were less likely to be prescribed lipid-lowering medication and antihypertensive drugs, especially if they had cardiovascular disease in comparison with men [50]. Moreover, it was shown that admissions for acute myocardial infarctions steadily increased in the last two decades especially in younger patients whose history of diabetes and hypertension increased in parallel [51]. The proportion attributable to younger women was particularly high who also had lower probability of receiving guideline-directed therapy.

The fact that we identify known risk factors for cardiovascular disease and confirm previously reported gender differences in the strength of their effect serves as a validation of our method. In Additional file 1: Tables S136–S137, we report further transitions between clusters which show a significant gender bias and may yield hypotheses for detailed follow-up analyses. For example, we note that men aged 20–30 with a history of drug abuse (F10–F19) have a higher risk of subsequently being diagnosed with depression when compared with women of the same age group. Male depression often remains undiagnosed because men are less likely to seek help [52] and tend to underreport symptoms of depression [53]. Furthermore, diagnostic criteria appear to better reflect symptoms of depressed women than their male counterparts who tend to display “atypical” symptoms such as alcohol and drug abuse as well as poor impulse control and risk taking [54]. Interestingly, this gender difference is reversed in the age group of 40–50-year-old patients, where the risk is higher for women. This may indicate that women at perimenopausal age are more vulnerable to the use of psychotropic drugs due to biological (endocrine) and psychosocial factors.

Our work is constrained by limitations in data availability, in particular, the lack of information on outpatient contacts, doctor visits, prescribed medications or death cases outside of hospitals. Moreover, the fact that the data was originally recorded for billing purposes means that diagnoses which did not lead to financial reimbursement were often not reported. Some diseases might therefore be strongly underrepresented in the data, in particular, lifestyle-related diagnoses like overweight or nicotine addiction. It is also important to bear in mind that the binary vectors characterising the state of patients at a given point in time encode their complete observed history of in-hospital diagnoses. This is due to the fact that the data quality does not allow to reliably infer which diseases a patient has been cured of. While for many diseases, the assumption that the patient’s health is still affected by them if they have been diagnosed with them in the past is well justified, other past diagnoses might be irrelevant. However, in the data-driven approach pursued here, we did not want to include any a priori knowledge about the acuteness of different conditions. Instead, we expect acute diseases which are independent of the other conditions and the further disease trajectories of patients to not influence the transition probabilities between clusters. Another limitation is that despite our choice of cohort, consisting of all patients who have not been assigned diagnoses with ICD-10 codes A00–N99 from 1997 to 2002, the assumption that all patients are free of any relevant preconditions will be wrong in some cases.

On a methodological level, the model might produce disease trajectories starting in cluster A, with disease *d* as an inclusion criterion, stepping to cluster B, where *d* is neither an exclusion nor an inclusion criterion, and from there to cluster *C*, where *d* is now an exclusion criterion, leading to a contradiction to the fact that the patient has previously visited cluster A and must therefore have been diagnosed with disease *d*.

We made the implicit assumption that the probability distribution of a patient’s health state in the next year does only depend on their sex, age and current health state; the pathway via which the patient has reached their current health state, as well as the time the patient has already spent in this health state is assumed to be irrelevant. The validity of this assumption remains to be seen.

To assess the quality of the trajectory model, we compared the performance of our model in terms of long-term predictions with two different benchmark models that assign patients to clusters based on single diseases (either the most recent or the least frequent one), see Additional file 1: Sections S4–S5. These models perform either comparable or only slightly worse than the DIVCLUS-T approach in terms of cluster inertia, which can be understood from the fact that they nevertheless capture disease–disease correlations (note that for a given cluster, the cluster-specific frequencies of other diseases are the marginal frequencies of the diseases computed over all patients fulfilling the criteria for that cluster). However, if we take the longitudinal component of the data into account and compare the statistics of simulated and real disease trajectories, we find that our approach clearly outperforms the benchmark models. This indicates that the issue of logically impossible trajectories does not substantially impact our results. Moreover, our results show that our multilayer network approach based on typical multimorbidity patterns provides a more adequate framework to capture the path-dependent nature of long-term disease trajectories compared to the single-disease benchmark models. It will be of interest to extend the current framework with additional clinical variables or to focus on more specific subsets of patients for a more precise identification of high-risk clusters for specific diseases.

## Supplementary information

**Additional file 1** Supplementary information. **Section S1.** Description of the algorithm used to cluster the data. **Section S2.** Explanation of the choice of the number of clusters. **Section S3.** Explanation of our notion of statistical robustness. **Section S4.** Description of our method to simulate patient disease trajectories. **Section S5.** Comparison of the performance of our clustering method with two benchmark methods. **Figure S1.** Two-dimensional histogram of the age distributions in the different clusters. **Figure S2.** Visualisation of the transition rates between each pair of clusters. **Figure S3.** Visualisation of the number of patients transitioning between each pair of clusters. **Figure S4.** Number of observations assigned to the different clusters. **Figure S5.** Two-dimensional histogram of the number of distinct diagnoses acquired by patients of the study cohort in the observation period from 2003 to 2014, depending on the 5-year age group they belong to in 2014. **Figure S6.** Distribution of the dates of birth of patients in the study cohort. **Figure S7.** Relative distribution of the number of clusters visited during the observation period for all people in the cohort, patients diagnosed with cerebrovascular diseases (I60–I69), malignant neoplasms (C00–C97) and mood [affective] disorders (F30–F39) during the observation period. **Figure S8.** Distribution of clusters occupied by patients at the end of the observation period (a). Distribution of clusters occupied by patients at the end of their lives, as forecasted by our multiplex network model of disease trajectories (b). **Figure S9.** Mean cluster inertia as a function of the number of clusters for original and uncorrelated data. **Table S1.** Definition of all blocks of ICD-10 codes considered in this work. **Tables S2–S132.** Inclusion and exclusion criteria of all clusters. **Tables S133–S134.** Absolute risk for patients in clusters 112 and 120 to step into the high cardiovascular mortality region, depending on age and sex, and relative risk compared with all other patients and patients in cluster 0 with the same age and sex. **Table S135.** Comparison of the performance of our approach to modeling patient trajectories based on clustering patients using DIVCLUS-T and two other benchmark algorithms. **Tables S136–S137**. Age–specific cluster transitions with significant gender bias.

## Data Availability

Data on sex- and age-stratified transition rates between the different clusters is available from the authors upon request.
