# Supplementary material for: High-risk multimorbidity patterns on the road to cardiovascular mortality
Source: BMC Med. 2020 Mar 10;18:44. doi: 10.1186/s12916-020-1508-1 (PMC7063814; doi:10.1186/s12916-020-1508-1)
Supplement: Supplementary file 1 — Additional file 1 Supplementary information. Section S1. Description of the algorithm used to cluster the data. Section S2. Explanation of the choice of the number of clusters. Section S3. Explanation of our notion of statistical robustness. Section S4. Description of our method to simulate patient disease trajectories. Section S5. Comparison of the performance of our clustering method with two benchmark methods. Figure S1. Two-dimensional histogram of the age distributions in the different clusters. Figure S2. Visualisation of the transition rates between each pair of clusters. Figure S3. Visualisation of the number of patients transitioning between each pair of clusters. Figure S4. Number of observations assigned to the different clusters. Figure S5. Two-dimensional histogram of the number of distinct diagnoses acquired by patients of the study cohort in the observation period from 2003 to 2014, depending on the 5-year age group they belong to in 2014. Figure S6. Distribution of the dates of birth of patients in the study cohort. Figure S7. Relative distribution of the number of clusters visited during the observation period for all people in the cohort, patients diagnosed with cerebrovascular diseases (I60–I69), malignant neoplasms (C00–C97) and mood [affective] disorders (F30–F39) during the observation period. Figure S8. Distribution of clusters occupied by patients at the end of the observation period (a). Distribution of clusters occupied by patients at the end of their lives, as forecasted by our multiplex network model of disease trajectories (b). Figure S9. Mean cluster inertia as a function of the number of clusters for original and uncorrelated data. Table S1. Definition of all blocks of ICD-10 codes considered in this work. Tables S2–S132. Inclusion and exclusion criteria of all clusters. Tables S133–S134. Absolute risk for patients in clusters 112 and 120 to step into the high cardiovascular mortality region, depending on age and sex, and relative risk [file 12916_2020_1508_MOESM1_ESM.pdf]

Supplementary Information for

High-risk multimorbidity patterns on the road to cardiovascular mortality.

Nina Haug, Carola Deischinger, Michael Gyimesi, Alexandra Kautzky-Willer,
Stefan Thurner and Peter Klimek

S1 Clustering algorithm

The divisive hierarchical clustering algorithm which we use in this work is called DIVCLUS-T, and was presented in [1]. It is similar to the well established Ward's minimum variance method [2] and the k-means algorithm [3, chap. 20] in that it aims to optimize the same objective function as these two clustering methods, but increases interpretability due to the fact that its results can be read like a decision tree.

The input data passed to the algorithm is in the form of a binary matrix $\mathbf{X} \in \{0,1\}^{M\times N}$, recording M observations of N different features. The element of $\mathbf{X}$ which stands in the ith row and jth column is denoted by $X_{i,j}$, where $1 \le i \le M$ and $1 \le j \le N$. We also denote for $1 \le i \le M$ the binary vector representing one particular observation by $\mathbf{x}^{(i)} = (X_{i,1}, \ldots, X_{i,N})$. In our application, one row of $\mathbf{X}$ corresponds to the health state of one patient, observed at a certain time, and each column corresponds to a particular block of diagnoses. For example, if column j represents the diagnosis block 'Diabetes Mellitus', then $X_{i,j} = 1$ if the patient represented by row i has been diagnosed with diabetes until the point in time the observation was made, and 0 otherwise. In our implementation of the algorithm, the user further specifies a binary vector $\mathbf{v} \in \{0,1\}^N$, a real, non-negative vector $\mathbf{w} \in [0,\infty)^N$, as well as two positive integers θ and K.

Starting from the root node, the algorithm iteratively constructs a binary tree where each node k represents one particular subset S_k of the total set of observations $S_0 = \{1,\ldots,M\}$, and $S_k \supset S_j$ if and only if node k is an ancestor of node j; the set S_0 is represented by the root node. To each node k is further associated a vector $\mathbf{p}^{(k)} = (p_1^{(k)}, \ldots, p_N^{(k)}) \in [0,1]^N$, defined as

$$\mathbf{p}^{(k)} = \frac{1}{|S_k|}\sum_{i\in S_k} \mathbf{x}^{(i)}. \tag{1}$$

Geometrically, $\mathbf{p}^{(k)}$ is the centroid of the $|S_k|$ binary vectors represented by node k, embedded into $\mathbb{R}^N$. For $1 \le j \le N$, the value $p_j^{(k)}$ is the probability that an observation, randomly selected from S_k, has feature j. The inertia of a node k is defined as

$$I_k = \sum_{i\in S_k} \|\mathbf{w} \circ (\mathbf{x}_i - \mathbf{p}^{(k)})\|_2^2 = |S_k|\mathbf{w} \cdot \left(\mathbf{p}^{(k)} \circ (\mathbf{1} - \mathbf{p}^{(k)})\right), \tag{2}$$

where '$\circ$' denotes the coefficientwise product, '$\cdot$' is the Euclidean scalar product, $\mathbf{1} = (1,\ldots,1)$ and $\|\cdot\|_2$ denotes the Euclidean norm. For a given feature $1 \le j \le N$, the value $p_j^{(k)}(1 - p_j^{(k)})$ is the variance of the set of points $\{\mathbf{x}_i \mid i \in S_k\}$, projected onto the jth axis. It can therefore be regarded as a measure for how homogeneous the observations belonging to S_k are with respect to feature j. The inertia I_k quantifies the overall inhomogeneity of the observations represented by note k, averaged over all the features, weighted according to the entries of $\mathbf{w}$. Note that our use of the letter $\mathbf{w}$ differs from [1], where weights were assigned to observations.

Let after any number of iterations of the algorithm node k be a leaf node of the tree constructed thus far. To simplify the notation, we drop the index k and let S be the set of observations represented by that node. Define for a given feature $1 \le j \le N$ the subsets $S_0^j = \{i \in S \mid X_{i,j} = 0\}$ and $S_1^j = \{i \in S \mid X_{i,j} = 1\}$. Hence, the sets S_1^j and S_0^j partition the set S into those observations which do or do not have feature j, respectively. Among the features $1 \le j \le N$ for which $v_j = 1$, the algorithm now selects the feature

$$j_0 = \underset{j\in J}{\operatorname{argmin}} \left(I_0^j + I_1^j\right), \tag{3}$$

where I_0^j and I_1^j are the inertias of S_1^j and S_0^j, respectively, and

$$J = \left\{1 \le j \le N \,\middle|\, v_j = 1 \wedge \min(|S_0^j|, |S_1^j|) > \theta\right\}. \tag{4}$$

Node k now becomes the parent node of two newly created leaf nodes representing the subsets $S_0^{j_0}$ and $S_1^{j_0}$, respectively. The feature j_0 is hence the feature used to split up node k; the binary vector $\mathbf{v}$ specifies which features are allowed to be used to split up nodes.

After a certain number of iterations, the set J is empty for each leaf node, and the algorithm therefore stops. Assume that the number of leaves of the generated tree is L and denote the sum of the inertias of all leaves of this tree by F. By removing two leaves of the tree with common parent node, we obtain a pruned tree of $L-1$ leaves where the parent node is now a leaf. Denote the sum of the inertias of all leaves of this new tree by F'. We remove the pair of nodes with common parent node such that the difference $F' - F$ is minimal. By repeating this procedure $L - K$ times, we arrive at a tree with K nodes. We denote the sets represented by the leaves of this tree by $C_1, \ldots, C_K$. They form a partition of the set $S_0 = \{1,\ldots,M\}$, and hence give a clustering of the observations contained in $\mathbf{X}$ into K disjoint subsets.

Suppose we are given a certain observation but do not know which cluster it belongs to. To determine the cluster membership of that observation, we proceed as follows. We ask whether the feature which was used to split up the root node is present in the observation or not, and, accordingly, we move on to the child node of the root which has or does not have this feature, respectively, and so forth, until we arrive at a leaf node (i.e., a cluster). The cluster membership of any observation can thus be determined by a finite sequence of binary questions. It is this property which distinguishes it from Ward's method and the k-means algorithm, and which increases the interpretability of the results obtained. However, this comes at the cost of restricting the set of possible cluster partitions, and therefore potentially less optimal solutions.

Specifically in this work, we set $\theta = 1000$, $\mathbf{v} = (1, 1, \ldots, 1)$, and the entries of the weight vector $\mathbf{w} = (w_1, \ldots, w_N)$ are set as

$$w_j = -\lambda \ln(p_j) \quad \text{for } 1 \leq j \leq N, \tag{5}$$

where $\lambda = N / \sum_{j=1}^{N} \ln(p_j)$, with $p_j = \frac{1}{M} \sum_{i=1}^{M} X_{i,j}$ being the probability that a randomly selected patient of the cohort, observed at a randomly selected time in T, has been diagnosed with disease j so far. The quantity on the right of Eq. (5) is known as the inverse document frequency (idf), a commonly used measure in text analytics [4]. The idea behind idf is that terms appearing less frequently in a corpus of documents are more distinctive to a text which contains them. In our context, documents correspond to observations and words correspond to individual diseases.

The objective function aimed to be minimized by DIVCLUS-T is

$$F = \sum_{k=1}^{K} I_k, \tag{6}$$

where for $1 \leq k \leq K$, I_k is the inertia of cluster k. In the case where $\mathbf{w} = (1, \ldots, 1)$, F is the same objective function aimed to be minimized by Ward's method and the k-means algorithm.

Note that DIVCLUS-T, as described here, can also be used for prediction purposes. For example, if we set $\mathbf{v} = (1, 1, \ldots, 1, 0)$ and $\mathbf{w} = (0, 0, \ldots, 0, 1)$, then the algorithm generates a decision tree predicting the feature represented by the last column of $\mathbf{X}$ based on all other features.

S2 Selection of the number of clusters

As in the last section, we denote by F the sum of the inertias of the leaf nodes of a clustering tree. The mean cluster inertia of a clustering tree of K leaves is defined as $f = \frac{F}{K}$. In order to find out whether our set of data points admits a natural partitioning into a certain number of clusters, in Fig. S9 we plot the mean cluster inertia f against the number K of clusters. In case such a natural partitioning exists, one expects that the mean cluster inertia decreases strongly as the number of clusters increases from one to the natural number of clusters in the data, and less strongly as the number of clusters increases further. This plot shows that the mean cluster inertia decreases strongly for small values of the number of clusters and less strongly for larger numbers of clusters.

In order to quantify the effect of correlations in the data, as in the previous section we compute for each disease block j the marginal probability $p_j = \frac{1}{M} \sum_{i=1}^{M} X_{i,j}$. Then we create a ranking $r : \{1, \ldots, N\} \to \{1, \ldots, N\}$ such that $w_{r(i)} p_{r(i)} (1 - p_{r(i)}) > w_{r(j)} p_{r(j)} (1 - p_{r(j)})$ if $i < j$. For a given number of clusters K we compute

$$\tilde{f} = \sum_{d=K}^{N} w_{r(d)} p_{r(d)} (1 - p_{r(d)}). \tag{7}$$

The value $\tilde{f}$ is the expectation value of the mean cluster inertia obtained by randomly shuffling the ones of the matrix $\mathbf{X}$ independently in each column. The difference $\tilde{f} - f$ indicates how strongly different diseases in the data are correlated. The pink line in Fig. S9 shows the value of $\tilde{f}$ as a function of K. We see that there is a clear effect of correlations between different diagnoses in the data, which is made use of by our clustering algorithm.

The choice $K = 132$ is made as a trade-off between statistical robustness and homogeneity of the clusters. Moreover, this value enables us to directly compare the performance of the algorithm with two simple approaches used as benchmarks, as discussed in section S5.

S3 Robustness of our results

As explained in the methods section of the main article, the weight $q_{s,g,i,j}$ is the rate at which patients of sex g and age group a step from cluster i to cluster j. This rate is computed by dividing the number $s_{g,a,i,j}$ of

steps of patients with sex g from cluster i to cluster j in layer a by the number of observations $\sum_{j=1}^{K} s_{g,a,i,j}$ (the size) of cluster i in layer a for sex g. If we consider our dataset as a realization of a certain stochastic process, then $q_{g,a,i,j}$ is an empirical estimation of the transition probability $\hat{q}_{g,a,i,j}$ inherent to this process. We call an edge weight robust if the width of the 95% confidence interval for the estimation of $\hat{q}_{g,a,i,j}$ is smaller than $\lambda\, q_{g,a,i,j}$, where $\lambda = 0.2$. To check if a given edge weight $q_{g,i,j}$ is robust, we proceed as follows.

Let g, a, i and j be fixed and simplify the notation to $p = q_{g,a,i,j}$, $\hat{p} = \hat{q}_{g,a,i,j}$ and $s = \sum_{j=1}^{K} s_{g,a,i,j}$. The empirical probability is a random variable

$$p = \frac{1}{s}\sum_{i=1}^{s} X_i, \tag{8}$$

where $X_1, \ldots, X_s$ are iid random variables with $X_1 \sim \text{Bernoulli}(\hat{p})$. It follows from the central limit theorem that for $s \to \infty$,

$$\sqrt{\frac{s}{\hat{p}(1-\hat{p})}}\,(p - \hat{p}) \xrightarrow{d} \mathcal{N}(0,1), \tag{9}$$

where $\xrightarrow{d}$ denotes convergence in distribution. This means that for $\delta > 0$,

$$\lim_{s\to\infty} \mathbb{P}\Big(\hat{p} \in \Big[p - \delta\sqrt{\frac{\hat{p}(1-\hat{p})}{s}}, p + \delta\sqrt{\frac{\hat{p}(1-\hat{p})}{s}}\Big]\Big) = 2C(\delta) - 1, \tag{10}$$

where $C(\cdot)$ is the cumulative distribution function of the standard normal distribution. For sufficiently large s, the condition that

$$\delta\sqrt{\frac{\hat{p}(1-\hat{p})}{s}} \le \lambda p$$

is equivalent to $p \ge \delta^2/(s\lambda^2)$.

For $\delta \simeq 1.96$, $2C(\delta) - 1 = 0.95$. Our condition for an edge to be robust is therefore that

$$q_{g,a,i,j} \ge \left(\frac{1.96}{0.2}\right)^2 \cdot \frac{1}{s} \simeq \frac{96}{s}. \tag{11}$$

S4 Simulating disease trajectories

We can simulate patient disease trajectories as random walks on the multilayer network of disease clusters. Assume we are given a patient of sex g and age group a and want to simulate their future disease trajectory based on their present disease state. We first determine the layer of the multilayer network matching their sex and age. Their present position in this layer is then determined as the cluster whose inclusion and exclusion criteria match their disease history. Let the patient be in cluster k at present. We randomly choose the position of the patient in the next year, such that with probability $q_{g,a',k,j}$, their next position is in cluster j, where a' is determined from the age of the patient in the next year. Repeating this procedure, we arrive at a stochastically simulated long-term disease trajectory. Since during the simulation, the network layer on which the patient is moving is updated according to their age progression, the model takes into account effects of ageing. Fig. S8 (a) shows how the cohort patients distribute over the different clusters at the end of the observation period. Simulating the further disease progression of the cohort patients after the end of the observation period, we arrive at Fig. S8 (b), which shows the forecasted distribution of the cohort patients over the clusters at the end of their lives. To assess the quality of the model, we compute several metrics for comparing disease frequencies according to the simulated trajectories with the data, and compare these metrics with those of two benchmark models, see section S5.

S5 Performance of the method and comparison with benchmarks

To quantify how well the cluster transitions shown in Fig. 6 of the main text characterize the progression of actual multimorbid health states, we evaluate the performance of our method for simulating patient trajectories in terms of several metrics and compare these metrics to two benchmark models that replace DIVCLUS-T by a clustering scheme which assigns patients to clusters based on a single disease.

We denote for $1 \le d \le N$ by p_d the probability that a randomly selected patient of the study cohort has been assigned a diagnosis from the ICD-10 code block d until the end of the observation period. The total disease burden of the study cohort at the end of the observation period is given by

$$N_{\text{obs}} = M \cdot \|\mathbf{p}\|_1, \tag{12}$$

where $M = 5,112,811$ is the size of the cohort and $\mathbf{p} = (p_1, \ldots, p_N)$. Here, for $p \geq 1$, $\|\|_p$ denotes the L^p norm, which, for a vector $\mathbf{x} = (x_1, \ldots, x_L)$, is defined as

$$\|\mathbf{x}\|_p = \left(\sum_{\ell=1}^{L} x_\ell^p \right)^{\frac{1}{p}}. \tag{13}$$

Let $\hat{p}_d$ be the marginal probability for a randomly selected patient to have been diagnosed with disease d until the end of the observation period according to our random walk model. The vector $\hat{\mathbf{p}} = (\hat{p}_1, \ldots, \hat{p}_N)$ is computed as

$$\hat{\mathbf{p}} = \sum_{k=1}^{K} \hat{c}_k \mathbf{p}^{(k)}, \tag{14}$$

where $\mathbf{p}^{(k)} = (p_k^{(1)}, \ldots, p_k^{(N)})$, with $p_d^{(k)}$ for $1 \leq d \leq N$ being the probability for a randomly selected patient in cluster k to have disease d; $\hat{c}^{(k)}$ is the probability that a randomly selected patient of the cohort is assigned to cluster k at the end of the observation period. The total disease burden of the study population according to our model is

$$N_{\exp} = M \cdot \|\hat{\mathbf{p}}\|_1. \tag{15}$$

The quantity $\|\mathbf{w} \circ \hat{\mathbf{p}}\|_1 / \|\mathbf{w} \circ \mathbf{p}\|_1$, where the entries of the vector $\mathbf{w} = (w_1, w_2, \ldots, w_N)$ are defined in Eq. (5), serves us as a measure for how well our model reflects the observed total disease burden of our study cohort at the end of the observation period. We also compute the quantities $\|\Delta\mathbf{p}\|_1$ and $\|\Delta\mathbf{p}\|_2$, where $\Delta\mathbf{p} = \mathbf{w} \circ (\mathbf{p} - \hat{\mathbf{p}})$. Together with the mean cluster inertia f, these quantities measure the performance of our algorithm in describing and predicting patient trajectories.

Benchmark method 1 consists in clustering the health states of patients according to their most recent main diagnosis. For example, if a patient had one hospital stay with main diagnosis 'Malignant neoplasms' in 2008, then their health state at the end of that year is assigned to the cluster where all patients had malignant neoplasms as their most recent main diagnosis. If a patient received more than one main diagnosis in a given year, then their health state at the end of this year is assigned to the cluster of patients with the less frequent main diagnosis. An additional cluster contains all patients who have not had any diagnoses yet.

Benchmark method 2 consists in clustering patients according to the rarest disease they have been diagnosed with so far. For example, if a patient has been diagnosed with diagnoses from the block 'Hypertensive diseases' and 'Ischaemic heart diseases', then they are assigned to the cluster of patients who have 'Ischaemic heart diseases' as their rarest disease, because diagnoses of this block are rarer than 'Hypertensive diseases' diagnoses. As for Benchmark method 1, an additional cluster contains all patients who have not had any diagnoses yet.

The quantities defined above are compared in table S135. While both with DIVCLUS-T and Benchmark method 2 we attain a mean cluster inertia of 0.48, DIVCLUS-T clearly outperforms both benchmark methods in terms of the ability to model future disease burdens, as quantified by the other metrics. Benchmark method 1 performs worse than DIVCLUS-T in all metrics.

S6 Figures

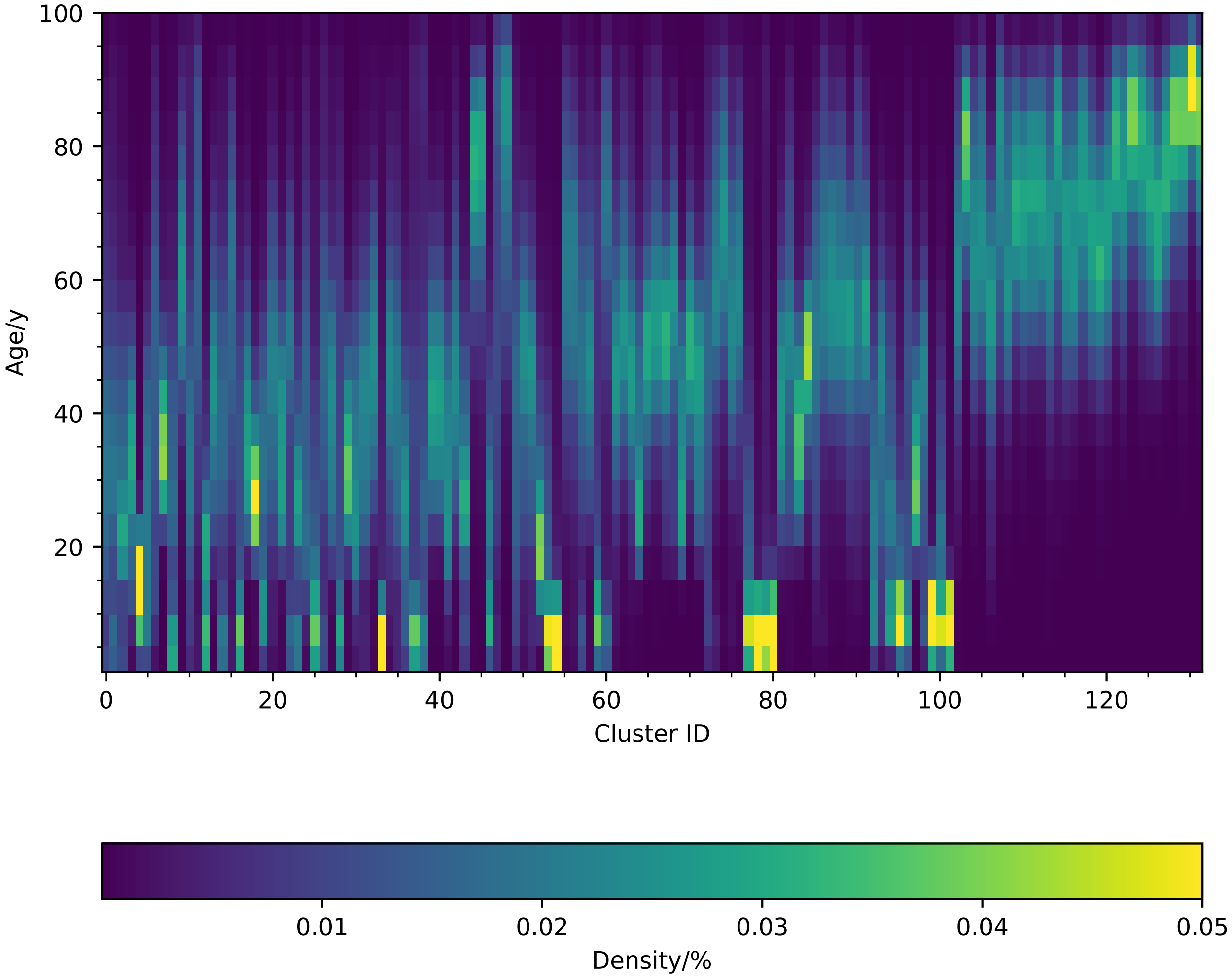

Figure S1: Two-dimensional histogram of the age distribution of patients in the different clusters.

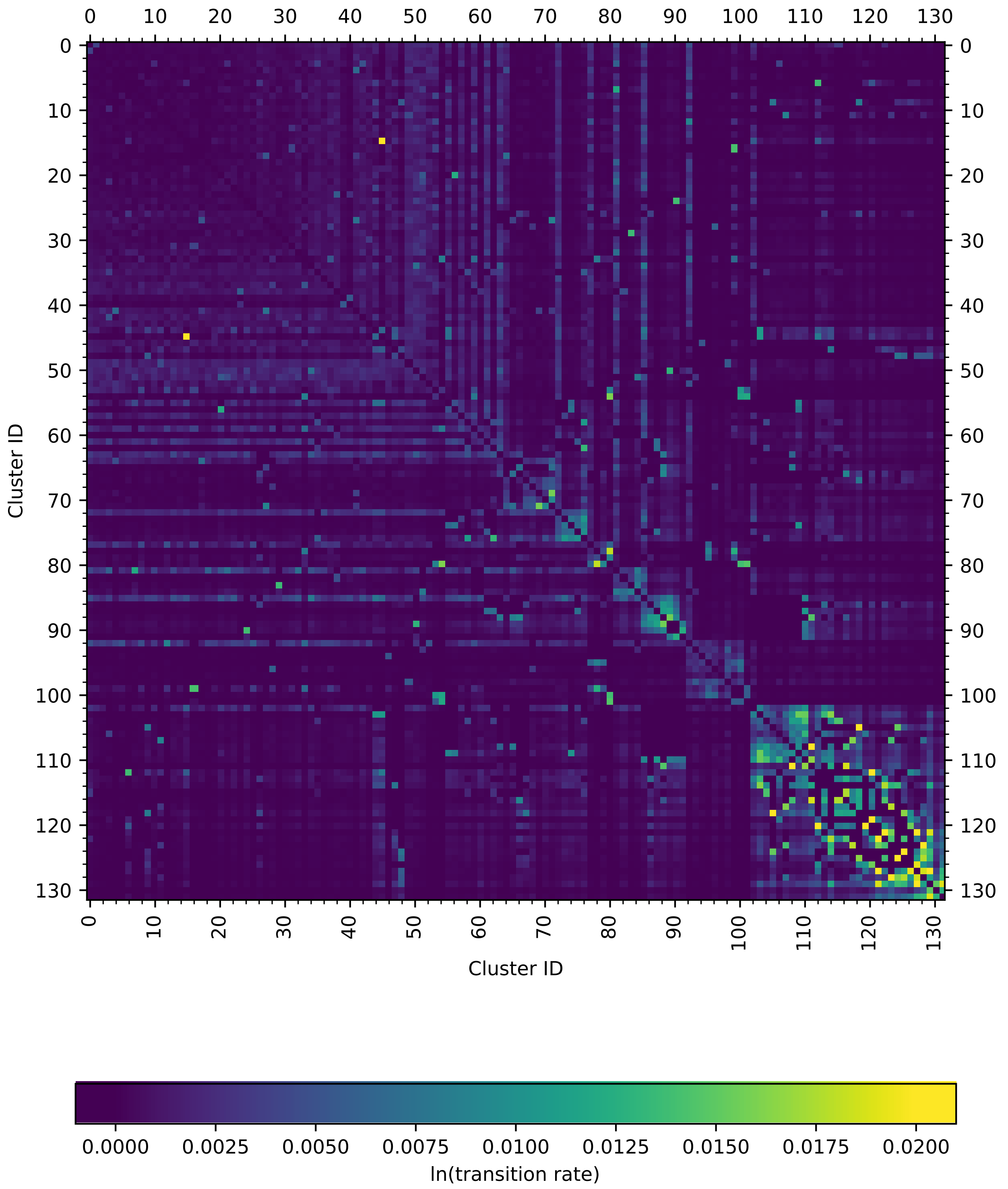

Figure S2: Visualisation of the transition rates between each pair of clusters. The image consists of 132 × 132 pixels, each representing one pair of clusters. The pixel in the jth row and kth column is coloured according to the rate at which patients transition between cluster j and cluster k. For better visibility, the pixels on the diagonal from the upper left to the lower right corner of the image, which represent self-loops, have been shaded dark.

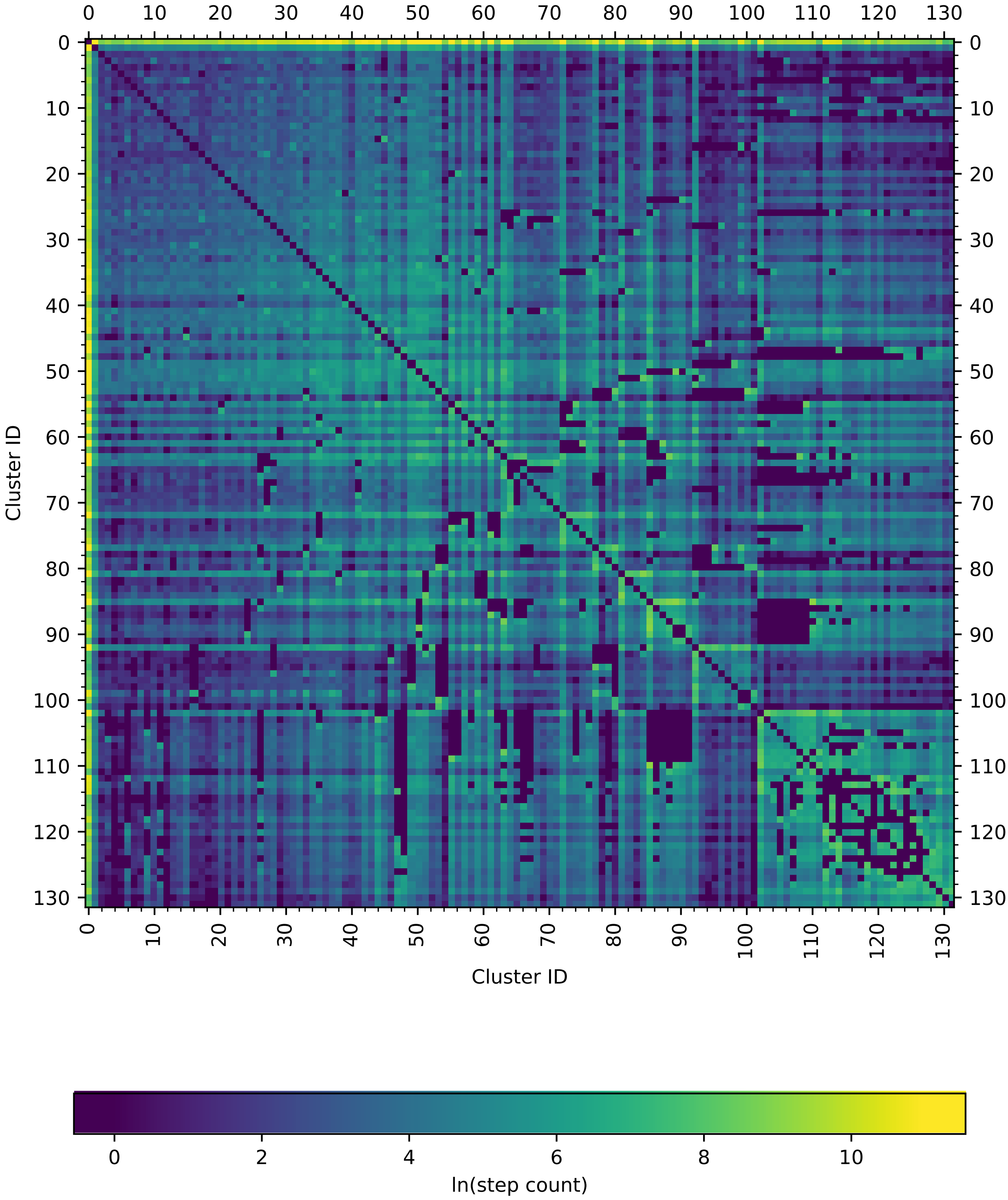

Figure S3: Visualisation of the number of patients transitioning between each pair of clusters. The image consists of 132 × 132 pixels, each representing one pair of clusters. The pixel in the jth row and kth column is coloured according to the number of patients who step from cluster j to cluster k or from cluster k to cluster j. For better visibility, the pixels on the diagonal from the upper left to the lower right corner of the image, which represent self-loops, have been shaded dark.

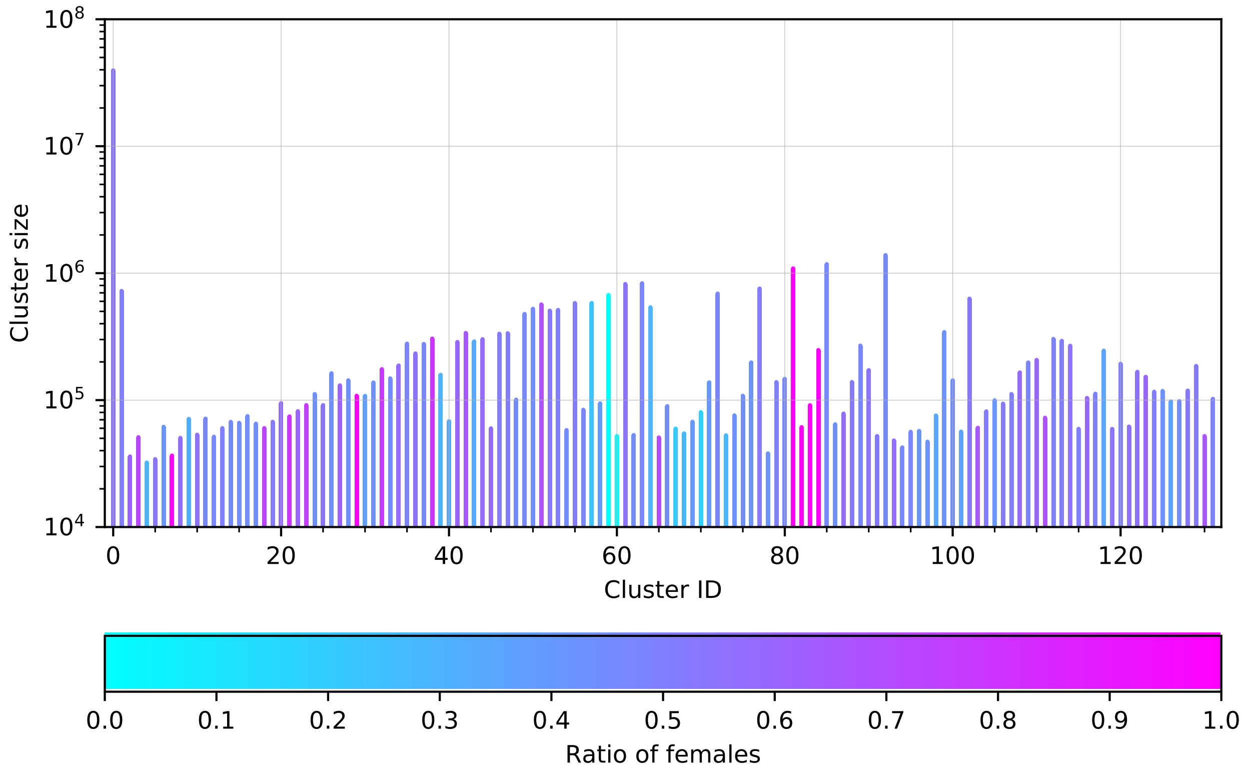

Figure S4: Number of observations assigned to the different clusters. The bars are color coded according to the ratio of females within the corresponding cluster.

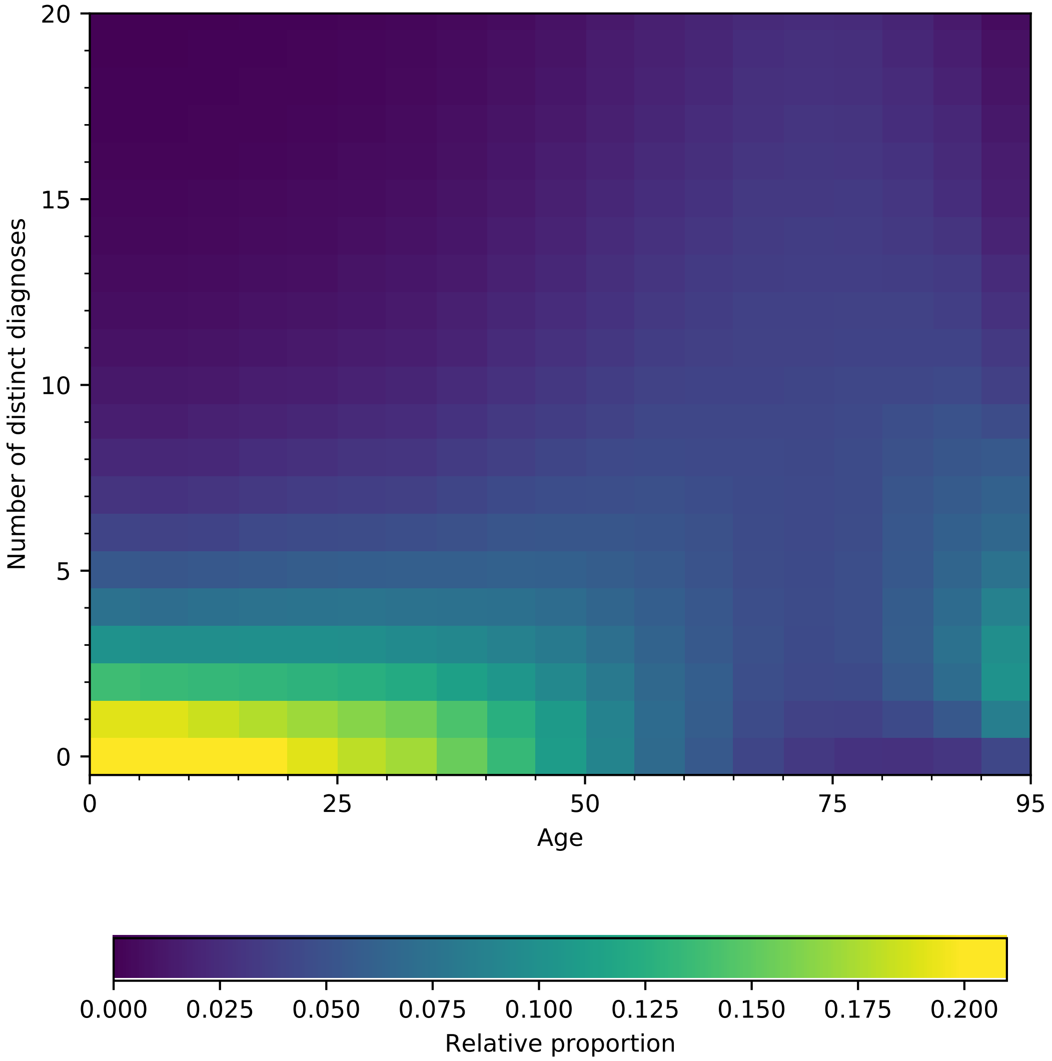

;

Figure S5: Two-dimensional histogram of the number of distinct diagnoses acquired by patients of the study cohort in the observation period from 2003 to 2014, depending on the 5-year age group they belong to in 2014. The data is normalized such that values sum to one separately for each age group.

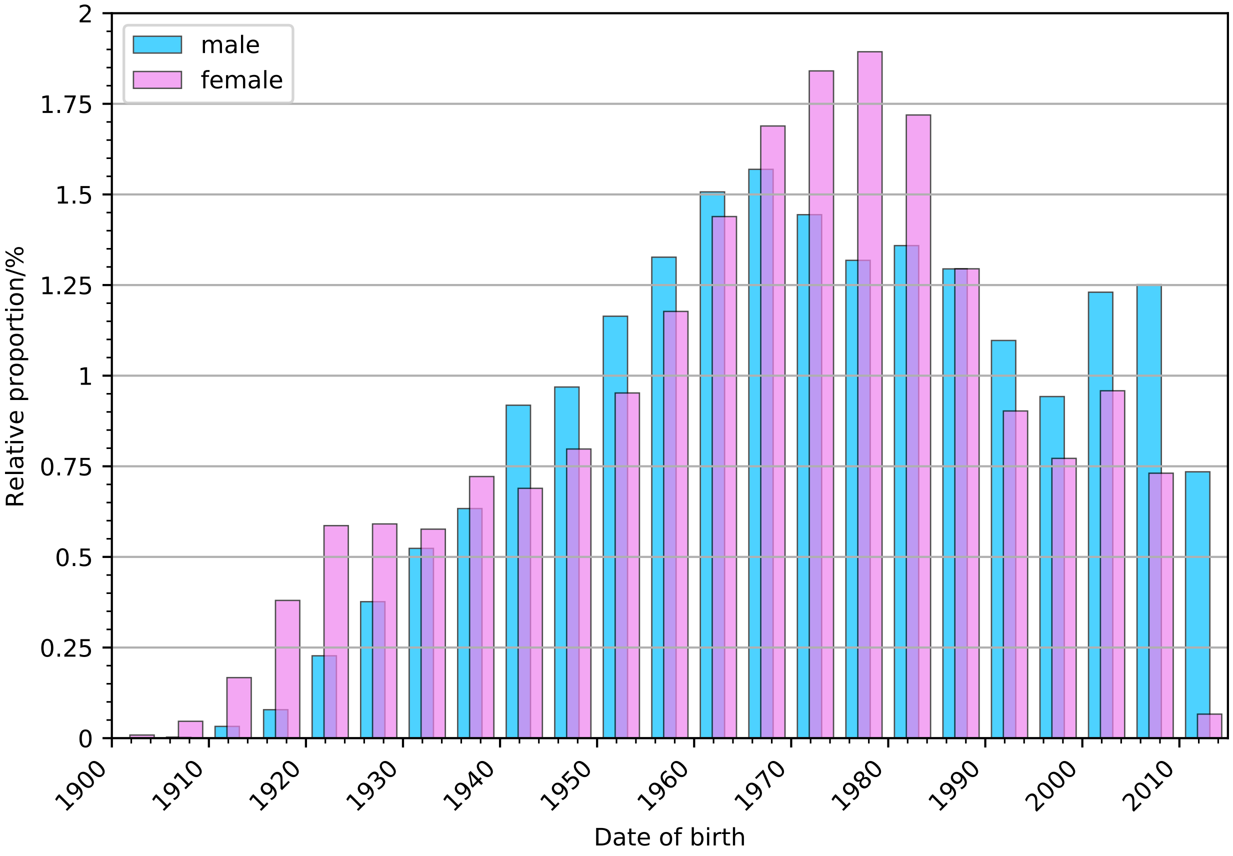

Figure S6: Distribution of the dates of birth of patients in the study cohort. The data is normalized such that the bar heights for males and females sum to one separately.

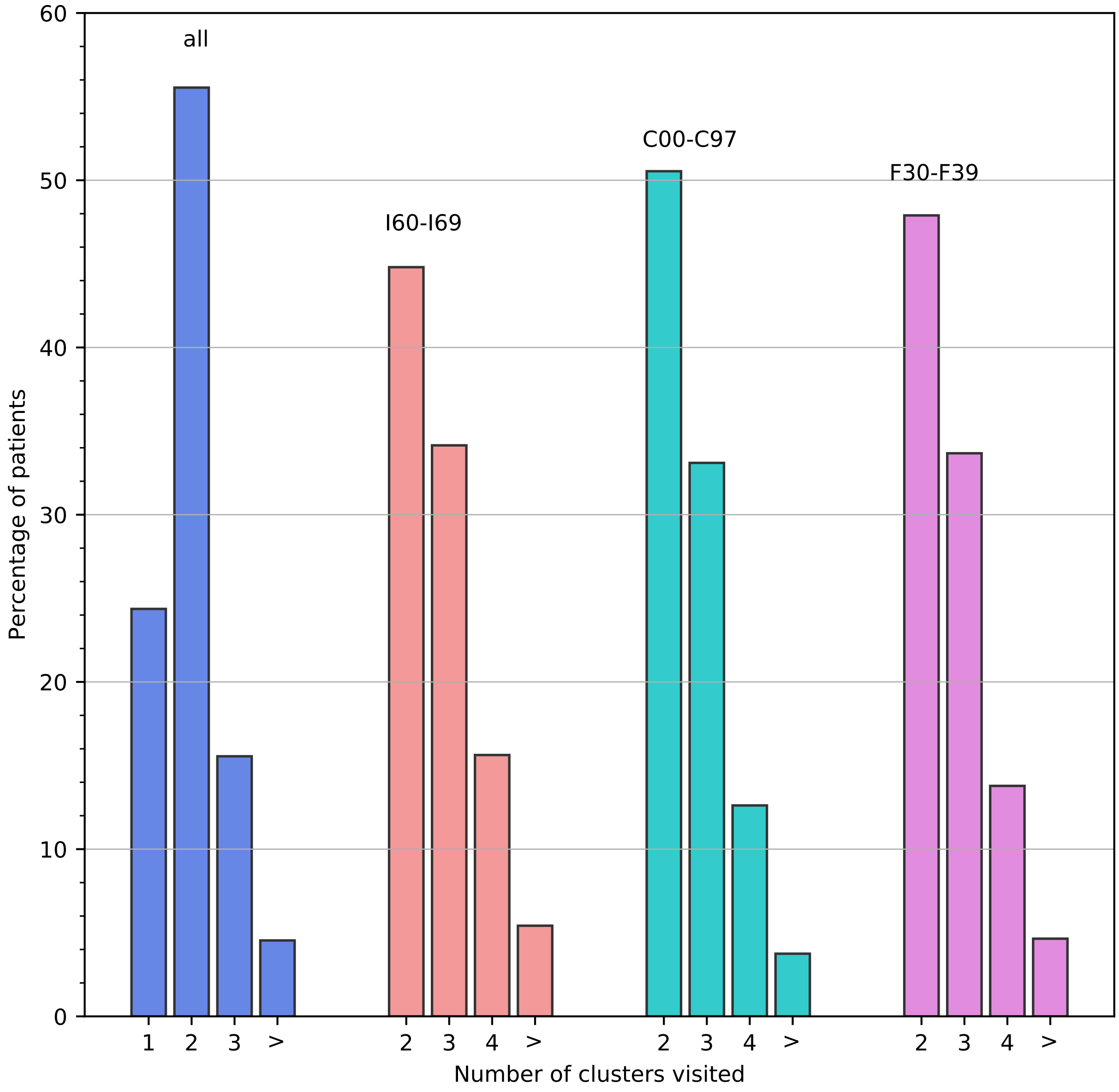

Figure S7: Relative distribution of the number of clusters visited during the observation period for all people in the cohort (blue), patients diagnosed with cerebrovascular diseases (red, I60–I69), malignant neoplasms (green, C00–C97) and mood [affective] disorders (magenta, F30–F39) during the observation period. Note the different labelling of the horizontal axis in the leftmost histogram.

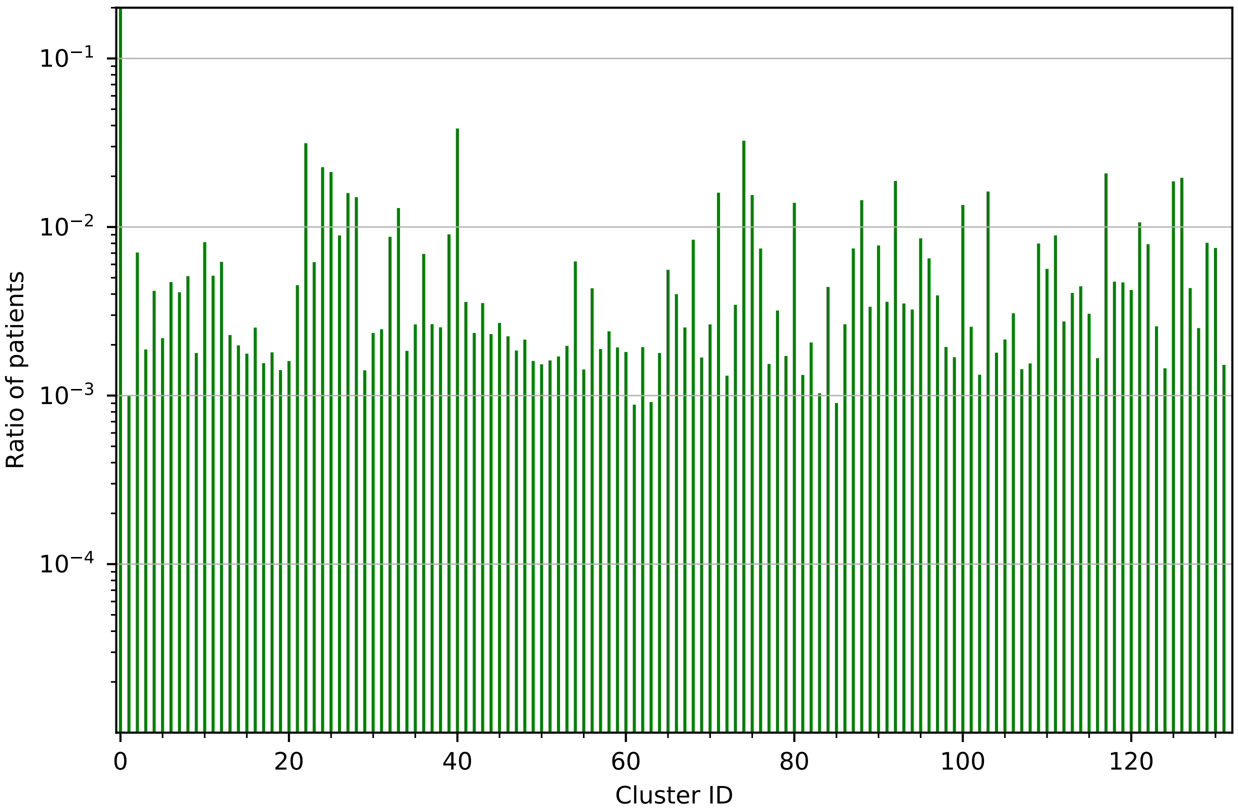

(a)

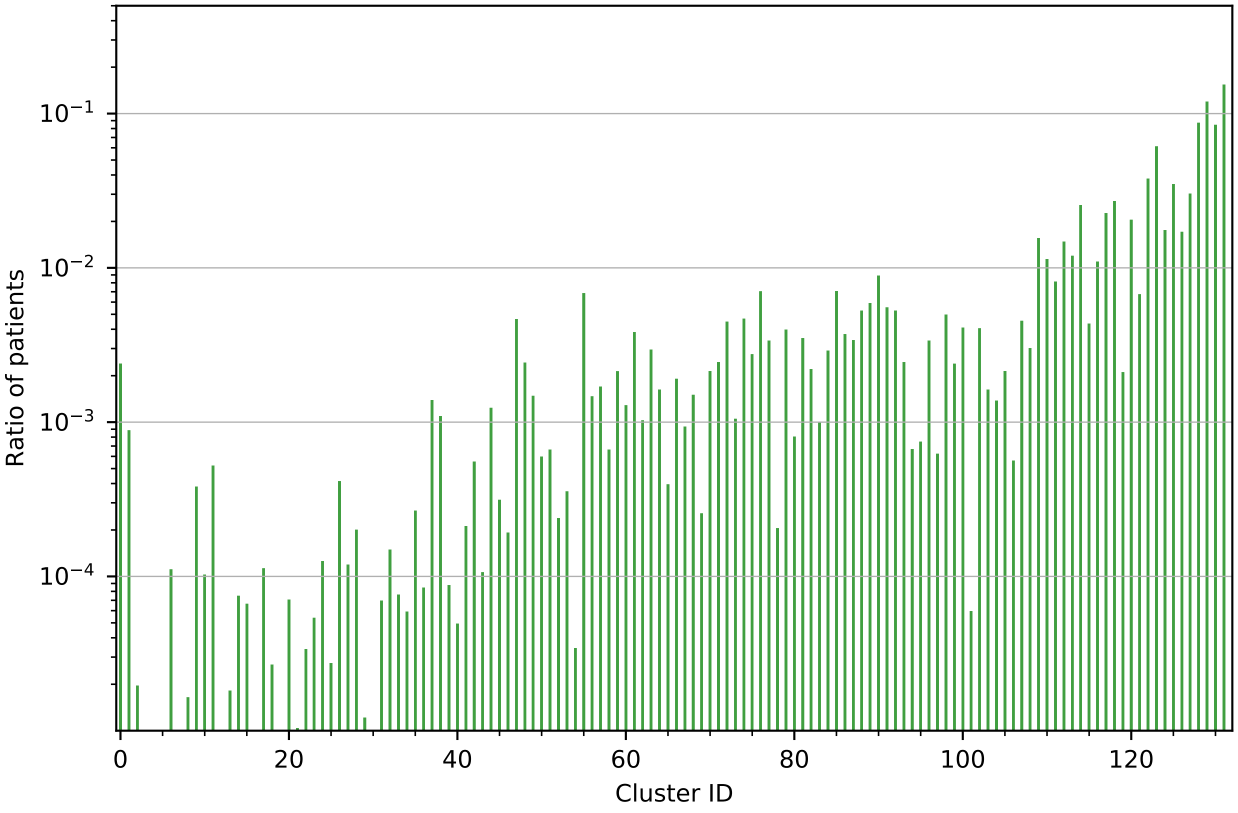

(b)

Figure S8: Distribution of clusters occupied by patients at the end of the observation period (a). Distribution of clusters occupied by patients at the end of their lives, as forecasted by our multiplex network model of disease trajectories (b).

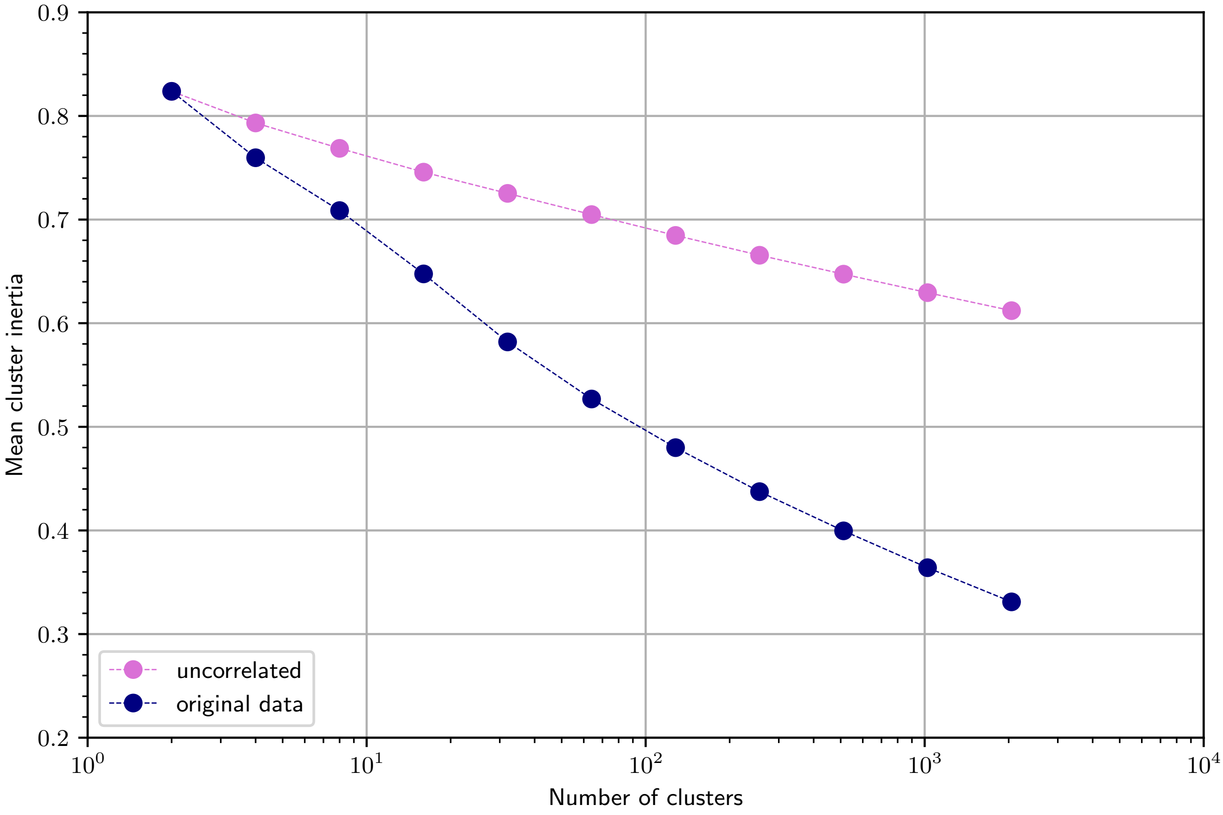

Figure S9: Mean cluster inertia as a function of the number of clusters for original and uncorrelated data.

S7 Tables

The following table lists all the diagnosis blocks considered in this work.

ID	ICD-10	Description
0	A00-A09	Intestinal infectious diseases
1	A15-A19	Tuberculosis
2	A20-A28	Certain zoonotic bacterial diseases
3	A30-A49	Other bacterial diseases
4	A50-A64	Infections with a predominantly sexual mode of transmission
5	A65-A69	Other spirochaetal diseases
6	A70-A74	Other diseases caused by chlamydiae
7	A75-A79	Rickettsioses
8	A80-A91	Viral infections of the central nervous system
9	A92-A99	Arthropod-borne viral fevers and viral haemorrhagic fevers
10	B00-B09	Viral infections characterized by skin and mucous membrane lesions
11	B15-B19	Viral hepatitis
12	B20-B24	Human immunodeficiency virus [HIV] disease
13	B25-B34	Other viral diseases
14	B35-B49	Mycoses
15	B50-B64	Protozoal diseases
16	B65-B83	Helminthiases
17	B85-B89	Pediculosis, acariasis and other infestations
18	B90-B94	Sequelae of infectious and parasitic diseases
19	B95-B97	Bacterial, viral and other infectious agents
20	B99-B99	Other infectious diseases
21	C00-C97	Malignant neoplasms
22	D00-D09	In situ neoplasms
23	D10-D36	Benign neoplasms
24	D37-D48	Neoplasms of uncertain or unknown behaviour
25	D50-D53	Nutritional anaemias
26	D55-D59	Haemolytic anaemias
27	D60-D64	Aplastic and other anaemias
28	D65-D69	Coagulation defects, purpura and other haemorrhagic conditions
29	D70-D77	Other diseases of blood and blood-forming organs
30	D80-D89	Certain disorders involving the immune mechanism
31	E00-E07	Disorders of thyroid gland
32	E10-E14	Diabetes mellitus
33	E15-E16	Other disorders of glucose regulation and pancreatic internal secretion
34	E20-E35	Disorders of other endocrine glands
35	E40-E46	Malnutrition
36	E50-E64	Other nutritional deficiencies
37	E65-E68	Obesity and other hyperalimentation
38	E70-E90	Metabolic disorders
39	F00-F09	Organic, including symptomatic, mental disorders
40	F10-F19	Mental and behavioral disorders due to psychoactive substance use
41	F20-F29	Schizophrenia, schizotypal and delusional disorders
42	F30-F39	Mood [affective] disorders
43	F40-F48	Neurotic, stress-related and somatoform disorders
44	F50-F59	Behavioral syndromes associated with physiological disturbances and physical factors
45	F60-F69	Disorders of adult personality and behaviour
46	F70-F79	Mental retardation
47	F80-F89	Disorders of psychological development
48	F90-F98	Behavioral and emotional disorders with onset usually occurring in childhood and adolescence
49	F99-F99	Unspecified mental disorder
50	G00-G09	Inflammatory diseases of the central nervous system
51	G10-G13	Systemic atrophies primarily affecting the central nervous system
52	G20-G26	Extrapyramidal and movement disorders
53	G30-G32	Other degenerative diseases of the nervous system
54	G35-G37	Demyelinating diseases of the central nervous system
55	G40-G47	Episodic and paroxysmal disorders
56	G50-G59	Nerve, nerve root and plexus disorders
57	G60-G64	Polyneuropathies and other disorders of the peripheral nervous system
58	G70-G73	Diseases of myoneural junction and muscle
59	G80-G83	Cerebral palsy and other paralytic syndromes
60	G90-G99	Other disorders of the nervous system

ID	ICD-10	Description
61	H00-H06	Disorders of eyelid, lacrimal system and orbit
62	H10-H13	Disorders of conjunctiva
63	H15-H22	Disorders of sclera, cornea, iris and ciliary body
64	H25-H28	Disorders of lens
65	H30-H36	Disorders of choroid and retina
66	H40-H42	Glaucoma
67	H43-H45	Disorders of vitreous body and globe
68	H46-H48	Disorders of optic nerve and visual pathways
69	H49-H52	Disorders of ocular muscles, binocular movement, accommodation and refraction
70	H53-H54	Visual disturbances and blindness
71	H55-H59	Other disorders of eye and adnexa
72	H60-H62	Diseases of external ear
73	H65-H75	Diseases of middle ear and mastoid
74	H80-H83	Diseases of inner ear
75	H90-H95	Other disorders of ear
76	I00-I02	Acute rheumatic fever
77	I05-I09	Chronic rheumatic heart diseases
78	I10-I15	Hypertensive diseases
79	I20-I25	Ischaemic heart diseases
80	I26-I28	Pulmonary heart disease and diseases of pulmonary circulation
81	I30-I52	Other forms of heart disease
82	I60-I69	Cerebrovascular diseases
83	I70-I79	Diseases of arteries, arterioles and capillaries
84	I80-I89	Diseases of veins, lymphatic vessels and lymph nodes, not elsewhere classified
85	I95-I99	Other and unspecified disorders of the circulatory system
86	J00-J06	Acute upper respiratory infections
87	J09-J18	Influenza and pneumonia
88	J20-J22	Other acute lower respiratory infections
89	J30-J39	Other diseases of upper respiratory tract
90	J40-J47	Chronic lower respiratory diseases
91	J60-J70	Lung diseases due to external agents
92	J80-J84	Other respiratory diseases principally affecting the interstitium
93	J85-J86	Suppurative and necrotic conditions of lower respiratory tract
94	J90-J94	Other diseases of pleura
95	J95-J99	Other diseases of the respiratory system
96	K00-K14	Diseases of oral cavity, salivary glands and jaws
97	K20-K31	Diseases of oesophagus, stomach and duodenum
98	K35-K38	Diseases of appendix
99	K40-K46	Hernia
100	K50-K52	Noninfective enteritis and colitis
101	K55-K63	Other diseases of intestines
102	K65-K67	Diseases of peritoneum
103	K70-K77	Diseases of liver
104	K80-K87	Disorders of gallbladder, biliary tract and pancreas
105	K90-K93	Other diseases of the digestive system
106	L00-L08	Infections of the skin and subcutaneous tissue
107	L10-L14	Bullous disorders
108	L20-L30	Dermatitis and eczema
109	L40-L45	Papulosquamous disorders
110	L50-L54	Urticaria and erythema
111	L55-L59	Radiation-related disorders of the skin and subcutaneous tissue
112	L60-L75	Disorders of skin appendages
113	L80-L99	Other disorders of the skin and subcutaneous tissue
114	M00-M25	Arthropathies
115	M30-M36	Systemic connective tissue disorders
116	M40-M54	Dorsopathies
117	M60-M79	Soft tissue disorders
118	M80-M94	Osteopathies and chondropathies
119	M95-M99	Other disorders of the musculoskeletal system and connective tissue
120	N00-N08	Glomerular diseases
121	N10-N16	Renal tubulo-interstitial diseases
122	N17-N19	Renal failure
123	N20-N23	Urolithiasis
124	N25-N29	Other disorders of kidney and ureter
125	N30-N39	Other diseases of urinary system
126	N40-N51	Diseases of male genital organs
127	N60-N64	Disorders of breast
128	N70-N77	Inflammatory diseases of female pelvic organs
129	N80-N98	Noninflammatory disorders of female genital tract
130	N99-N99	Other disorders of the genitourinary system

Table S1: List of all diagnosis blocks considered in this work.

Intestinal infectious diseases (A00-A09)	✗
Other bacterial diseases (A30-A49)	✗
Infections with a predominantly sexual mode of transmission (A50-A64)	✗
Viral infections characterized by skin and mucous membrane lesions (B00-B09)	✗
Other viral diseases (B25-B34)	✗
Malignant neoplasms (C00-C97)	✗
In situ neoplasms (D00-D09)	✗
Benign neoplasms (D10-D36)	✗
Neoplasms of uncertain or unknown behaviour (D37-D48)	✗
Disorders of thyroid gland (E00-E07)	✗
Diabetes mellitus (E10-E14)	✗
Obesity and other hyperalimentation (E65-E68)	✗
Metabolic disorders (E70-E90)	✗
Mental and behavioural disorders due to psychoactive substance use (F10-F19)	✗
Schizophrenia, schizotypal and delusional disorders (F20-F29)	✗
Mood [affective] disorders (F30-F39)	✗
Neurotic, stress-related and somatoform disorders (F40-F48)	✗
Behavioural and emotional disorders with onset usually occurring in childhood and adolescence (F90-F98)	✗
Episodic and paroxysmal disorders (G40-G47)	✗
Nerve, nerve root and plexus disorders (G50-G59)	✗
Disorders of eyelid, lacrimal system and orbit (H00-H06)	✗
Disorders of lens (H25-H28)	✗
Disorders of choroid and retina (H30-H36)	✗
Disorders of ocular muscles, binocular movement, accommodation and refraction (H49-H52)	✗
Diseases of middle ear and mastoid (H65-H75)	✗
Diseases of inner ear (H80-H83)	✗
Other disorders of ear (H90-H95)	✗
Hypertensive diseases (I10-I15)	✗
Ischaemic heart diseases (I20-I25)	✗
Other forms of heart disease (I30-I52)	✗
Cerebrovascular diseases (I60-I69)	✗
Diseases of veins, lymphatic vessels and lymph nodes, not elsewhere classified (I80-I89)	✗
Other and unspecified disorders of the circulatory system (I95-I99)	✗
Acute upper respiratory infections (J00-J06)	✗
Influenza and pneumonia (J09-J18)	✗
Other acute lower respiratory infections (J20-J22)	✗
Other diseases of upper respiratory tract (J30-J39)	✗
Chronic lower respiratory diseases (J40-J47)	✗
Diseases of oral cavity, salivary glands and jaws (K00-K14)	✗
Diseases of oesophagus, stomach and duodenum (K20-K31)	✗
Diseases of appendix (K35-K38)	✗
Hernia (K40-K46)	✗
Noninfective enteritis and colitis (K50-K52)	✗
Other diseases of intestines (K55-K63)	✗
Disorders of gallbladder, biliary tract and pancreas (K80-K87)	✗
Infections of the skin and subcutaneous tissue (L00-L08)	✗
Dermatitis and eczema (L20-L30)	✗
Urticaria and erythema (L50-L54)	✗
Disorders of skin appendages (L60-L75)	✗
Other disorders of the skin and subcutaneous tissue (L80-L99)	✗
Arthropathies (M00-M25)	✗
Dorsopathies (M40-M54)	✗
Soft tissue disorders (M60-M79)	✗
Osteopathies and chondropathies (M80-M94)	✗
Renal tubulo-interstitial diseases (N10-N16)	✗
Urolithiasis (N20-N23)	✗
Other diseases of urinary system (N30-N39)	✗
Diseases of male genital organs (N40-N51)	✗
Disorders of breast (N60-N64)	✗
Inflammatory diseases of female pelvic organs (N70-N77)	✗
Noninflammatory disorders of female genital tract (N80-N98)	✗

Table S2: Inclusion and exclusion criteria for cluster 1. Female ratio: 50%, mean age of patients: 35, mortality: 0%.

Intestinal infectious diseases (A00-A09)	✗
Other bacterial diseases (A30-A49)	✗
Infections with a predominantly sexual mode of transmission (A50-A64)	✗
Viral infections characterized by skin and mucous membrane lesions (B00-B09)	✗
Other viral diseases (B25-B34)	✗
Malignant neoplasms (C00-C97)	✗
In situ neoplasms (D00-D09)	✗
Benign neoplasms (D10-D36)	✗
Neoplasms of uncertain or unknown behaviour (D37-D48)	✗
Disorders of thyroid gland (E00-E07)	✗
Diabetes mellitus (E10-E14)	✗
Obesity and other hyperalimentation (E65-E68)	✗
Metabolic disorders (E70-E90)	✗
Mental and behavioural disorders due to psychoactive substance use (F10-F19)	✗
Schizophrenia, schizotypal and delusional disorders (F20-F29)	✗
Mood [affective] disorders (F30-F39)	✗
Neurotic, stress-related and somatoform disorders (F40-F48)	✗
Behavioural and emotional disorders with onset usually occurring in childhood and adolescence (F90-F98)	✗
Episodic and paroxysmal disorders (G40-G47)	✗
Nerve, nerve root and plexus disorders (G50-G59)	✗
Disorders of eyelid, lacrimal system and orbit (H00-H06)	✗
Disorders of lens (H25-H28)	✗
Disorders of choroid and retina (H30-H36)	✗
Disorders of ocular muscles, binocular movement, accommodation and refraction (H49-H52)	✗
Diseases of middle ear and mastoid (H65-H75)	✗
Diseases of inner ear (H80-H83)	✗
Other disorders of ear (H90-H95)	✗
Hypertensive diseases (I10-I15)	✗
Ischaemic heart diseases (I20-I25)	✗
Other forms of heart disease (I30-I52)	✗
Cerebrovascular diseases (I60-I69)	✗
Diseases of veins, lymphatic vessels and lymph nodes, not elsewhere classified (I80-I89)	✗
Other and unspecified disorders of the circulatory system (I95-I99)	✓
Acute upper respiratory infections (J00-J06)	✗
Influenza and pneumonia (J09-J18)	✗
Other acute lower respiratory infections (J20-J22)	✗
Other diseases of upper respiratory tract (J30-J39)	✗
Chronic lower respiratory diseases (J40-J47)	✗
Diseases of oral cavity, salivary glands and jaws (K00-K14)	✗
Diseases of oesophagus, stomach and duodenum (K20-K31)	✗
Diseases of appendix (K35-K38)	✗
Hernia (K40-K46)	✗
Noninfective enteritis and colitis (K50-K52)	✗
Other diseases of intestines (K55-K63)	✗
Disorders of gallbladder, biliary tract and pancreas (K80-K87)	✗
Infections of the skin and subcutaneous tissue (L00-L08)	✗
Dermatitis and eczema (L20-L30)	✗
Urticaria and erythema (L50-L54)	✗
Disorders of skin appendages (L60-L75)	✗
Other disorders of the skin and subcutaneous tissue (L80-L99)	✗
Arthropathies (M00-M25)	✗
Dorsopathies (M40-M54)	✗
Soft tissue disorders (M60-M79)	✗
Osteopathies and chondropathies (M80-M94)	✗
Renal tubulo-interstitial diseases (N10-N16)	✗
Urolithiasis (N20-N23)	✗
Other diseases of urinary system (N30-N39)	✗
Diseases of male genital organs (N40-N51)	✗
Disorders of breast (N60-N64)	✗
Inflammatory diseases of female pelvic organs (N70-N77)	✗
Noninflammatory disorders of female genital tract (N80-N98)	✗

Table S3: Inclusion and exclusion criteria for cluster 2. Female ratio: 62%, mean age of patients: 31, mortality: 1%.

Intestinal infectious diseases (A00-A09)	✗
Other bacterial diseases (A30-A49)	✗
Infections with a predominantly sexual mode of transmission (A50-A64)	✗
Viral infections characterized by skin and mucous membrane lesions (B00-B09)	✗
Other viral diseases (B25-B34)	✗
Malignant neoplasms (C00-C97)	✗
In situ neoplasms (D00-D09)	✗
Benign neoplasms (D10-D36)	✗
Neoplasms of uncertain or unknown behaviour (D37-D48)	✗
Disorders of thyroid gland (E00-E07)	✗
Diabetes mellitus (E10-E14)	✗
Obesity and other hyperalimentation (E65-E68)	✓
Metabolic disorders (E70-E90)	✗
Mental and behavioural disorders due to psychoactive substance use (F10-F19)	✗
Schizophrenia, schizotypal and delusional disorders (F20-F29)	✗
Mood [affective] disorders (F30-F39)	✗
Neurotic, stress-related and somatoform disorders (F40-F48)	✗
Behavioural and emotional disorders with onset usually occurring in childhood and adolescence (F90-F98)	✗
Episodic and paroxysmal disorders (G40-G47)	✗
Nerve, nerve root and plexus disorders (G50-G59)	✗
Disorders of eyelid, lacrimal system and orbit (H00-H06)	✗
Disorders of lens (H25-H28)	✗
Disorders of choroid and retina (H30-H36)	✗
Disorders of ocular muscles, binocular movement, accommodation and refraction (H49-H52)	✗
Diseases of middle ear and mastoid (H65-H75)	✗
Diseases of inner ear (H80-H83)	✗
Other disorders of ear (H90-H95)	✗
Hypertensive diseases (I10-I15)	✗
Ischaemic heart diseases (I20-I25)	✗
Other forms of heart disease (I30-I52)	✗
Cerebrovascular diseases (I60-I69)	✗
Diseases of veins, lymphatic vessels and lymph nodes, not elsewhere classified (I80-I89)	✗
Acute upper respiratory infections (J00-J06)	✗
Influenza and pneumonia (J09-J18)	✗
Other acute lower respiratory infections (J20-J22)	✗
Other diseases of upper respiratory tract (J30-J39)	✗
Chronic lower respiratory diseases (J40-J47)	✗
Diseases of oral cavity, salivary glands and jaws (K00-K14)	✗
Diseases of oesophagus, stomach and duodenum (K20-K31)	✗
Diseases of appendix (K35-K38)	✗
Hernia (K40-K46)	✗
Noninfective enteritis and colitis (K50-K52)	✗
Other diseases of intestines (K55-K63)	✗
Disorders of gallbladder, biliary tract and pancreas (K80-K87)	✗
Infections of the skin and subcutaneous tissue (L00-L08)	✗
Dermatitis and eczema (L20-L30)	✗
Urticaria and erythema (L50-L54)	✗
Disorders of skin appendages (L60-L75)	✗
Other disorders of the skin and subcutaneous tissue (L80-L99)	✗
Arthropathies (M00-M25)	✗
Dorsopathies (M40-M54)	✗
Soft tissue disorders (M60-M79)	✗
Osteopathies and chondropathies (M80-M94)	✗
Renal tubulo-interstitial diseases (N10-N16)	✗
Urolithiasis (N20-N23)	✗
Other diseases of urinary system (N30-N39)	✗
Diseases of male genital organs (N40-N51)	✗
Disorders of breast (N60-N64)	✗
Inflammatory diseases of female pelvic organs (N70-N77)	✗
Noninflammatory disorders of female genital tract (N80-N98)	✗

Table S4: Inclusion and exclusion criteria for cluster 3. Female ratio: 71%, mean age of patients: 33, mortality: 0%.

Intestinal infectious diseases (A00-A09)	✗
Other bacterial diseases (A30-A49)	✗
Infections with a predominantly sexual mode of transmission (A50-A64)	✗
Viral infections characterized by skin and mucous membrane lesions (B00-B09)	✗
Other viral diseases (B25-B34)	✗
Malignant neoplasms (C00-C97)	✗
In situ neoplasms (D00-D09)	✗
Benign neoplasms (D10-D36)	✗
Neoplasms of uncertain or unknown behaviour (D37-D48)	✗
Disorders of thyroid gland (E00-E07)	✗
Diabetes mellitus (E10-E14)	✗
Metabolic disorders (E70-E90)	✗
Mental and behavioural disorders due to psychoactive substance use (F10-F19)	✗
Schizophrenia, schizotypal and delusional disorders (F20-F29)	✗
Mood [affective] disorders (F30-F39)	✗
Neurotic, stress-related and somatoform disorders (F40-F48)	✗
Behavioural and emotional disorders with onset usually occurring in childhood and adolescence (F90-F98)	✓
Episodic and paroxysmal disorders (G40-G47)	✗
Nerve, nerve root and plexus disorders (G50-G59)	✗
Disorders of eyelid, lacrimal system and orbit (H00-H06)	✗
Disorders of lens (H25-H28)	✗
Disorders of choroid and retina (H30-H36)	✗
Disorders of ocular muscles, binocular movement, accommodation and refraction (H49-H52)	✗
Diseases of middle ear and mastoid (H65-H75)	✗
Diseases of inner ear (H80-H83)	✗
Other disorders of ear (H90-H95)	✗
Hypertensive diseases (I10-I15)	✗
Ischaemic heart diseases (I20-I25)	✗
Other forms of heart disease (I30-I52)	✗
Cerebrovascular diseases (I60-I69)	✗
Diseases of veins, lymphatic vessels and lymph nodes, not elsewhere classified (I80-I89)	✗
Acute upper respiratory infections (J00-J06)	✗
Influenza and pneumonia (J09-J18)	✗
Other acute lower respiratory infections (J20-J22)	✗
Other diseases of upper respiratory tract (J30-J39)	✗
Chronic lower respiratory diseases (J40-J47)	✗
Diseases of oral cavity, salivary glands and jaws (K00-K14)	✗
Diseases of oesophagus, stomach and duodenum (K20-K31)	✗
Diseases of appendix (K35-K38)	✗
Hernia (K40-K46)	✗
Noninfective enteritis and colitis (K50-K52)	✗
Other diseases of intestines (K55-K63)	✗
Disorders of gallbladder, biliary tract and pancreas (K80-K87)	✗
Infections of the skin and subcutaneous tissue (L00-L08)	✗
Dermatitis and eczema (L20-L30)	✗
Urticaria and erythema (L50-L54)	✗
Disorders of skin appendages (L60-L75)	✗
Other disorders of the skin and subcutaneous tissue (L80-L99)	✗
Arthropathies (M00-M25)	✗
Dorsopathies (M40-M54)	✗
Soft tissue disorders (M60-M79)	✗
Osteopathies and chondropathies (M80-M94)	✗
Renal tubulo-interstitial diseases (N10-N16)	✗
Urolithiasis (N20-N23)	✗
Other diseases of urinary system (N30-N39)	✗
Diseases of male genital organs (N40-N51)	✗
Disorders of breast (N60-N64)	✗
Inflammatory diseases of female pelvic organs (N70-N77)	✗
Noninflammatory disorders of female genital tract (N80-N98)	✗

Table S5: Inclusion and exclusion criteria for cluster 4. Female ratio: 29%, mean age of patients: 14, mortality: 0%.

Intestinal infectious diseases (A00-A09)	✗
Other bacterial diseases (A30-A49)	✗
Infections with a predominantly sexual mode of transmission (A50-A64)	✗
Viral infections characterized by skin and mucous membrane lesions (B00-B09)	✗
Other viral diseases (B25-B34)	✗
Malignant neoplasms (C00-C97)	✗
In situ neoplasms (D00-D09)	✗
Benign neoplasms (D10-D36)	✗
Neoplasms of uncertain or unknown behaviour (D37-D48)	✗
Disorders of thyroid gland (E00-E07)	✗
Diabetes mellitus (E10-E14)	✗
Metabolic disorders (E70-E90)	✗
Mental and behavioural disorders due to psychoactive substance use (F10-F19)	✗
Schizophrenia, schizotypal and delusional disorders (F20-F29)	✗
Mood [affective] disorders (F30-F39)	✗
Neurotic, stress-related and somatoform disorders (F40-F48)	✗
Episodic and paroxysmal disorders (G40-G47)	✗
Nerve, nerve root and plexus disorders (G50-G59)	✗
Disorders of eyelid, lacrimal system and orbit (H00-H06)	✗
Disorders of lens (H25-H28)	✗
Disorders of choroid and retina (H30-H36)	✗
Disorders of ocular muscles, binocular movement, accommodation and refraction (H49-H52)	✗
Diseases of middle ear and mastoid (H65-H75)	✗
Diseases of inner ear (H80-H83)	✗
Other disorders of ear (H90-H95)	✗
Hypertensive diseases (I10-I15)	✗
Ischaemic heart diseases (I20-I25)	✗
Other forms of heart disease (I30-I52)	✗
Cerebrovascular diseases (I60-I69)	✗
Diseases of veins, lymphatic vessels and lymph nodes, not elsewhere classified (I80-I89)	✗
Acute upper respiratory infections (J00-J06)	✗
Influenza and pneumonia (J09-J18)	✗
Other acute lower respiratory infections (J20-J22)	✗
Other diseases of upper respiratory tract (J30-J39)	✗
Chronic lower respiratory diseases (J40-J47)	✗
Diseases of oral cavity, salivary glands and jaws (K00-K14)	✗
Diseases of oesophagus, stomach and duodenum (K20-K31)	✗
Diseases of appendix (K35-K38)	✗
Hernia (K40-K46)	✗
Noninfective enteritis and colitis (K50-K52)	✗
Other diseases of intestines (K55-K63)	✗
Disorders of gallbladder, biliary tract and pancreas (K80-K87)	✗
Infections of the skin and subcutaneous tissue (L00-L08)	✗
Dermatitis and eczema (L20-L30)	✗
Urticaria and erythema (L50-L54)	✓
Disorders of skin appendages (L60-L75)	✗
Other disorders of the skin and subcutaneous tissue (L80-L99)	✗
Arthropathies (M00-M25)	✗
Dorsopathies (M40-M54)	✗
Soft tissue disorders (M60-M79)	✗
Osteopathies and chondropathies (M80-M94)	✗
Renal tubulo-interstitial diseases (N10-N16)	✗
Urolithiasis (N20-N23)	✗
Other diseases of urinary system (N30-N39)	✗
Diseases of male genital organs (N40-N51)	✗
Disorders of breast (N60-N64)	✗
Inflammatory diseases of female pelvic organs (N70-N77)	✗
Noninflammatory disorders of female genital tract (N80-N98)	✗

Table S6: Inclusion and exclusion criteria for cluster 5. Female ratio: 54%, mean age of patients: 27, mortality: 0%.

Intestinal infectious diseases (A00-A09)	✗
Other bacterial diseases (A30-A49)	✗
Infections with a predominantly sexual mode of transmission (A50-A64)	✗
Viral infections characterized by skin and mucous membrane lesions (B00-B09)	✗
Other viral diseases (B25-B34)	✗
Malignant neoplasms (C00-C97)	✗
In situ neoplasms (D00-D09)	✗
Benign neoplasms (D10-D36)	✗
Neoplasms of uncertain or unknown behaviour (D37-D48)	✗
Disorders of thyroid gland (E00-E07)	✗
Diabetes mellitus (E10-E14)	✓
Metabolic disorders (E70-E90)	✗
Mental and behavioural disorders due to psychoactive substance use (F10-F19)	✗
Schizophrenia, schizotypal and delusional disorders (F20-F29)	✗
Mood [affective] disorders (F30-F39)	✗
Neurotic, stress-related and somatoform disorders (F40-F48)	✗
Episodic and paroxysmal disorders (G40-G47)	✗
Nerve, nerve root and plexus disorders (G50-G59)	✗
Disorders of eyelid, lacrimal system and orbit (H00-H06)	✗
Disorders of lens (H25-H28)	✗
Disorders of choroid and retina (H30-H36)	✗
Disorders of ocular muscles, binocular movement, accommodation and refraction (H49-H52)	✗
Diseases of middle ear and mastoid (H65-H75)	✗
Diseases of inner ear (H80-H83)	✗
Other disorders of ear (H90-H95)	✗
Hypertensive diseases (I10-I15)	✗
Ischaemic heart diseases (I20-I25)	✗
Other forms of heart disease (I30-I52)	✗
Cerebrovascular diseases (I60-I69)	✗
Diseases of veins, lymphatic vessels and lymph nodes, not elsewhere classified (I80-I89)	✗
Acute upper respiratory infections (J00-J06)	✗
Influenza and pneumonia (J09-J18)	✗
Other acute lower respiratory infections (J20-J22)	✗
Other diseases of upper respiratory tract (J30-J39)	✗
Chronic lower respiratory diseases (J40-J47)	✗
Diseases of oral cavity, salivary glands and jaws (K00-K14)	✗
Diseases of oesophagus, stomach and duodenum (K20-K31)	✗
Diseases of appendix (K35-K38)	✗
Hernia (K40-K46)	✗
Noninfective enteritis and colitis (K50-K52)	✗
Other diseases of intestines (K55-K63)	✗
Disorders of gallbladder, biliary tract and pancreas (K80-K87)	✗
Infections of the skin and subcutaneous tissue (L00-L08)	✗
Dermatitis and eczema (L20-L30)	✗
Disorders of skin appendages (L60-L75)	✗
Other disorders of the skin and subcutaneous tissue (L80-L99)	✗
Arthropathies (M00-M25)	✗
Dorsopathies (M40-M54)	✗
Soft tissue disorders (M60-M79)	✗
Osteopathies and chondropathies (M80-M94)	✗
Renal tubulo-interstitial diseases (N10-N16)	✗
Urolithiasis (N20-N23)	✗
Other diseases of urinary system (N30-N39)	✗
Diseases of male genital organs (N40-N51)	✗
Disorders of breast (N60-N64)	✗
Inflammatory diseases of female pelvic organs (N70-N77)	✗
Noninflammatory disorders of female genital tract (N80-N98)	✗

Table S7: Inclusion and exclusion criteria for cluster 6. Female ratio: 42%, mean age of patients: 47, mortality: 0%.

Intestinal infectious diseases (A00-A09)	✗
Other bacterial diseases (A30-A49)	✗
Infections with a predominantly sexual mode of transmission (A50-A64)	✗
Viral infections characterized by skin and mucous membrane lesions (B00-B09)	✗
Other viral diseases (B25-B34)	✗
Malignant neoplasms (C00-C97)	✗
In situ neoplasms (D00-D09)	✓
Benign neoplasms (D10-D36)	✗
Neoplasms of uncertain or unknown behaviour (D37-D48)	✗
Disorders of thyroid gland (E00-E07)	✗
Metabolic disorders (E70-E90)	✗
Mental and behavioural disorders due to psychoactive substance use (F10-F19)	✗
Schizophrenia, schizotypal and delusional disorders (F20-F29)	✗
Mood [affective] disorders (F30-F39)	✗
Neurotic, stress-related and somatoform disorders (F40-F48)	✗
Episodic and paroxysmal disorders (G40-G47)	✗
Nerve, nerve root and plexus disorders (G50-G59)	✗
Disorders of eyelid, lacrimal system and orbit (H00-H06)	✗
Disorders of lens (H25-H28)	✗
Disorders of choroid and retina (H30-H36)	✗
Disorders of ocular muscles, binocular movement, accommodation and refraction (H49-H52)	✗
Diseases of middle ear and mastoid (H65-H75)	✗
Diseases of inner ear (H80-H83)	✗
Other disorders of ear (H90-H95)	✗
Hypertensive diseases (I10-I15)	✗
Ischaemic heart diseases (I20-I25)	✗
Other forms of heart disease (I30-I52)	✗
Cerebrovascular diseases (I60-I69)	✗
Diseases of veins, lymphatic vessels and lymph nodes, not elsewhere classified (I80-I89)	✗
Acute upper respiratory infections (J00-J06)	✗
Influenza and pneumonia (J09-J18)	✗
Other acute lower respiratory infections (J20-J22)	✗
Other diseases of upper respiratory tract (J30-J39)	✗
Chronic lower respiratory diseases (J40-J47)	✗
Diseases of oral cavity, salivary glands and jaws (K00-K14)	✗
Diseases of oesophagus, stomach and duodenum (K20-K31)	✗
Diseases of appendix (K35-K38)	✗
Hernia (K40-K46)	✗
Noninfective enteritis and colitis (K50-K52)	✗
Other diseases of intestines (K55-K63)	✗
Disorders of gallbladder, biliary tract and pancreas (K80-K87)	✗
Infections of the skin and subcutaneous tissue (L00-L08)	✗
Dermatitis and eczema (L20-L30)	✗
Disorders of skin appendages (L60-L75)	✗
Other disorders of the skin and subcutaneous tissue (L80-L99)	✗
Arthropathies (M00-M25)	✗
Dorsopathies (M40-M54)	✗
Soft tissue disorders (M60-M79)	✗
Osteopathies and chondropathies (M80-M94)	✗
Renal tubulo-interstitial diseases (N10-N16)	✗
Urolithiasis (N20-N23)	✗
Other diseases of urinary system (N30-N39)	✗
Diseases of male genital organs (N40-N51)	✗
Disorders of breast (N60-N64)	✗
Inflammatory diseases of female pelvic organs (N70-N77)	✗
Noninflammatory disorders of female genital tract (N80-N98)	✗

Table S8: Inclusion and exclusion criteria for cluster 7. Female ratio: 97%, mean age of patients: 38, mortality: 0%.

Intestinal infectious diseases (A00-A09)	✗
Other bacterial diseases (A30-A49)	✗
Infections with a predominantly sexual mode of transmission (A50-A64)	✗
Viral infections characterized by skin and mucous membrane lesions (B00-B09)	✗
Other viral diseases (B25-B34)	✗
Malignant neoplasms (C00-C97)	✗
Benign neoplasms (D10-D36)	✗
Neoplasms of uncertain or unknown behaviour (D37-D48)	✗
Disorders of thyroid gland (E00-E07)	✗
Metabolic disorders (E70-E90)	✗
Mental and behavioural disorders due to psychoactive substance use (F10-F19)	✗
Schizophrenia, schizotypal and delusional disorders (F20-F29)	✗
Mood [affective] disorders (F30-F39)	✗
Neurotic, stress-related and somatoform disorders (F40-F48)	✗
Episodic and paroxysmal disorders (G40-G47)	✗
Nerve, nerve root and plexus disorders (G50-G59)	✗
Disorders of eyelid, lacrimal system and orbit (H00-H06)	✗
Disorders of lens (H25-H28)	✗
Disorders of choroid and retina (H30-H36)	✗
Disorders of ocular muscles, binocular movement, accommodation and refraction (H49-H52)	✗
Diseases of middle ear and mastoid (H65-H75)	✗
Diseases of inner ear (H80-H83)	✗
Other disorders of ear (H90-H95)	✗
Hypertensive diseases (I10-I15)	✗
Ischaemic heart diseases (I20-I25)	✗
Other forms of heart disease (I30-I52)	✗
Cerebrovascular diseases (I60-I69)	✗
Diseases of veins, lymphatic vessels and lymph nodes, not elsewhere classified (I80-I89)	✗
Acute upper respiratory infections (J00-J06)	✗
Influenza and pneumonia (J09-J18)	✗
Other acute lower respiratory infections (J20-J22)	✗
Other diseases of upper respiratory tract (J30-J39)	✗
Chronic lower respiratory diseases (J40-J47)	✗
Diseases of oral cavity, salivary glands and jaws (K00-K14)	✗
Diseases of oesophagus, stomach and duodenum (K20-K31)	✗
Diseases of appendix (K35-K38)	✗
Hernia (K40-K46)	✗
Noninfective enteritis and colitis (K50-K52)	✗
Other diseases of intestines (K55-K63)	✗
Disorders of gallbladder, biliary tract and pancreas (K80-K87)	✗
Infections of the skin and subcutaneous tissue (L00-L08)	✗
Dermatitis and eczema (L20-L30)	✓
Disorders of skin appendages (L60-L75)	✗
Other disorders of the skin and subcutaneous tissue (L80-L99)	✗
Arthropathies (M00-M25)	✗
Dorsopathies (M40-M54)	✗
Soft tissue disorders (M60-M79)	✗
Osteopathies and chondropathies (M80-M94)	✗
Renal tubulo-interstitial diseases (N10-N16)	✗
Urolithiasis (N20-N23)	✗
Other diseases of urinary system (N30-N39)	✗
Diseases of male genital organs (N40-N51)	✗
Disorders of breast (N60-N64)	✗
Inflammatory diseases of female pelvic organs (N70-N77)	✗
Noninflammatory disorders of female genital tract (N80-N98)	✗

Table S9: Inclusion and exclusion criteria for cluster 8. Female ratio: 57%, mean age of patients: 27, mortality: 0%.

Intestinal infectious diseases (A00-A09)	✗
Other bacterial diseases (A30-A49)	✗
Infections with a predominantly sexual mode of transmission (A50-A64)	✗
Viral infections characterized by skin and mucous membrane lesions (B00-B09)	✗
Other viral diseases (B25-B34)	✗
Malignant neoplasms (C00-C97)	✗
Benign neoplasms (D10-D36)	✗
Neoplasms of uncertain or unknown behaviour (D37-D48)	✗
Disorders of thyroid gland (E00-E07)	✗
Metabolic disorders (E70-E90)	✗
Mental and behavioural disorders due to psychoactive substance use (F10-F19)	✗
Schizophrenia, schizotypal and delusional disorders (F20-F29)	✗
Mood [affective] disorders (F30-F39)	✗
Neurotic, stress-related and somatoform disorders (F40-F48)	✗
Episodic and paroxysmal disorders (G40-G47)	✗
Nerve, nerve root and plexus disorders (G50-G59)	✗
Disorders of eyelid, lacrimal system and orbit (H00-H06)	✗
Disorders of lens (H25-H28)	✗
Disorders of choroid and retina (H30-H36)	✗
Disorders of ocular muscles, binocular movement, accommodation and refraction (H49-H52)	✗
Diseases of middle ear and mastoid (H65-H75)	✗
Diseases of inner ear (H80-H83)	✗
Other disorders of ear (H90-H95)	✗
Hypertensive diseases (I10-I15)	✗
Ischaemic heart diseases (I20-I25)	✓
Other forms of heart disease (I30-I52)	✗
Cerebrovascular diseases (I60-I69)	✗
Diseases of veins, lymphatic vessels and lymph nodes, not elsewhere classified (I80-I89)	✗
Acute upper respiratory infections (J00-J06)	✗
Influenza and pneumonia (J09-J18)	✗
Other acute lower respiratory infections (J20-J22)	✗
Other diseases of upper respiratory tract (J30-J39)	✗
Chronic lower respiratory diseases (J40-J47)	✗
Diseases of oral cavity, salivary glands and jaws (K00-K14)	✗
Diseases of oesophagus, stomach and duodenum (K20-K31)	✗
Diseases of appendix (K35-K38)	✗
Hernia (K40-K46)	✗
Noninfective enteritis and colitis (K50-K52)	✗
Other diseases of intestines (K55-K63)	✗
Disorders of gallbladder, biliary tract and pancreas (K80-K87)	✗
Infections of the skin and subcutaneous tissue (L00-L08)	✗
Disorders of skin appendages (L60-L75)	✗
Other disorders of the skin and subcutaneous tissue (L80-L99)	✗
Arthropathies (M00-M25)	✗
Dorsopathies (M40-M54)	✗
Soft tissue disorders (M60-M79)	✗
Osteopathies and chondropathies (M80-M94)	✗
Renal tubulo-interstitial diseases (N10-N16)	✗
Urolithiasis (N20-N23)	✗
Other diseases of urinary system (N30-N39)	✗
Diseases of male genital organs (N40-N51)	✗
Disorders of breast (N60-N64)	✗
Inflammatory diseases of female pelvic organs (N70-N77)	✗
Noninflammatory disorders of female genital tract (N80-N98)	✗

Table S10: Inclusion and exclusion criteria for cluster 9. Female ratio: 32%, mean age of patients: 63, mortality: 2%.

Intestinal infectious diseases (A00-A09)	✗
Other bacterial diseases (A30-A49)	✗
Infections with a predominantly sexual mode of transmission (A50-A64)	✗
Viral infections characterized by skin and mucous membrane lesions (B00-B09)	✗
Other viral diseases (B25-B34)	✗
Malignant neoplasms (C00-C97)	✗
Benign neoplasms (D10-D36)	✗
Neoplasms of uncertain or unknown behaviour (D37-D48)	✗
Disorders of thyroid gland (E00-E07)	✗
Metabolic disorders (E70-E90)	✗
Mental and behavioural disorders due to psychoactive substance use (F10-F19)	✗
Schizophrenia, schizotypal and delusional disorders (F20-F29)	✗
Mood [affective] disorders (F30-F39)	✗
Neurotic, stress-related and somatoform disorders (F40-F48)	✗
Episodic and paroxysmal disorders (G40-G47)	✗
Nerve, nerve root and plexus disorders (G50-G59)	✗
Disorders of eyelid, lacrimal system and orbit (H00-H06)	✗
Disorders of lens (H25-H28)	✗
Disorders of choroid and retina (H30-H36)	✗
Disorders of ocular muscles, binocular movement, accommodation and refraction (H49-H52)	✗
Diseases of middle ear and mastoid (H65-H75)	✗
Diseases of inner ear (H80-H83)	✗
Other disorders of ear (H90-H95)	✗
Hypertensive diseases (I10-I15)	✗
Other forms of heart disease (I30-I52)	✗
Cerebrovascular diseases (I60-I69)	✗
Diseases of veins, lymphatic vessels and lymph nodes, not elsewhere classified (I80-I89)	✗
Acute upper respiratory infections (J00-J06)	✗
Influenza and pneumonia (J09-J18)	✗
Other acute lower respiratory infections (J20-J22)	✗
Other diseases of upper respiratory tract (J30-J39)	✗
Chronic lower respiratory diseases (J40-J47)	✗
Diseases of oral cavity, salivary glands and jaws (K00-K14)	✗
Diseases of oesophagus, stomach and duodenum (K20-K31)	✗
Diseases of appendix (K35-K38)	✗
Hernia (K40-K46)	✗
Noninfective enteritis and colitis (K50-K52)	✗
Other diseases of intestines (K55-K63)	✗
Disorders of gallbladder, biliary tract and pancreas (K80-K87)	✗
Infections of the skin and subcutaneous tissue (L00-L08)	✗
Disorders of skin appendages (L60-L75)	✗
Other disorders of the skin and subcutaneous tissue (L80-L99)	✓
Arthropathies (M00-M25)	✗
Dorsopathies (M40-M54)	✗
Soft tissue disorders (M60-M79)	✗
Osteopathies and chondropathies (M80-M94)	✗
Renal tubulo-interstitial diseases (N10-N16)	✗
Urolithiasis (N20-N23)	✗
Other diseases of urinary system (N30-N39)	✗
Diseases of male genital organs (N40-N51)	✗
Disorders of breast (N60-N64)	✗
Inflammatory diseases of female pelvic organs (N70-N77)	✗
Noninflammatory disorders of female genital tract (N80-N98)	✗

Table S11: Inclusion and exclusion criteria for cluster 10. Female ratio: 55%, mean age of patients: 39, mortality: 0%.

Intestinal infectious diseases (A00-A09)	✗
Other bacterial diseases (A30-A49)	✗
Infections with a predominantly sexual mode of transmission (A50-A64)	✗
Viral infections characterized by skin and mucous membrane lesions (B00-B09)	✗
Other viral diseases (B25-B34)	✗
Malignant neoplasms (C00-C97)	✗
Benign neoplasms (D10-D36)	✗
Neoplasms of uncertain or unknown behaviour (D37-D48)	✗
Disorders of thyroid gland (E00-E07)	✗
Metabolic disorders (E70-E90)	✗
Mental and behavioural disorders due to psychoactive substance use (F10-F19)	✗
Schizophrenia, schizotypal and delusional disorders (F20-F29)	✗
Mood [affective] disorders (F30-F39)	✗
Neurotic, stress-related and somatoform disorders (F40-F48)	✗
Episodic and paroxysmal disorders (G40-G47)	✗
Nerve, nerve root and plexus disorders (G50-G59)	✗
Disorders of eyelid, lacrimal system and orbit (H00-H06)	✗
Disorders of lens (H25-H28)	✗
Disorders of choroid and retina (H30-H36)	✗
Disorders of ocular muscles, binocular movement, accommodation and refraction (H49-H52)	✗
Diseases of middle ear and mastoid (H65-H75)	✗
Diseases of inner ear (H80-H83)	✗
Other disorders of ear (H90-H95)	✗
Hypertensive diseases (I10-I15)	✗
Other forms of heart disease (I30-I52)	✗
Cerebrovascular diseases (I60-I69)	✓
Diseases of veins, lymphatic vessels and lymph nodes, not elsewhere classified (I80-I89)	✗
Acute upper respiratory infections (J00-J06)	✗
Influenza and pneumonia (J09-J18)	✗
Other acute lower respiratory infections (J20-J22)	✗
Other diseases of upper respiratory tract (J30-J39)	✗
Chronic lower respiratory diseases (J40-J47)	✗
Diseases of oral cavity, salivary glands and jaws (K00-K14)	✗
Diseases of oesophagus, stomach and duodenum (K20-K31)	✗
Diseases of appendix (K35-K38)	✗
Hernia (K40-K46)	✗
Noninfective enteritis and colitis (K50-K52)	✗
Other diseases of intestines (K55-K63)	✗
Disorders of gallbladder, biliary tract and pancreas (K80-K87)	✗
Infections of the skin and subcutaneous tissue (L00-L08)	✗
Disorders of skin appendages (L60-L75)	✗
Arthropathies (M00-M25)	✗
Dorsopathies (M40-M54)	✗
Soft tissue disorders (M60-M79)	✗
Osteopathies and chondropathies (M80-M94)	✗
Renal tubulo-interstitial diseases (N10-N16)	✗
Urolithiasis (N20-N23)	✗
Other diseases of urinary system (N30-N39)	✗
Diseases of male genital organs (N40-N51)	✗
Disorders of breast (N60-N64)	✗
Inflammatory diseases of female pelvic organs (N70-N77)	✗
Noninflammatory disorders of female genital tract (N80-N98)	✗

Table S12: Inclusion and exclusion criteria for cluster 11. Female ratio: 47%, mean age of patients: 61, mortality: 4%.

Intestinal infectious diseases (A00-A09)	✗
Other bacterial diseases (A30-A49)	✗
Infections with a predominantly sexual mode of transmission (A50-A64)	✗
Viral infections characterized by skin and mucous membrane lesions (B00-B09)	✗
Other viral diseases (B25-B34)	✓
Malignant neoplasms (C00-C97)	✗
Benign neoplasms (D10-D36)	✗
Neoplasms of uncertain or unknown behaviour (D37-D48)	✗
Disorders of thyroid gland (E00-E07)	✗
Metabolic disorders (E70-E90)	✗
Mental and behavioural disorders due to psychoactive substance use (F10-F19)	✗
Schizophrenia, schizotypal and delusional disorders (F20-F29)	✗
Mood [affective] disorders (F30-F39)	✗
Neurotic, stress-related and somatoform disorders (F40-F48)	✗
Episodic and paroxysmal disorders (G40-G47)	✗
Nerve, nerve root and plexus disorders (G50-G59)	✗
Disorders of eyelid, lacrimal system and orbit (H00-H06)	✗
Disorders of lens (H25-H28)	✗
Disorders of choroid and retina (H30-H36)	✗
Disorders of ocular muscles, binocular movement, accommodation and refraction (H49-H52)	✗
Diseases of middle ear and mastoid (H65-H75)	✗
Diseases of inner ear (H80-H83)	✗
Other disorders of ear (H90-H95)	✗
Hypertensive diseases (I10-I15)	✗
Other forms of heart disease (I30-I52)	✗
Diseases of veins, lymphatic vessels and lymph nodes, not elsewhere classified (I80-I89)	✗
Acute upper respiratory infections (J00-J06)	✗
Influenza and pneumonia (J09-J18)	✗
Other acute lower respiratory infections (J20-J22)	✗
Other diseases of upper respiratory tract (J30-J39)	✗
Chronic lower respiratory diseases (J40-J47)	✗
Diseases of oral cavity, salivary glands and jaws (K00-K14)	✗
Diseases of oesophagus, stomach and duodenum (K20-K31)	✗
Diseases of appendix (K35-K38)	✗
Hernia (K40-K46)	✗
Noninfective enteritis and colitis (K50-K52)	✗
Other diseases of intestines (K55-K63)	✗
Disorders of gallbladder, biliary tract and pancreas (K80-K87)	✗
Infections of the skin and subcutaneous tissue (L00-L08)	✗
Disorders of skin appendages (L60-L75)	✗
Arthropathies (M00-M25)	✗
Dorsopathies (M40-M54)	✗
Soft tissue disorders (M60-M79)	✗
Osteopathies and chondropathies (M80-M94)	✗
Renal tubulo-interstitial diseases (N10-N16)	✗
Urolithiasis (N20-N23)	✗
Other diseases of urinary system (N30-N39)	✗
Diseases of male genital organs (N40-N51)	✗
Disorders of breast (N60-N64)	✗
Inflammatory diseases of female pelvic organs (N70-N77)	✗
Noninflammatory disorders of female genital tract (N80-N98)	✗

Table S13: Inclusion and exclusion criteria for cluster 12. Female ratio: 47%, mean age of patients: 18, mortality: 0%.

Intestinal infectious diseases (A00-A09)	✗
Other bacterial diseases (A30-A49)	✗
Infections with a predominantly sexual mode of transmission (A50-A64)	✗
Viral infections characterized by skin and mucous membrane lesions (B00-B09)	✗
Malignant neoplasms (C00-C97)	✗
Benign neoplasms (D10-D36)	✗
Neoplasms of uncertain or unknown behaviour (D37-D48)	✗
Disorders of thyroid gland (E00-E07)	✗
Metabolic disorders (E70-E90)	✗
Mental and behavioural disorders due to psychoactive substance use (F10-F19)	✗
Schizophrenia, schizotypal and delusional disorders (F20-F29)	✗
Mood [affective] disorders (F30-F39)	✗
Neurotic, stress-related and somatoform disorders (F40-F48)	✗
Episodic and paroxysmal disorders (G40-G47)	✗
Nerve, nerve root and plexus disorders (G50-G59)	✗
Disorders of eyelid, lacrimal system and orbit (H00-H06)	✗
Disorders of lens (H25-H28)	✗
Disorders of choroid and retina (H30-H36)	✗
Disorders of ocular muscles, binocular movement, accommodation and refraction (H49-H52)	✗
Diseases of middle ear and mastoid (H65-H75)	✗
Diseases of inner ear (H80-H83)	✓
Other disorders of ear (H90-H95)	✗
Hypertensive diseases (I10-I15)	✗
Other forms of heart disease (I30-I52)	✗
Diseases of veins, lymphatic vessels and lymph nodes, not elsewhere classified (I80-I89)	✗
Acute upper respiratory infections (J00-J06)	✗
Influenza and pneumonia (J09-J18)	✗
Other acute lower respiratory infections (J20-J22)	✗
Other diseases of upper respiratory tract (J30-J39)	✗
Chronic lower respiratory diseases (J40-J47)	✗
Diseases of oral cavity, salivary glands and jaws (K00-K14)	✗
Diseases of oesophagus, stomach and duodenum (K20-K31)	✗
Diseases of appendix (K35-K38)	✗
Hernia (K40-K46)	✗
Noninfective enteritis and colitis (K50-K52)	✗
Other diseases of intestines (K55-K63)	✗
Disorders of gallbladder, biliary tract and pancreas (K80-K87)	✗
Infections of the skin and subcutaneous tissue (L00-L08)	✗
Disorders of skin appendages (L60-L75)	✗
Arthropathies (M00-M25)	✗
Dorsopathies (M40-M54)	✗
Soft tissue disorders (M60-M79)	✗
Osteopathies and chondropathies (M80-M94)	✗
Renal tubulo-interstitial diseases (N10-N16)	✗
Urolithiasis (N20-N23)	✗
Other diseases of urinary system (N30-N39)	✗
Diseases of male genital organs (N40-N51)	✗
Disorders of breast (N60-N64)	✗
Inflammatory diseases of female pelvic organs (N70-N77)	✗
Noninflammatory disorders of female genital tract (N80-N98)	✗

Table S14: Inclusion and exclusion criteria for cluster 13. Female ratio: 51%, mean age of patients: 45, mortality: 0%.

Intestinal infectious diseases (A00-A09)	✗
Other bacterial diseases (A30-A49)	✓
Infections with a predominantly sexual mode of transmission (A50-A64)	✗
Viral infections characterized by skin and mucous membrane lesions (B00-B09)	✗
Malignant neoplasms (C00-C97)	✗
Benign neoplasms (D10-D36)	✗
Neoplasms of uncertain or unknown behaviour (D37-D48)	✗
Disorders of thyroid gland (E00-E07)	✗
Metabolic disorders (E70-E90)	✗
Mental and behavioural disorders due to psychoactive substance use (F10-F19)	✗
Schizophrenia, schizotypal and delusional disorders (F20-F29)	✗
Mood [affective] disorders (F30-F39)	✗
Neurotic, stress-related and somatoform disorders (F40-F48)	✗
Episodic and paroxysmal disorders (G40-G47)	✗
Nerve, nerve root and plexus disorders (G50-G59)	✗
Disorders of eyelid, lacrimal system and orbit (H00-H06)	✗
Disorders of lens (H25-H28)	✗
Disorders of choroid and retina (H30-H36)	✗
Disorders of ocular muscles, binocular movement, accommodation and refraction (H49-H52)	✗
Diseases of middle ear and mastoid (H65-H75)	✗
Other disorders of ear (H90-H95)	✗
Hypertensive diseases (I10-I15)	✗
Other forms of heart disease (I30-I52)	✗
Diseases of veins, lymphatic vessels and lymph nodes, not elsewhere classified (I80-I89)	✗
Acute upper respiratory infections (J00-J06)	✗
Influenza and pneumonia (J09-J18)	✗
Other acute lower respiratory infections (J20-J22)	✗
Other diseases of upper respiratory tract (J30-J39)	✗
Chronic lower respiratory diseases (J40-J47)	✗
Diseases of oral cavity, salivary glands and jaws (K00-K14)	✗
Diseases of oesophagus, stomach and duodenum (K20-K31)	✗
Diseases of appendix (K35-K38)	✗
Hernia (K40-K46)	✗
Noninfective enteritis and colitis (K50-K52)	✗
Other diseases of intestines (K55-K63)	✗
Disorders of gallbladder, biliary tract and pancreas (K80-K87)	✗
Infections of the skin and subcutaneous tissue (L00-L08)	✗
Disorders of skin appendages (L60-L75)	✗
Arthropathies (M00-M25)	✗
Dorsopathies (M40-M54)	✗
Soft tissue disorders (M60-M79)	✗
Osteopathies and chondropathies (M80-M94)	✗
Renal tubulo-interstitial diseases (N10-N16)	✗
Urolithiasis (N20-N23)	✗
Other diseases of urinary system (N30-N39)	✗
Diseases of male genital organs (N40-N51)	✗
Disorders of breast (N60-N64)	✗
Inflammatory diseases of female pelvic organs (N70-N77)	✗
Noninflammatory disorders of female genital tract (N80-N98)	✗

Table S15: Inclusion and exclusion criteria for cluster 14. Female ratio: 45%, mean age of patients: 36, mortality: 1%.

Intestinal infectious diseases (A00-A09)	✗
Infections with a predominantly sexual mode of transmission (A50-A64)	✗
Viral infections characterized by skin and mucous membrane lesions (B00-B09)	✗
Malignant neoplasms (C00-C97)	✗
Benign neoplasms (D10-D36)	✗
Neoplasms of uncertain or unknown behaviour (D37-D48)	✗
Disorders of thyroid gland (E00-E07)	✗
Metabolic disorders (E70-E90)	✗
Mental and behavioural disorders due to psychoactive substance use (F10-F19)	✗
Schizophrenia, schizotypal and delusional disorders (F20-F29)	✗
Mood [affective] disorders (F30-F39)	✗
Neurotic, stress-related and somatoform disorders (F40-F48)	✗
Episodic and paroxysmal disorders (G40-G47)	✗
Nerve, nerve root and plexus disorders (G50-G59)	✗
Disorders of eyelid, lacrimal system and orbit (H00-H06)	✗
Disorders of lens (H25-H28)	✗
Disorders of choroid and retina (H30-H36)	✓
Disorders of ocular muscles, binocular movement, accommodation and refraction (H49-H52)	✗
Diseases of middle ear and mastoid (H65-H75)	✗
Other disorders of ear (H90-H95)	✗
Hypertensive diseases (I10-I15)	✗
Other forms of heart disease (I30-I52)	✗
Diseases of veins, lymphatic vessels and lymph nodes, not elsewhere classified (I80-I89)	✗
Acute upper respiratory infections (J00-J06)	✗
Influenza and pneumonia (J09-J18)	✗
Other acute lower respiratory infections (J20-J22)	✗
Other diseases of upper respiratory tract (J30-J39)	✗
Chronic lower respiratory diseases (J40-J47)	✗
Diseases of oral cavity, salivary glands and jaws (K00-K14)	✗
Diseases of oesophagus, stomach and duodenum (K20-K31)	✗
Diseases of appendix (K35-K38)	✗
Hernia (K40-K46)	✗
Noninfective enteritis and colitis (K50-K52)	✗
Other diseases of intestines (K55-K63)	✗
Disorders of gallbladder, biliary tract and pancreas (K80-K87)	✗
Infections of the skin and subcutaneous tissue (L00-L08)	✗
Disorders of skin appendages (L60-L75)	✗
Arthropathies (M00-M25)	✗
Dorsopathies (M40-M54)	✗
Soft tissue disorders (M60-M79)	✗
Osteopathies and chondropathies (M80-M94)	✗
Renal tubulo-interstitial diseases (N10-N16)	✗
Urolithiasis (N20-N23)	✗
Other diseases of urinary system (N30-N39)	✗
Diseases of male genital organs (N40-N51)	✗
Disorders of breast (N60-N64)	✗
Inflammatory diseases of female pelvic organs (N70-N77)	✗
Noninflammatory disorders of female genital tract (N80-N98)	✗

Table S16: Inclusion and exclusion criteria for cluster 15. Female ratio: 45%, mean age of patients: 53, mortality: 0%.

Intestinal infectious diseases (A00-A09)	✗
Infections with a predominantly sexual mode of transmission (A50-A64)	✗
Viral infections characterized by skin and mucous membrane lesions (B00-B09)	✗
Malignant neoplasms (C00-C97)	✗
Benign neoplasms (D10-D36)	✗
Neoplasms of uncertain or unknown behaviour (D37-D48)	✗
Disorders of thyroid gland (E00-E07)	✗
Metabolic disorders (E70-E90)	✗
Mental and behavioural disorders due to psychoactive substance use (F10-F19)	✗
Schizophrenia, schizotypal and delusional disorders (F20-F29)	✗
Mood [affective] disorders (F30-F39)	✗
Neurotic, stress-related and somatoform disorders (F40-F48)	✗
Episodic and paroxysmal disorders (G40-G47)	✗
Nerve, nerve root and plexus disorders (G50-G59)	✗
Disorders of eyelid, lacrimal system and orbit (H00-H06)	✗
Disorders of lens (H25-H28)	✗
Disorders of ocular muscles, binocular movement, accommodation and refraction (H49-H52)	✗
Diseases of middle ear and mastoid (H65-H75)	✓
Other disorders of ear (H90-H95)	✗
Hypertensive diseases (I10-I15)	✗
Other forms of heart disease (I30-I52)	✗
Diseases of veins, lymphatic vessels and lymph nodes, not elsewhere classified (I80-I89)	✗
Acute upper respiratory infections (J00-J06)	✗
Influenza and pneumonia (J09-J18)	✗
Other acute lower respiratory infections (J20-J22)	✗
Other diseases of upper respiratory tract (J30-J39)	✗
Chronic lower respiratory diseases (J40-J47)	✗
Diseases of oral cavity, salivary glands and jaws (K00-K14)	✗
Diseases of oesophagus, stomach and duodenum (K20-K31)	✗
Diseases of appendix (K35-K38)	✗
Hernia (K40-K46)	✗
Noninfective enteritis and colitis (K50-K52)	✗
Other diseases of intestines (K55-K63)	✗
Disorders of gallbladder, biliary tract and pancreas (K80-K87)	✗
Infections of the skin and subcutaneous tissue (L00-L08)	✗
Disorders of skin appendages (L60-L75)	✗
Arthropathies (M00-M25)	✗
Dorsopathies (M40-M54)	✗
Soft tissue disorders (M60-M79)	✗
Osteopathies and chondropathies (M80-M94)	✗
Renal tubulo-interstitial diseases (N10-N16)	✗
Urolithiasis (N20-N23)	✗
Other diseases of urinary system (N30-N39)	✗
Diseases of male genital organs (N40-N51)	✗
Disorders of breast (N60-N64)	✗
Inflammatory diseases of female pelvic organs (N70-N77)	✗
Noninflammatory disorders of female genital tract (N80-N98)	✗

Table S17: Inclusion and exclusion criteria for cluster 16. Female ratio: 45%, mean age of patients: 24, mortality: 0%.

Intestinal infectious diseases (A00-A09)	✗
Infections with a predominantly sexual mode of transmission (A50-A64)	✗
Viral infections characterized by skin and mucous membrane lesions (B00-B09)	✗
Malignant neoplasms (C00-C97)	✗
Benign neoplasms (D10-D36)	✗
Neoplasms of uncertain or unknown behaviour (D37-D48)	✗
Disorders of thyroid gland (E00-E07)	✗
Metabolic disorders (E70-E90)	✗
Mental and behavioural disorders due to psychoactive substance use (F10-F19)	✗
Schizophrenia, schizotypal and delusional disorders (F20-F29)	✓
Mood [affective] disorders (F30-F39)	✗
Neurotic, stress-related and somatoform disorders (F40-F48)	✗
Episodic and paroxysmal disorders (G40-G47)	✗
Nerve, nerve root and plexus disorders (G50-G59)	✗
Disorders of eyelid, lacrimal system and orbit (H00-H06)	✗
Disorders of lens (H25-H28)	✗
Disorders of ocular muscles, binocular movement, accommodation and refraction (H49-H52)	✗
Other disorders of ear (H90-H95)	✗
Hypertensive diseases (I10-I15)	✗
Other forms of heart disease (I30-I52)	✗
Diseases of veins, lymphatic vessels and lymph nodes, not elsewhere classified (I80-I89)	✗
Acute upper respiratory infections (J00-J06)	✗
Influenza and pneumonia (J09-J18)	✗
Other acute lower respiratory infections (J20-J22)	✗
Other diseases of upper respiratory tract (J30-J39)	✗
Chronic lower respiratory diseases (J40-J47)	✗
Diseases of oral cavity, salivary glands and jaws (K00-K14)	✗
Diseases of oesophagus, stomach and duodenum (K20-K31)	✗
Diseases of appendix (K35-K38)	✗
Hernia (K40-K46)	✗
Noninfective enteritis and colitis (K50-K52)	✗
Other diseases of intestines (K55-K63)	✗
Disorders of gallbladder, biliary tract and pancreas (K80-K87)	✗
Infections of the skin and subcutaneous tissue (L00-L08)	✗
Disorders of skin appendages (L60-L75)	✗
Arthropathies (M00-M25)	✗
Dorsopathies (M40-M54)	✗
Soft tissue disorders (M60-M79)	✗
Osteopathies and chondropathies (M80-M94)	✗
Renal tubulo-interstitial diseases (N10-N16)	✗
Urolithiasis (N20-N23)	✗
Other diseases of urinary system (N30-N39)	✗
Diseases of male genital organs (N40-N51)	✗
Disorders of breast (N60-N64)	✗
Inflammatory diseases of female pelvic organs (N70-N77)	✗
Noninflammatory disorders of female genital tract (N80-N98)	✗

Table S18: Inclusion and exclusion criteria for cluster 17. Female ratio: 44%, mean age of patients: 41, mortality: 0%.

Intestinal infectious diseases (A00-A09)	✗
Infections with a predominantly sexual mode of transmission (A50-A64)	✓
Viral infections characterized by skin and mucous membrane lesions (B00-B09)	✗
Malignant neoplasms (C00-C97)	✗
Benign neoplasms (D10-D36)	✗
Neoplasms of uncertain or unknown behaviour (D37-D48)	✗
Disorders of thyroid gland (E00-E07)	✗
Metabolic disorders (E70-E90)	✗
Mental and behavioural disorders due to psychoactive substance use (F10-F19)	✗
Mood [affective] disorders (F30-F39)	✗
Neurotic, stress-related and somatoform disorders (F40-F48)	✗
Episodic and paroxysmal disorders (G40-G47)	✗
Nerve, nerve root and plexus disorders (G50-G59)	✗
Disorders of eyelid, lacrimal system and orbit (H00-H06)	✗
Disorders of lens (H25-H28)	✗
Disorders of ocular muscles, binocular movement, accommodation and refraction (H49-H52)	✗
Other disorders of ear (H90-H95)	✗
Hypertensive diseases (I10-I15)	✗
Other forms of heart disease (I30-I52)	✗
Diseases of veins, lymphatic vessels and lymph nodes, not elsewhere classified (I80-I89)	✗
Acute upper respiratory infections (J00-J06)	✗
Influenza and pneumonia (J09-J18)	✗
Other acute lower respiratory infections (J20-J22)	✗
Other diseases of upper respiratory tract (J30-J39)	✗
Chronic lower respiratory diseases (J40-J47)	✗
Diseases of oral cavity, salivary glands and jaws (K00-K14)	✗
Diseases of oesophagus, stomach and duodenum (K20-K31)	✗
Diseases of appendix (K35-K38)	✗
Hernia (K40-K46)	✗
Noninfective enteritis and colitis (K50-K52)	✗
Other diseases of intestines (K55-K63)	✗
Disorders of gallbladder, biliary tract and pancreas (K80-K87)	✗
Infections of the skin and subcutaneous tissue (L00-L08)	✗
Disorders of skin appendages (L60-L75)	✗
Arthropathies (M00-M25)	✗
Dorsopathies (M40-M54)	✗
Soft tissue disorders (M60-M79)	✗
Osteopathies and chondropathies (M80-M94)	✗
Renal tubulo-interstitial diseases (N10-N16)	✗
Urolithiasis (N20-N23)	✗
Other diseases of urinary system (N30-N39)	✗
Diseases of male genital organs (N40-N51)	✗
Disorders of breast (N60-N64)	✗
Inflammatory diseases of female pelvic organs (N70-N77)	✗
Noninflammatory disorders of female genital tract (N80-N98)	✗

Table S19: Inclusion and exclusion criteria for cluster 18. Female ratio: 73%, mean age of patients: 30, mortality: 0%.

Intestinal infectious diseases (A00-A09)	✗
Viral infections characterized by skin and mucous membrane lesions (B00-B09)	✗
Malignant neoplasms (C00-C97)	✗
Benign neoplasms (D10-D36)	✗
Neoplasms of uncertain or unknown behaviour (D37-D48)	✗
Disorders of thyroid gland (E00-E07)	✗
Metabolic disorders (E70-E90)	✗
Mental and behavioural disorders due to psychoactive substance use (F10-F19)	✗
Mood [affective] disorders (F30-F39)	✗
Neurotic, stress-related and somatoform disorders (F40-F48)	✗
Episodic and paroxysmal disorders (G40-G47)	✗
Nerve, nerve root and plexus disorders (G50-G59)	✗
Disorders of eyelid, lacrimal system and orbit (H00-H06)	✗
Disorders of lens (H25-H28)	✗
Disorders of ocular muscles, binocular movement, accommodation and refraction (H49-H52)	✓
Other disorders of ear (H90-H95)	✗
Hypertensive diseases (I10-I15)	✗
Other forms of heart disease (I30-I52)	✗
Diseases of veins, lymphatic vessels and lymph nodes, not elsewhere classified (I80-I89)	✗
Acute upper respiratory infections (J00-J06)	✗
Influenza and pneumonia (J09-J18)	✗
Other acute lower respiratory infections (J20-J22)	✗
Other diseases of upper respiratory tract (J30-J39)	✗
Chronic lower respiratory diseases (J40-J47)	✗
Diseases of oral cavity, salivary glands and jaws (K00-K14)	✗
Diseases of oesophagus, stomach and duodenum (K20-K31)	✗
Diseases of appendix (K35-K38)	✗
Hernia (K40-K46)	✗
Noninfective enteritis and colitis (K50-K52)	✗
Other diseases of intestines (K55-K63)	✗
Disorders of gallbladder, biliary tract and pancreas (K80-K87)	✗
Infections of the skin and subcutaneous tissue (L00-L08)	✗
Disorders of skin appendages (L60-L75)	✗
Arthropathies (M00-M25)	✗
Dorsopathies (M40-M54)	✗
Soft tissue disorders (M60-M79)	✗
Osteopathies and chondropathies (M80-M94)	✗
Renal tubulo-interstitial diseases (N10-N16)	✗
Urolithiasis (N20-N23)	✗
Other diseases of urinary system (N30-N39)	✗
Diseases of male genital organs (N40-N51)	✗
Disorders of breast (N60-N64)	✗
Inflammatory diseases of female pelvic organs (N70-N77)	✗
Noninflammatory disorders of female genital tract (N80-N98)	✗

Table S20: Inclusion and exclusion criteria for cluster 19. Female ratio: 52%, mean age of patients: 28, mortality: 0%.

Intestinal infectious diseases (A00-A09)	✗
Viral infections characterized by skin and mucous membrane lesions (B00-B09)	✗
Malignant neoplasms (C00-C97)	✗
Benign neoplasms (D10-D36)	✗
Neoplasms of uncertain or unknown behaviour (D37-D48)	✓
Disorders of thyroid gland (E00-E07)	✗
Metabolic disorders (E70-E90)	✗
Mental and behavioural disorders due to psychoactive substance use (F10-F19)	✗
Mood [affective] disorders (F30-F39)	✗
Neurotic, stress-related and somatoform disorders (F40-F48)	✗
Episodic and paroxysmal disorders (G40-G47)	✗
Nerve, nerve root and plexus disorders (G50-G59)	✗
Disorders of eyelid, lacrimal system and orbit (H00-H06)	✗
Disorders of lens (H25-H28)	✗
Other disorders of ear (H90-H95)	✗
Hypertensive diseases (I10-I15)	✗
Other forms of heart disease (I30-I52)	✗
Diseases of veins, lymphatic vessels and lymph nodes, not elsewhere classified (I80-I89)	✗
Acute upper respiratory infections (J00-J06)	✗
Influenza and pneumonia (J09-J18)	✗
Other acute lower respiratory infections (J20-J22)	✗
Other diseases of upper respiratory tract (J30-J39)	✗
Chronic lower respiratory diseases (J40-J47)	✗
Diseases of oral cavity, salivary glands and jaws (K00-K14)	✗
Diseases of oesophagus, stomach and duodenum (K20-K31)	✗
Diseases of appendix (K35-K38)	✗
Hernia (K40-K46)	✗
Noninfective enteritis and colitis (K50-K52)	✗
Other diseases of intestines (K55-K63)	✗
Disorders of gallbladder, biliary tract and pancreas (K80-K87)	✗
Infections of the skin and subcutaneous tissue (L00-L08)	✗
Disorders of skin appendages (L60-L75)	✗
Arthropathies (M00-M25)	✗
Dorsopathies (M40-M54)	✗
Soft tissue disorders (M60-M79)	✗
Osteopathies and chondropathies (M80-M94)	✗
Renal tubulo-interstitial diseases (N10-N16)	✗
Urolithiasis (N20-N23)	✗
Other diseases of urinary system (N30-N39)	✗
Diseases of male genital organs (N40-N51)	✗
Disorders of breast (N60-N64)	✗
Inflammatory diseases of female pelvic organs (N70-N77)	✗
Noninflammatory disorders of female genital tract (N80-N98)	✗

Table S21: Inclusion and exclusion criteria for cluster 20. Female ratio: 58%, mean age of patients: 46, mortality: 0%.

Intestinal infectious diseases (A00-A09)	✗
Viral infections characterized by skin and mucous membrane lesions (B00-B09)	✗
Malignant neoplasms (C00-C97)	✗
Benign neoplasms (D10-D36)	✗
Disorders of thyroid gland (E00-E07)	✗
Metabolic disorders (E70-E90)	✗
Mental and behavioural disorders due to psychoactive substance use (F10-F19)	✗
Mood [affective] disorders (F30-F39)	✗
Neurotic, stress-related and somatoform disorders (F40-F48)	✗
Episodic and paroxysmal disorders (G40-G47)	✗
Nerve, nerve root and plexus disorders (G50-G59)	✗
Disorders of eyelid, lacrimal system and orbit (H00-H06)	✗
Disorders of lens (H25-H28)	✗
Other disorders of ear (H90-H95)	✗
Hypertensive diseases (I10-I15)	✗
Other forms of heart disease (I30-I52)	✗
Diseases of veins, lymphatic vessels and lymph nodes, not elsewhere classified (I80-I89)	✗
Acute upper respiratory infections (J00-J06)	✗
Influenza and pneumonia (J09-J18)	✗
Other acute lower respiratory infections (J20-J22)	✗
Other diseases of upper respiratory tract (J30-J39)	✗
Chronic lower respiratory diseases (J40-J47)	✗
Diseases of oral cavity, salivary glands and jaws (K00-K14)	✗
Diseases of oesophagus, stomach and duodenum (K20-K31)	✗
Diseases of appendix (K35-K38)	✗
Hernia (K40-K46)	✗
Noninfective enteritis and colitis (K50-K52)	✗
Other diseases of intestines (K55-K63)	✗
Disorders of gallbladder, biliary tract and pancreas (K80-K87)	✗
Infections of the skin and subcutaneous tissue (L00-L08)	✗
Disorders of skin appendages (L60-L75)	✗
Arthropathies (M00-M25)	✗
Dorsopathies (M40-M54)	✗
Soft tissue disorders (M60-M79)	✗
Osteopathies and chondropathies (M80-M94)	✗
Renal tubulo-interstitial diseases (N10-N16)	✗
Urolithiasis (N20-N23)	✗
Other diseases of urinary system (N30-N39)	✗
Diseases of male genital organs (N40-N51)	✗
Disorders of breast (N60-N64)	✓
Inflammatory diseases of female pelvic organs (N70-N77)	✗
Noninflammatory disorders of female genital tract (N80-N98)	✗

Table S22: Inclusion and exclusion criteria for cluster 21. Female ratio: 79%, mean age of patients: 37, mortality: 0%.

Intestinal infectious diseases (A00-A09)	✗
Viral infections characterized by skin and mucous membrane lesions (B00-B09)	✗
Malignant neoplasms (C00-C97)	✗
Benign neoplasms (D10-D36)	✗
Disorders of thyroid gland (E00-E07)	✗
Metabolic disorders (E70-E90)	✗
Mental and behavioural disorders due to psychoactive substance use (F10-F19)	✗
Mood [affective] disorders (F30-F39)	✗
Neurotic, stress-related and somatoform disorders (F40-F48)	✗
Episodic and paroxysmal disorders (G40-G47)	✗
Nerve, nerve root and plexus disorders (G50-G59)	✗
Disorders of eyelid, lacrimal system and orbit (H00-H06)	✓
Disorders of lens (H25-H28)	✗
Other disorders of ear (H90-H95)	✗
Hypertensive diseases (I10-I15)	✗
Other forms of heart disease (I30-I52)	✗
Diseases of veins, lymphatic vessels and lymph nodes, not elsewhere classified (I80-I89)	✗
Acute upper respiratory infections (J00-J06)	✗
Influenza and pneumonia (J09-J18)	✗
Other acute lower respiratory infections (J20-J22)	✗
Other diseases of upper respiratory tract (J30-J39)	✗
Chronic lower respiratory diseases (J40-J47)	✗
Diseases of oral cavity, salivary glands and jaws (K00-K14)	✗
Diseases of oesophagus, stomach and duodenum (K20-K31)	✗
Diseases of appendix (K35-K38)	✗
Hernia (K40-K46)	✗
Noninfective enteritis and colitis (K50-K52)	✗
Other diseases of intestines (K55-K63)	✗
Disorders of gallbladder, biliary tract and pancreas (K80-K87)	✗
Infections of the skin and subcutaneous tissue (L00-L08)	✗
Disorders of skin appendages (L60-L75)	✗
Arthropathies (M00-M25)	✗
Dorsopathies (M40-M54)	✗
Soft tissue disorders (M60-M79)	✗
Osteopathies and chondropathies (M80-M94)	✗
Renal tubulo-interstitial diseases (N10-N16)	✗
Urolithiasis (N20-N23)	✗
Other diseases of urinary system (N30-N39)	✗
Diseases of male genital organs (N40-N51)	✗
Inflammatory diseases of female pelvic organs (N70-N77)	✗
Noninflammatory disorders of female genital tract (N80-N98)	✗

Table S23: Inclusion and exclusion criteria for cluster 22. Female ratio: 60%, mean age of patients: 41, mortality: 0%.

Intestinal infectious diseases (A00-A09)	✗
Viral infections characterized by skin and mucous membrane lesions (B00-B09)	✗
Malignant neoplasms (C00-C97)	✗
Benign neoplasms (D10-D36)	✗
Disorders of thyroid gland (E00-E07)	✗
Metabolic disorders (E70-E90)	✗
Mental and behavioural disorders due to psychoactive substance use (F10-F19)	✗
Mood [affective] disorders (F30-F39)	✗
Neurotic, stress-related and somatoform disorders (F40-F48)	✗
Episodic and paroxysmal disorders (G40-G47)	✗
Nerve, nerve root and plexus disorders (G50-G59)	✗
Disorders of lens (H25-H28)	✗
Other disorders of ear (H90-H95)	✗
Hypertensive diseases (I10-I15)	✗
Other forms of heart disease (I30-I52)	✗
Diseases of veins, lymphatic vessels and lymph nodes, not elsewhere classified (I80-I89)	✗
Acute upper respiratory infections (J00-J06)	✗
Influenza and pneumonia (J09-J18)	✗
Other acute lower respiratory infections (J20-J22)	✗
Other diseases of upper respiratory tract (J30-J39)	✗
Chronic lower respiratory diseases (J40-J47)	✗
Diseases of oral cavity, salivary glands and jaws (K00-K14)	✗
Diseases of oesophagus, stomach and duodenum (K20-K31)	✗
Diseases of appendix (K35-K38)	✗
Hernia (K40-K46)	✗
Noninfective enteritis and colitis (K50-K52)	✗
Other diseases of intestines (K55-K63)	✗
Disorders of gallbladder, biliary tract and pancreas (K80-K87)	✗
Infections of the skin and subcutaneous tissue (L00-L08)	✗
Disorders of skin appendages (L60-L75)	✗
Arthropathies (M00-M25)	✗
Dorsopathies (M40-M54)	✗
Soft tissue disorders (M60-M79)	✗
Osteopathies and chondropathies (M80-M94)	✗
Renal tubulo-interstitial diseases (N10-N16)	✓
Urolithiasis (N20-N23)	✗
Other diseases of urinary system (N30-N39)	✗
Diseases of male genital organs (N40-N51)	✗
Inflammatory diseases of female pelvic organs (N70-N77)	✗
Noninflammatory disorders of female genital tract (N80-N98)	✗

Table S24: Inclusion and exclusion criteria for cluster 23. Female ratio: 75%, mean age of patients: 27, mortality: 0%.

Intestinal infectious diseases (A00-A09)	✗
Viral infections characterized by skin and mucous membrane lesions (B00-B09)	✗
Malignant neoplasms (C00-C97)	✗
Benign neoplasms (D10-D36)	✗
Disorders of thyroid gland (E00-E07)	✗
Metabolic disorders (E70-E90)	✗
Mental and behavioural disorders due to psychoactive substance use (F10-F19)	✗
Mood [affective] disorders (F30-F39)	✗
Neurotic, stress-related and somatoform disorders (F40-F48)	✗
Episodic and paroxysmal disorders (G40-G47)	✗
Nerve, nerve root and plexus disorders (G50-G59)	✗
Disorders of lens (H25-H28)	✗
Other disorders of ear (H90-H95)	✗
Hypertensive diseases (I10-I15)	✗
Other forms of heart disease (I30-I52)	✗
Diseases of veins, lymphatic vessels and lymph nodes, not elsewhere classified (I80-I89)	✗
Acute upper respiratory infections (J00-J06)	✗
Influenza and pneumonia (J09-J18)	✗
Other acute lower respiratory infections (J20-J22)	✗
Other diseases of upper respiratory tract (J30-J39)	✗
Chronic lower respiratory diseases (J40-J47)	✗
Diseases of oral cavity, salivary glands and jaws (K00-K14)	✗
Diseases of oesophagus, stomach and duodenum (K20-K31)	✗
Diseases of appendix (K35-K38)	✗
Hernia (K40-K46)	✗
Noninfective enteritis and colitis (K50-K52)	✗
Other diseases of intestines (K55-K63)	✗
Disorders of gallbladder, biliary tract and pancreas (K80-K87)	✗
Infections of the skin and subcutaneous tissue (L00-L08)	✗
Disorders of skin appendages (L60-L75)	✗
Arthropathies (M00-M25)	✗
Dorsopathies (M40-M54)	✗
Soft tissue disorders (M60-M79)	✗
Osteopathies and chondropathies (M80-M94)	✓
Urolithiasis (N20-N23)	✗
Other diseases of urinary system (N30-N39)	✗
Diseases of male genital organs (N40-N51)	✗
Inflammatory diseases of female pelvic organs (N70-N77)	✗
Noninflammatory disorders of female genital tract (N80-N98)	✗

Table S25: Inclusion and exclusion criteria for cluster 24. Female ratio: 43%, mean age of patients: 43, mortality: 0%.

Intestinal infectious diseases (A00-A09)	✗
Viral infections characterized by skin and mucous membrane lesions (B00-B09)	✓
Malignant neoplasms (C00-C97)	✗
Benign neoplasms (D10-D36)	✗
Disorders of thyroid gland (E00-E07)	✗
Metabolic disorders (E70-E90)	✗
Mental and behavioural disorders due to psychoactive substance use (F10-F19)	✗
Mood [affective] disorders (F30-F39)	✗
Neurotic, stress-related and somatoform disorders (F40-F48)	✗
Episodic and paroxysmal disorders (G40-G47)	✗
Nerve, nerve root and plexus disorders (G50-G59)	✗
Disorders of lens (H25-H28)	✗
Other disorders of ear (H90-H95)	✗
Hypertensive diseases (I10-I15)	✗
Other forms of heart disease (I30-I52)	✗
Diseases of veins, lymphatic vessels and lymph nodes, not elsewhere classified (I80-I89)	✗
Acute upper respiratory infections (J00-J06)	✗
Influenza and pneumonia (J09-J18)	✗
Other acute lower respiratory infections (J20-J22)	✗
Other diseases of upper respiratory tract (J30-J39)	✗
Chronic lower respiratory diseases (J40-J47)	✗
Diseases of oral cavity, salivary glands and jaws (K00-K14)	✗
Diseases of oesophagus, stomach and duodenum (K20-K31)	✗
Diseases of appendix (K35-K38)	✗
Hernia (K40-K46)	✗
Noninfective enteritis and colitis (K50-K52)	✗
Other diseases of intestines (K55-K63)	✗
Disorders of gallbladder, biliary tract and pancreas (K80-K87)	✗
Infections of the skin and subcutaneous tissue (L00-L08)	✗
Disorders of skin appendages (L60-L75)	✗
Arthropathies (M00-M25)	✗
Dorsopathies (M40-M54)	✗
Soft tissue disorders (M60-M79)	✗
Urolithiasis (N20-N23)	✗
Other diseases of urinary system (N30-N39)	✗
Diseases of male genital organs (N40-N51)	✗
Inflammatory diseases of female pelvic organs (N70-N77)	✗
Noninflammatory disorders of female genital tract (N80-N98)	✗

Table S26: Inclusion and exclusion criteria for cluster 25. Female ratio: 54%, mean age of patients: 22, mortality: 0%.

Intestinal infectious diseases (A00-A09)	✗
Malignant neoplasms (C00-C97)	✗
Benign neoplasms (D10-D36)	✗
Disorders of thyroid gland (E00-E07)	✗
Metabolic disorders (E70-E90)	✓
Mental and behavioural disorders due to psychoactive substance use (F10-F19)	✗
Mood [affective] disorders (F30-F39)	✗
Neurotic, stress-related and somatoform disorders (F40-F48)	✗
Episodic and paroxysmal disorders (G40-G47)	✗
Nerve, nerve root and plexus disorders (G50-G59)	✗
Disorders of lens (H25-H28)	✗
Other disorders of ear (H90-H95)	✗
Hypertensive diseases (I10-I15)	✗
Other forms of heart disease (I30-I52)	✗
Diseases of veins, lymphatic vessels and lymph nodes, not elsewhere classified (I80-I89)	✗
Acute upper respiratory infections (J00-J06)	✗
Influenza and pneumonia (J09-J18)	✗
Other acute lower respiratory infections (J20-J22)	✗
Other diseases of upper respiratory tract (J30-J39)	✗
Chronic lower respiratory diseases (J40-J47)	✗
Diseases of oral cavity, salivary glands and jaws (K00-K14)	✗
Diseases of oesophagus, stomach and duodenum (K20-K31)	✗
Diseases of appendix (K35-K38)	✗
Hernia (K40-K46)	✗
Noninfective enteritis and colitis (K50-K52)	✗
Other diseases of intestines (K55-K63)	✗
Disorders of gallbladder, biliary tract and pancreas (K80-K87)	✗
Infections of the skin and subcutaneous tissue (L00-L08)	✗
Disorders of skin appendages (L60-L75)	✗
Arthropathies (M00-M25)	✗
Dorsopathies (M40-M54)	✗
Soft tissue disorders (M60-M79)	✗
Urolithiasis (N20-N23)	✗
Other diseases of urinary system (N30-N39)	✗
Diseases of male genital organs (N40-N51)	✗
Inflammatory diseases of female pelvic organs (N70-N77)	✗
Noninflammatory disorders of female genital tract (N80-N98)	✗

Table S27: Inclusion and exclusion criteria for cluster 26. Female ratio: 44%, mean age of patients: 44, mortality: 0%.

Intestinal infectious diseases (A00-A09)	✗
Malignant neoplasms (C00-C97)	✗
Benign neoplasms (D10-D36)	✗
Disorders of thyroid gland (E00-E07)	✗
Mental and behavioural disorders due to psychoactive substance use (F10-F19)	✗
Mood [affective] disorders (F30-F39)	✓
Neurotic, stress-related and somatoform disorders (F40-F48)	✗
Episodic and paroxysmal disorders (G40-G47)	✗
Nerve, nerve root and plexus disorders (G50-G59)	✗
Disorders of lens (H25-H28)	✗
Other disorders of ear (H90-H95)	✗
Hypertensive diseases (I10-I15)	✗
Other forms of heart disease (I30-I52)	✗
Diseases of veins, lymphatic vessels and lymph nodes, not elsewhere classified (I80-I89)	✗
Acute upper respiratory infections (J00-J06)	✗
Influenza and pneumonia (J09-J18)	✗
Other acute lower respiratory infections (J20-J22)	✗
Other diseases of upper respiratory tract (J30-J39)	✗
Chronic lower respiratory diseases (J40-J47)	✗
Diseases of oral cavity, salivary glands and jaws (K00-K14)	✗
Diseases of oesophagus, stomach and duodenum (K20-K31)	✗
Diseases of appendix (K35-K38)	✗
Hernia (K40-K46)	✗
Noninfective enteritis and colitis (K50-K52)	✗
Other diseases of intestines (K55-K63)	✗
Disorders of gallbladder, biliary tract and pancreas (K80-K87)	✗
Infections of the skin and subcutaneous tissue (L00-L08)	✗
Disorders of skin appendages (L60-L75)	✗
Arthropathies (M00-M25)	✗
Dorsopathies (M40-M54)	✗
Soft tissue disorders (M60-M79)	✗
Urolithiasis (N20-N23)	✗
Other diseases of urinary system (N30-N39)	✗
Diseases of male genital organs (N40-N51)	✗
Inflammatory diseases of female pelvic organs (N70-N77)	✗
Noninflammatory disorders of female genital tract (N80-N98)	✗

Table S28: Inclusion and exclusion criteria for cluster 27. Female ratio: 61%, mean age of patients: 42, mortality: 0%.

Intestinal infectious diseases (A00-A09)	✗
Malignant neoplasms (C00-C97)	✗
Benign neoplasms (D10-D36)	✗
Disorders of thyroid gland (E00-E07)	✗
Mental and behavioural disorders due to psychoactive substance use (F10-F19)	✗
Neurotic, stress-related and somatoform disorders (F40-F48)	✗
Episodic and paroxysmal disorders (G40-G47)	✗
Nerve, nerve root and plexus disorders (G50-G59)	✗
Disorders of lens (H25-H28)	✗
Other disorders of ear (H90-H95)	✗
Hypertensive diseases (I10-I15)	✗
Other forms of heart disease (I30-I52)	✗
Diseases of veins, lymphatic vessels and lymph nodes, not elsewhere classified (I80-I89)	✗
Acute upper respiratory infections (J00-J06)	✗
Influenza and pneumonia (J09-J18)	✗
Other acute lower respiratory infections (J20-J22)	✗
Other diseases of upper respiratory tract (J30-J39)	✗
Chronic lower respiratory diseases (J40-J47)	✓
Diseases of oral cavity, salivary glands and jaws (K00-K14)	✗
Diseases of oesophagus, stomach and duodenum (K20-K31)	✗
Diseases of appendix (K35-K38)	✗
Hernia (K40-K46)	✗
Noninfective enteritis and colitis (K50-K52)	✗
Other diseases of intestines (K55-K63)	✗
Disorders of gallbladder, biliary tract and pancreas (K80-K87)	✗
Infections of the skin and subcutaneous tissue (L00-L08)	✗
Disorders of skin appendages (L60-L75)	✗
Arthropathies (M00-M25)	✗
Dorsopathies (M40-M54)	✗
Soft tissue disorders (M60-M79)	✗
Urolithiasis (N20-N23)	✗
Other diseases of urinary system (N30-N39)	✗
Diseases of male genital organs (N40-N51)	✗
Inflammatory diseases of female pelvic organs (N70-N77)	✗
Noninflammatory disorders of female genital tract (N80-N98)	✗

Table S29: Inclusion and exclusion criteria for cluster 28. Female ratio: 44%, mean age of patients: 33, mortality: 0%.

Intestinal infectious diseases (A00-A09)	✗
Malignant neoplasms (C00-C97)	✗
Benign neoplasms (D10-D36)	✗
Disorders of thyroid gland (E00-E07)	✗
Mental and behavioural disorders due to psychoactive substance use (F10-F19)	✗
Neurotic, stress-related and somatoform disorders (F40-F48)	✗
Episodic and paroxysmal disorders (G40-G47)	✗
Nerve, nerve root and plexus disorders (G50-G59)	✗
Disorders of lens (H25-H28)	✗
Other disorders of ear (H90-H95)	✗
Hypertensive diseases (I10-I15)	✗
Other forms of heart disease (I30-I52)	✗
Diseases of veins, lymphatic vessels and lymph nodes, not elsewhere classified (I80-I89)	✗
Acute upper respiratory infections (J00-J06)	✗
Influenza and pneumonia (J09-J18)	✗
Other acute lower respiratory infections (J20-J22)	✗
Other diseases of upper respiratory tract (J30-J39)	✗
Diseases of oral cavity, salivary glands and jaws (K00-K14)	✗
Diseases of oesophagus, stomach and duodenum (K20-K31)	✗
Diseases of appendix (K35-K38)	✗
Hernia (K40-K46)	✗
Noninfective enteritis and colitis (K50-K52)	✗
Other diseases of intestines (K55-K63)	✗
Disorders of gallbladder, biliary tract and pancreas (K80-K87)	✗
Infections of the skin and subcutaneous tissue (L00-L08)	✗
Disorders of skin appendages (L60-L75)	✗
Arthropathies (M00-M25)	✗
Dorsopathies (M40-M54)	✗
Soft tissue disorders (M60-M79)	✗
Urolithiasis (N20-N23)	✗
Other diseases of urinary system (N30-N39)	✗
Diseases of male genital organs (N40-N51)	✗
Inflammatory diseases of female pelvic organs (N70-N77)	✓
Noninflammatory disorders of female genital tract (N80-N98)	✗

Table S30: Inclusion and exclusion criteria for cluster 29. Female ratio: 100%, mean age of patients: 35, mortality: 0%.

Intestinal infectious diseases (A00-A09)	✗
Malignant neoplasms (C00-C97)	✗
Benign neoplasms (D10-D36)	✗
Disorders of thyroid gland (E00-E07)	✗
Mental and behavioural disorders due to psychoactive substance use (F10-F19)	✗
Neurotic, stress-related and somatoform disorders (F40-F48)	✗
Episodic and paroxysmal disorders (G40-G47)	✗
Nerve, nerve root and plexus disorders (G50-G59)	✗
Disorders of lens (H25-H28)	✗
Other disorders of ear (H90-H95)	✗
Hypertensive diseases (I10-I15)	✗
Other forms of heart disease (I30-I52)	✗
Diseases of veins, lymphatic vessels and lymph nodes, not elsewhere classified (I80-I89)	✗
Acute upper respiratory infections (J00-J06)	✗
Influenza and pneumonia (J09-J18)	✗
Other acute lower respiratory infections (J20-J22)	✗
Other diseases of upper respiratory tract (J30-J39)	✗
Diseases of oral cavity, salivary glands and jaws (K00-K14)	✗
Diseases of oesophagus, stomach and duodenum (K20-K31)	✗
Diseases of appendix (K35-K38)	✗
Hernia (K40-K46)	✗
Noninfective enteritis and colitis (K50-K52)	✗
Other diseases of intestines (K55-K63)	✗
Disorders of gallbladder, biliary tract and pancreas (K80-K87)	✗
Infections of the skin and subcutaneous tissue (L00-L08)	✗
Disorders of skin appendages (L60-L75)	✓
Arthropathies (M00-M25)	✗
Dorsopathies (M40-M54)	✗
Soft tissue disorders (M60-M79)	✗
Urolithiasis (N20-N23)	✗
Other diseases of urinary system (N30-N39)	✗
Diseases of male genital organs (N40-N51)	✗
Noninflammatory disorders of female genital tract (N80-N98)	✗

Table S31: Inclusion and exclusion criteria for cluster 30. Female ratio: 38%, mean age of patients: 34, mortality: 0%.

Intestinal infectious diseases (A00-A09)	✗
Malignant neoplasms (C00-C97)	✗
Benign neoplasms (D10-D36)	✗
Disorders of thyroid gland (E00-E07)	✗
Mental and behavioural disorders due to psychoactive substance use (F10-F19)	✗
Neurotic, stress-related and somatoform disorders (F40-F48)	✗
Episodic and paroxysmal disorders (G40-G47)	✗
Nerve, nerve root and plexus disorders (G50-G59)	✗
Disorders of lens (H25-H28)	✗
Other disorders of ear (H90-H95)	✓
Hypertensive diseases (I10-I15)	✗
Other forms of heart disease (I30-I52)	✗
Diseases of veins, lymphatic vessels and lymph nodes, not elsewhere classified (I80-I89)	✗
Acute upper respiratory infections (J00-J06)	✗
Influenza and pneumonia (J09-J18)	✗
Other acute lower respiratory infections (J20-J22)	✗
Other diseases of upper respiratory tract (J30-J39)	✗
Diseases of oral cavity, salivary glands and jaws (K00-K14)	✗
Diseases of oesophagus, stomach and duodenum (K20-K31)	✗
Diseases of appendix (K35-K38)	✗
Hernia (K40-K46)	✗
Noninfective enteritis and colitis (K50-K52)	✗
Other diseases of intestines (K55-K63)	✗
Disorders of gallbladder, biliary tract and pancreas (K80-K87)	✗
Infections of the skin and subcutaneous tissue (L00-L08)	✗
Arthropathies (M00-M25)	✗
Dorsopathies (M40-M54)	✗
Soft tissue disorders (M60-M79)	✗
Urolithiasis (N20-N23)	✗
Other diseases of urinary system (N30-N39)	✗
Diseases of male genital organs (N40-N51)	✗
Noninflammatory disorders of female genital tract (N80-N98)	✗

Table S32: Inclusion and exclusion criteria for cluster 31. Female ratio: 43%, mean age of patients: 40, mortality: 0%.

Intestinal infectious diseases (A00-A09)	✗
Malignant neoplasms (C00-C97)	✗
Benign neoplasms (D10-D36)	✗
Disorders of thyroid gland (E00-E07)	✓
Mental and behavioural disorders due to psychoactive substance use (F10-F19)	✗
Neurotic, stress-related and somatoform disorders (F40-F48)	✗
Episodic and paroxysmal disorders (G40-G47)	✗
Nerve, nerve root and plexus disorders (G50-G59)	✗
Disorders of lens (H25-H28)	✗
Hypertensive diseases (I10-I15)	✗
Other forms of heart disease (I30-I52)	✗
Diseases of veins, lymphatic vessels and lymph nodes, not elsewhere classified (I80-I89)	✗
Acute upper respiratory infections (J00-J06)	✗
Influenza and pneumonia (J09-J18)	✗
Other acute lower respiratory infections (J20-J22)	✗
Other diseases of upper respiratory tract (J30-J39)	✗
Diseases of oral cavity, salivary glands and jaws (K00-K14)	✗
Diseases of oesophagus, stomach and duodenum (K20-K31)	✗
Diseases of appendix (K35-K38)	✗
Hernia (K40-K46)	✗
Noninfective enteritis and colitis (K50-K52)	✗
Other diseases of intestines (K55-K63)	✗
Disorders of gallbladder, biliary tract and pancreas (K80-K87)	✗
Infections of the skin and subcutaneous tissue (L00-L08)	✗
Arthropathies (M00-M25)	✗
Dorsopathies (M40-M54)	✗
Soft tissue disorders (M60-M79)	✗
Urolithiasis (N20-N23)	✗
Other diseases of urinary system (N30-N39)	✗
Diseases of male genital organs (N40-N51)	✗
Noninflammatory disorders of female genital tract (N80-N98)	✗

Table S33: Inclusion and exclusion criteria for cluster 32. Female ratio: 78%, mean age of patients: 48, mortality: 0%.

Intestinal infectious diseases (A00-A09)	✗
Malignant neoplasms (C00-C97)	✗
Benign neoplasms (D10-D36)	✗
Mental and behavioural disorders due to psychoactive substance use (F10-F19)	✗
Neurotic, stress-related and somatoform disorders (F40-F48)	✗
Episodic and paroxysmal disorders (G40-G47)	✗
Nerve, nerve root and plexus disorders (G50-G59)	✗
Disorders of lens (H25-H28)	✗
Hypertensive diseases (I10-I15)	✗
Other forms of heart disease (I30-I52)	✗
Diseases of veins, lymphatic vessels and lymph nodes, not elsewhere classified (I80-I89)	✗
Acute upper respiratory infections (J00-J06)	✗
Influenza and pneumonia (J09-J18)	✗
Other acute lower respiratory infections (J20-J22)	✓
Other diseases of upper respiratory tract (J30-J39)	✗
Diseases of oral cavity, salivary glands and jaws (K00-K14)	✗
Diseases of oesophagus, stomach and duodenum (K20-K31)	✗
Diseases of appendix (K35-K38)	✗
Hernia (K40-K46)	✗
Noninfective enteritis and colitis (K50-K52)	✗
Other diseases of intestines (K55-K63)	✗
Disorders of gallbladder, biliary tract and pancreas (K80-K87)	✗
Infections of the skin and subcutaneous tissue (L00-L08)	✗
Arthropathies (M00-M25)	✗
Dorsopathies (M40-M54)	✗
Soft tissue disorders (M60-M79)	✗
Urolithiasis (N20-N23)	✗
Other diseases of urinary system (N30-N39)	✗
Diseases of male genital organs (N40-N51)	✗
Noninflammatory disorders of female genital tract (N80-N98)	✗

Table S34: Inclusion and exclusion criteria for cluster 33. Female ratio: 45%, mean age of patients: 14, mortality: 0%.

Intestinal infectious diseases (A00-A09)	✗
Malignant neoplasms (C00-C97)	✗
Benign neoplasms (D10-D36)	✗
Mental and behavioural disorders due to psychoactive substance use (F10-F19)	✗
Neurotic, stress-related and somatoform disorders (F40-F48)	✗
Episodic and paroxysmal disorders (G40-G47)	✗
Nerve, nerve root and plexus disorders (G50-G59)	✓
Disorders of lens (H25-H28)	✗
Hypertensive diseases (I10-I15)	✗
Other forms of heart disease (I30-I52)	✗
Diseases of veins, lymphatic vessels and lymph nodes, not elsewhere classified (I80-I89)	✗
Acute upper respiratory infections (J00-J06)	✗
Influenza and pneumonia (J09-J18)	✗
Other diseases of upper respiratory tract (J30-J39)	✗
Diseases of oral cavity, salivary glands and jaws (K00-K14)	✗
Diseases of oesophagus, stomach and duodenum (K20-K31)	✗
Diseases of appendix (K35-K38)	✗
Hernia (K40-K46)	✗
Noninfective enteritis and colitis (K50-K52)	✗
Other diseases of intestines (K55-K63)	✗
Disorders of gallbladder, biliary tract and pancreas (K80-K87)	✗
Infections of the skin and subcutaneous tissue (L00-L08)	✗
Arthropathies (M00-M25)	✗
Dorsopathies (M40-M54)	✗
Soft tissue disorders (M60-M79)	✗
Urolithiasis (N20-N23)	✗
Other diseases of urinary system (N30-N39)	✗
Diseases of male genital organs (N40-N51)	✗
Noninflammatory disorders of female genital tract (N80-N98)	✗

Table S35: Inclusion and exclusion criteria for cluster 34. Female ratio: 58%, mean age of patients: 45, mortality: 0%.

Intestinal infectious diseases (A00-A09)	✗
Malignant neoplasms (C00-C97)	✗
Benign neoplasms (D10-D36)	✗
Mental and behavioural disorders due to psychoactive substance use (F10-F19)	✗
Neurotic, stress-related and somatoform disorders (F40-F48)	✗
Episodic and paroxysmal disorders (G40-G47)	✗
Disorders of lens (H25-H28)	✗
Hypertensive diseases (I10-I15)	✗
Other forms of heart disease (I30-I52)	✗
Diseases of veins, lymphatic vessels and lymph nodes, not elsewhere classified (I80-I89)	✗
Acute upper respiratory infections (J00-J06)	✗
Influenza and pneumonia (J09-J18)	✗
Other diseases of upper respiratory tract (J30-J39)	✗
Diseases of oral cavity, salivary glands and jaws (K00-K14)	✗
Diseases of oesophagus, stomach and duodenum (K20-K31)	✓
Diseases of appendix (K35-K38)	✗
Hernia (K40-K46)	✗
Noninfective enteritis and colitis (K50-K52)	✗
Other diseases of intestines (K55-K63)	✗
Disorders of gallbladder, biliary tract and pancreas (K80-K87)	✗
Infections of the skin and subcutaneous tissue (L00-L08)	✗
Arthropathies (M00-M25)	✗
Dorsopathies (M40-M54)	✗
Soft tissue disorders (M60-M79)	✗
Urolithiasis (N20-N23)	✗
Other diseases of urinary system (N30-N39)	✗
Diseases of male genital organs (N40-N51)	✗
Noninflammatory disorders of female genital tract (N80-N98)	✗

Table S36: Inclusion and exclusion criteria for cluster 35. Female ratio: 48%, mean age of patients: 42, mortality: 0%.

Intestinal infectious diseases (A00-A09)	✗
Malignant neoplasms (C00-C97)	✗
Benign neoplasms (D10-D36)	✗
Mental and behavioural disorders due to psychoactive substance use (F10-F19)	✗
Neurotic, stress-related and somatoform disorders (F40-F48)	✗
Episodic and paroxysmal disorders (G40-G47)	✗
Disorders of lens (H25-H28)	✗
Hypertensive diseases (I10-I15)	✗
Other forms of heart disease (I30-I52)	✗
Diseases of veins, lymphatic vessels and lymph nodes, not elsewhere classified (I80-I89)	✗
Acute upper respiratory infections (J00-J06)	✗
Influenza and pneumonia (J09-J18)	✗
Other diseases of upper respiratory tract (J30-J39)	✗
Diseases of oral cavity, salivary glands and jaws (K00-K14)	✗
Diseases of appendix (K35-K38)	✗
Hernia (K40-K46)	✗
Noninfective enteritis and colitis (K50-K52)	✓
Other diseases of intestines (K55-K63)	✗
Disorders of gallbladder, biliary tract and pancreas (K80-K87)	✗
Infections of the skin and subcutaneous tissue (L00-L08)	✗
Arthropathies (M00-M25)	✗
Dorsopathies (M40-M54)	✗
Soft tissue disorders (M60-M79)	✗
Urolithiasis (N20-N23)	✗
Other diseases of urinary system (N30-N39)	✗
Diseases of male genital organs (N40-N51)	✗
Noninflammatory disorders of female genital tract (N80-N98)	✗

Table S37: Inclusion and exclusion criteria for cluster 36. Female ratio: 55%, mean age of patients: 30, mortality: 0%.

238

Intestinal infectious diseases (A00-A09)	✗
Malignant neoplasms (C00-C97)	✗
Benign neoplasms (D10-D36)	✗
Mental and behavioural disorders due to psychoactive substance use (F10-F19)	✗
Neurotic, stress-related and somatoform disorders (F40-F48)	✗
Episodic and paroxysmal disorders (G40-G47)	✗
Disorders of lens (H25-H28)	✗
Hypertensive diseases (I10-I15)	✗
Other forms of heart disease (I30-I52)	✗
Diseases of veins, lymphatic vessels and lymph nodes, not elsewhere classified (I80-I89)	✗
Acute upper respiratory infections (J00-J06)	✗
Influenza and pneumonia (J09-J18)	✓
Other diseases of upper respiratory tract (J30-J39)	✗
Diseases of oral cavity, salivary glands and jaws (K00-K14)	✗
Diseases of appendix (K35-K38)	✗
Hernia (K40-K46)	✗
Other diseases of intestines (K55-K63)	✗
Disorders of gallbladder, biliary tract and pancreas (K80-K87)	✗
Infections of the skin and subcutaneous tissue (L00-L08)	✗
Arthropathies (M00-M25)	✗
Dorsopathies (M40-M54)	✗
Soft tissue disorders (M60-M79)	✗
Urolithiasis (N20-N23)	✗
Other diseases of urinary system (N30-N39)	✗
Diseases of male genital organs (N40-N51)	✗
Noninflammatory disorders of female genital tract (N80-N98)	✗

239 Table S38: Inclusion and exclusion criteria for cluster 37. Female ratio: 47%, mean age of patients: 33, mortality: 1%.

240

Intestinal infectious diseases (A00-A09)	✗
Malignant neoplasms (C00-C97)	✗
Benign neoplasms (D10-D36)	✗
Mental and behavioural disorders due to psychoactive substance use (F10-F19)	✗
Neurotic, stress-related and somatoform disorders (F40-F48)	✗
Episodic and paroxysmal disorders (G40-G47)	✗
Disorders of lens (H25-H28)	✗
Hypertensive diseases (I10-I15)	✗
Other forms of heart disease (I30-I52)	✗
Diseases of veins, lymphatic vessels and lymph nodes, not elsewhere classified (I80-I89)	✗
Acute upper respiratory infections (J00-J06)	✗
Other diseases of upper respiratory tract (J30-J39)	✗
Diseases of oral cavity, salivary glands and jaws (K00-K14)	✗
Diseases of appendix (K35-K38)	✗
Hernia (K40-K46)	✗
Other diseases of intestines (K55-K63)	✗
Disorders of gallbladder, biliary tract and pancreas (K80-K87)	✗
Infections of the skin and subcutaneous tissue (L00-L08)	✗
Arthropathies (M00-M25)	✗
Dorsopathies (M40-M54)	✗
Soft tissue disorders (M60-M79)	✗
Urolithiasis (N20-N23)	✗
Other diseases of urinary system (N30-N39)	✓
Diseases of male genital organs (N40-N51)	✗
Noninflammatory disorders of female genital tract (N80-N98)	✗

241 Table S39: Inclusion and exclusion criteria for cluster 38. Female ratio: 79%, mean age of patients: 37, mortality: 0%.

242

Intestinal infectious diseases (A00-A09)	✗
Malignant neoplasms (C00-C97)	✗
Benign neoplasms (D10-D36)	✗
Mental and behavioural disorders due to psychoactive substance use (F10-F19)	✗
Neurotic, stress-related and somatoform disorders (F40-F48)	✗
Episodic and paroxysmal disorders (G40-G47)	✗
Disorders of lens (H25-H28)	✗
Hypertensive diseases (I10-I15)	✗
Other forms of heart disease (I30-I52)	✗
Diseases of veins, lymphatic vessels and lymph nodes, not elsewhere classified (I80-I89)	✗
Acute upper respiratory infections (J00-J06)	✗
Other diseases of upper respiratory tract (J30-J39)	✗
Diseases of oral cavity, salivary glands and jaws (K00-K14)	✗
Diseases of appendix (K35-K38)	✗
Hernia (K40-K46)	✗
Other diseases of intestines (K55-K63)	✗
Disorders of gallbladder, biliary tract and pancreas (K80-K87)	✗
Infections of the skin and subcutaneous tissue (L00-L08)	✗
Arthropathies (M00-M25)	✗
Dorsopathies (M40-M54)	✗
Soft tissue disorders (M60-M79)	✗
Renal tubulo-interstitial diseases (N10-N16)	✗
Urolithiasis (N20-N23)	✓
Diseases of male genital organs (N40-N51)	✗
Noninflammatory disorders of female genital tract (N80-N98)	✗

243 Table S40: Inclusion and exclusion criteria for cluster 39. Female ratio: 30%, mean age of patients: 44, mortality: 0%.

244

Intestinal infectious diseases (A00-A09)	✗
Malignant neoplasms (C00-C97)	✗
Benign neoplasms (D10-D36)	✗
Mental and behavioural disorders due to psychoactive substance use (F10-F19)	✗
Neurotic, stress-related and somatoform disorders (F40-F48)	✗
Episodic and paroxysmal disorders (G40-G47)	✗
Disorders of lens (H25-H28)	✗
Hypertensive diseases (I10-I15)	✗
Other forms of heart disease (I30-I52)	✗
Diseases of veins, lymphatic vessels and lymph nodes, not elsewhere classified (I80-I89)	✗
Acute upper respiratory infections (J00-J06)	✗
Other diseases of upper respiratory tract (J30-J39)	✗
Diseases of oral cavity, salivary glands and jaws (K00-K14)	✗
Diseases of appendix (K35-K38)	✗
Hernia (K40-K46)	✗
Other diseases of intestines (K55-K63)	✗
Disorders of gallbladder, biliary tract and pancreas (K80-K87)	✗
Infections of the skin and subcutaneous tissue (L00-L08)	✗
Arthropathies (M00-M25)	✗
Dorsopathies (M40-M54)	✗
Soft tissue disorders (M60-M79)	✗
Renal tubulo-interstitial diseases (N10-N16)	✓
Urolithiasis (N20-N23)	✓
Diseases of male genital organs (N40-N51)	✗
Noninflammatory disorders of female genital tract (N80-N98)	✗

245 Table S41: Inclusion and exclusion criteria for cluster 40. Female ratio: 34%, mean age of patients: 44, mortality: 0%.

Intestinal infectious diseases (A00-A09)	✗
Malignant neoplasms (C00-C97)	✗
Benign neoplasms (D10-D36)	✗
Mental and behavioural disorders due to psychoactive substance use (F10-F19)	✗
Neurotic, stress-related and somatoform disorders (F40-F48)	✓
Episodic and paroxysmal disorders (G40-G47)	✗
Disorders of lens (H25-H28)	✗
Hypertensive diseases (I10-I15)	✗
Other forms of heart disease (I30-I52)	✗
Diseases of veins, lymphatic vessels and lymph nodes, not elsewhere classified (I80-I89)	✗
Acute upper respiratory infections (J00-J06)	✗
Other diseases of upper respiratory tract (J30-J39)	✗
Diseases of oral cavity, salivary glands and jaws (K00-K14)	✗
Diseases of appendix (K35-K38)	✗
Hernia (K40-K46)	✗
Other diseases of intestines (K55-K63)	✗
Disorders of gallbladder, biliary tract and pancreas (K80-K87)	✗
Infections of the skin and subcutaneous tissue (L00-L08)	✗
Arthropathies (M00-M25)	✗
Dorsopathies (M40-M54)	✗
Soft tissue disorders (M60-M79)	✗
Diseases of male genital organs (N40-N51)	✗
Noninflammatory disorders of female genital tract (N80-N98)	✗

Table S42: Inclusion and exclusion criteria for cluster 41. Female ratio: 60%, mean age of patients: 34, mortality: 0%.

Intestinal infectious diseases (A00-A09)	✗
Malignant neoplasms (C00-C97)	✗
Benign neoplasms (D10-D36)	✗
Mental and behavioural disorders due to psychoactive substance use (F10-F19)	✗
Episodic and paroxysmal disorders (G40-G47)	✗
Disorders of lens (H25-H28)	✗
Hypertensive diseases (I10-I15)	✗
Other forms of heart disease (I30-I52)	✗
Diseases of veins, lymphatic vessels and lymph nodes, not elsewhere classified (I80-I89)	✗
Acute upper respiratory infections (J00-J06)	✗
Other diseases of upper respiratory tract (J30-J39)	✗
Diseases of oral cavity, salivary glands and jaws (K00-K14)	✗
Diseases of appendix (K35-K38)	✗
Hernia (K40-K46)	✗
Other diseases of intestines (K55-K63)	✗
Disorders of gallbladder, biliary tract and pancreas (K80-K87)	✓
Infections of the skin and subcutaneous tissue (L00-L08)	✗
Arthropathies (M00-M25)	✗
Dorsopathies (M40-M54)	✗
Soft tissue disorders (M60-M79)	✗
Diseases of male genital organs (N40-N51)	✗
Noninflammatory disorders of female genital tract (N80-N98)	✗

Table S43: Inclusion and exclusion criteria for cluster 42. Female ratio: 65%, mean age of patients: 49, mortality: 0%.

Intestinal infectious diseases (A00-A09)	✗
Malignant neoplasms (C00-C97)	✗
Benign neoplasms (D10-D36)	✗
Mental and behavioural disorders due to psychoactive substance use (F10-F19)	✗
Episodic and paroxysmal disorders (G40-G47)	✗
Disorders of lens (H25-H28)	✗
Hypertensive diseases (I10-I15)	✗
Other forms of heart disease (I30-I52)	✗
Diseases of veins, lymphatic vessels and lymph nodes, not elsewhere classified (I80-I89)	✗
Acute upper respiratory infections (J00-J06)	✗
Other diseases of upper respiratory tract (J30-J39)	✗
Diseases of oral cavity, salivary glands and jaws (K00-K14)	✗
Diseases of appendix (K35-K38)	✗
Hernia (K40-K46)	✗
Other diseases of intestines (K55-K63)	✗
Infections of the skin and subcutaneous tissue (L00-L08)	✓
Arthropathies (M00-M25)	✗
Dorsopathies (M40-M54)	✗
Soft tissue disorders (M60-M79)	✗
Diseases of male genital organs (N40-N51)	✗
Noninflammatory disorders of female genital tract (N80-N98)	✗

Table S44: Inclusion and exclusion criteria for cluster 43. Female ratio: 34%, mean age of patients: 31, mortality: 0%.

Intestinal infectious diseases (A00-A09)	✗
Malignant neoplasms (C00-C97)	✗
Benign neoplasms (D10-D36)	✗
Mental and behavioural disorders due to psychoactive substance use (F10-F19)	✗
Episodic and paroxysmal disorders (G40-G47)	✗
Disorders of lens (H25-H28)	✓
Disorders of choroid and retina (H30-H36)	✗
Hypertensive diseases (I10-I15)	✗
Other forms of heart disease (I30-I52)	✗
Diseases of veins, lymphatic vessels and lymph nodes, not elsewhere classified (I80-I89)	✗
Acute upper respiratory infections (J00-J06)	✗
Other diseases of upper respiratory tract (J30-J39)	✗
Diseases of oral cavity, salivary glands and jaws (K00-K14)	✗
Diseases of appendix (K35-K38)	✗
Hernia (K40-K46)	✗
Other diseases of intestines (K55-K63)	✗
Arthropathies (M00-M25)	✗
Dorsopathies (M40-M54)	✗
Soft tissue disorders (M60-M79)	✗
Diseases of male genital organs (N40-N51)	✗
Noninflammatory disorders of female genital tract (N80-N98)	✗

Table S45: Inclusion and exclusion criteria for cluster 44. Female ratio: 58%, mean age of patients: 71, mortality: 0%.

Intestinal infectious diseases (A00-A09)	✗
Malignant neoplasms (C00-C97)	✗
Benign neoplasms (D10-D36)	✗
Mental and behavioural disorders due to psychoactive substance use (F10-F19)	✗
Episodic and paroxysmal disorders (G40-G47)	✗
Disorders of lens (H25-H28)	✓
Disorders of choroid and retina (H30-H36)	✓
Hypertensive diseases (I10-I15)	✗
Other forms of heart disease (I30-I52)	✗
Diseases of veins, lymphatic vessels and lymph nodes, not elsewhere classified (I80-I89)	✗
Acute upper respiratory infections (J00-J06)	✗
Other diseases of upper respiratory tract (J30-J39)	✗
Diseases of oral cavity, salivary glands and jaws (K00-K14)	✗
Diseases of appendix (K35-K38)	✗
Hernia (K40-K46)	✗
Other diseases of intestines (K55-K63)	✗
Arthropathies (M00-M25)	✗
Dorsopathies (M40-M54)	✗
Soft tissue disorders (M60-M79)	✗
Diseases of male genital organs (N40-N51)	✗
Noninflammatory disorders of female genital tract (N80-N98)	✗

Table S46: Inclusion and exclusion criteria for cluster 45. Female ratio: 57%, mean age of patients: 71, mortality: 0%.

Intestinal infectious diseases (A00-A09)	✗
Malignant neoplasms (C00-C97)	✗
Benign neoplasms (D10-D36)	✗
Mental and behavioural disorders due to psychoactive substance use (F10-F19)	✗
Episodic and paroxysmal disorders (G40-G47)	✗
Hypertensive diseases (I10-I15)	✗
Other forms of heart disease (I30-I52)	✗
Diseases of veins, lymphatic vessels and lymph nodes, not elsewhere classified (I80-I89)	✗
Acute upper respiratory infections (J00-J06)	✗
Other diseases of upper respiratory tract (J30-J39)	✗
Diseases of oral cavity, salivary glands and jaws (K00-K14)	✓
Diseases of appendix (K35-K38)	✗
Hernia (K40-K46)	✗
Other diseases of intestines (K55-K63)	✗
Arthropathies (M00-M25)	✗
Dorsopathies (M40-M54)	✗
Soft tissue disorders (M60-M79)	✗
Diseases of male genital organs (N40-N51)	✗
Noninflammatory disorders of female genital tract (N80-N98)	✗

Table S47: Inclusion and exclusion criteria for cluster 46. Female ratio: 52%, mean age of patients: 25, mortality: 0%.

Intestinal infectious diseases (A00-A09)	✗
Malignant neoplasms (C00-C97)	✗
Benign neoplasms (D10-D36)	✗
Mental and behavioural disorders due to psychoactive substance use (F10-F19)	✗
Episodic and paroxysmal disorders (G40-G47)	✗
Hypertensive diseases (I10-I15)	✗
Ischaemic heart diseases (I20-I25)	✗
Other forms of heart disease (I30-I52)	✓
Diseases of veins, lymphatic vessels and lymph nodes, not elsewhere classified (I80-I89)	✗
Acute upper respiratory infections (J00-J06)	✗
Other diseases of upper respiratory tract (J30-J39)	✗
Diseases of appendix (K35-K38)	✗
Hernia (K40-K46)	✗
Other diseases of intestines (K55-K63)	✗
Arthropathies (M00-M25)	✗
Dorsopathies (M40-M54)	✗
Soft tissue disorders (M60-M79)	✗
Diseases of male genital organs (N40-N51)	✗
Noninflammatory disorders of female genital tract (N80-N98)	✗

Table S48: Inclusion and exclusion criteria for cluster 47. Female ratio: 51%, mean age of patients: 61, mortality: 3%.

Intestinal infectious diseases (A00-A09)	✗
Malignant neoplasms (C00-C97)	✗
Benign neoplasms (D10-D36)	✗
Mental and behavioural disorders due to psychoactive substance use (F10-F19)	✗
Episodic and paroxysmal disorders (G40-G47)	✗
Hypertensive diseases (I10-I15)	✗
Ischaemic heart diseases (I20-I25)	✓
Other forms of heart disease (I30-I52)	✓
Diseases of veins, lymphatic vessels and lymph nodes, not elsewhere classified (I80-I89)	✗
Acute upper respiratory infections (J00-J06)	✗
Other diseases of upper respiratory tract (J30-J39)	✗
Diseases of appendix (K35-K38)	✗
Hernia (K40-K46)	✗
Other diseases of intestines (K55-K63)	✗
Arthropathies (M00-M25)	✗
Dorsopathies (M40-M54)	✗
Soft tissue disorders (M60-M79)	✗
Diseases of male genital organs (N40-N51)	✗
Noninflammatory disorders of female genital tract (N80-N98)	✗

Table S49: Inclusion and exclusion criteria for cluster 48. Female ratio: 46%, mean age of patients: 76, mortality: 6%.

Intestinal infectious diseases (A00-A09)	✗
Malignant neoplasms (C00-C97)	✗
Benign neoplasms (D10-D36)	✗
Mental and behavioural disorders due to psychoactive substance use (F10-F19)	✗
Episodic and paroxysmal disorders (G40-G47)	✓
Hypertensive diseases (I10-I15)	✗
Diseases of veins, lymphatic vessels and lymph nodes, not elsewhere classified (I80-I89)	✗
Acute upper respiratory infections (J00-J06)	✗
Other diseases of upper respiratory tract (J30-J39)	✗
Diseases of appendix (K35-K38)	✗
Hernia (K40-K46)	✗
Other diseases of intestines (K55-K63)	✗
Arthropathies (M00-M25)	✗
Dorsopathies (M40-M54)	✗
Soft tissue disorders (M60-M79)	✗
Diseases of male genital organs (N40-N51)	✗
Noninflammatory disorders of female genital tract (N80-N98)	✗

Table S50: Inclusion and exclusion criteria for cluster 49. Female ratio: 46%, mean age of patients: 40, mortality: 0%.

Intestinal infectious diseases (A00-A09)	✗
Malignant neoplasms (C00-C97)	✗
Benign neoplasms (D10-D36)	✗
Mental and behavioural disorders due to psychoactive substance use (F10-F19)	✗
Hypertensive diseases (I10-I15)	✗
Diseases of veins, lymphatic vessels and lymph nodes, not elsewhere classified (I80-I89)	✗
Acute upper respiratory infections (J00-J06)	✗
Other diseases of upper respiratory tract (J30-J39)	✗
Diseases of appendix (K35-K38)	✗
Hernia (K40-K46)	✗
Other diseases of intestines (K55-K63)	✗
Arthropathies (M00-M25)	✗
Dorsopathies (M40-M54)	✗
Soft tissue disorders (M60-M79)	✓
Diseases of male genital organs (N40-N51)	✗
Noninflammatory disorders of female genital tract (N80-N98)	✗

Table S51: Inclusion and exclusion criteria for cluster 50. Female ratio: 45%, mean age of patients: 45, mortality: 0%.

Intestinal infectious diseases (A00-A09)	✗
Malignant neoplasms (C00-C97)	✗
Benign neoplasms (D10-D36)	✓
Mental and behavioural disorders due to psychoactive substance use (F10-F19)	✗
Hypertensive diseases (I10-I15)	✗
Diseases of veins, lymphatic vessels and lymph nodes, not elsewhere classified (I80-I89)	✗
Acute upper respiratory infections (J00-J06)	✗
Other diseases of upper respiratory tract (J30-J39)	✗
Diseases of appendix (K35-K38)	✗
Hernia (K40-K46)	✗
Other diseases of intestines (K55-K63)	✗
Arthropathies (M00-M25)	✗
Dorsopathies (M40-M54)	✗
Diseases of male genital organs (N40-N51)	✗
Noninflammatory disorders of female genital tract (N80-N98)	✗

Table S52: Inclusion and exclusion criteria for cluster 51. Female ratio: 67%, mean age of patients: 42, mortality: 0%.

Intestinal infectious diseases (A00-A09)	✗
Malignant neoplasms (C00-C97)	✗
Mental and behavioural disorders due to psychoactive substance use (F10-F19)	✗
Hypertensive diseases (I10-I15)	✗
Diseases of veins, lymphatic vessels and lymph nodes, not elsewhere classified (I80-I89)	✗
Acute upper respiratory infections (J00-J06)	✗
Other diseases of upper respiratory tract (J30-J39)	✗
Diseases of appendix (K35-K38)	✓
Hernia (K40-K46)	✗
Other diseases of intestines (K55-K63)	✗
Arthropathies (M00-M25)	✗
Dorsopathies (M40-M54)	✗
Diseases of male genital organs (N40-N51)	✗
Noninflammatory disorders of female genital tract (N80-N98)	✗

Table S53: Inclusion and exclusion criteria for cluster 52. Female ratio: 52%, mean age of patients: 26, mortality: 0%.

Intestinal infectious diseases (A00-A09)	✗
Malignant neoplasms (C00-C97)	✗
Mental and behavioural disorders due to psychoactive substance use (F10-F19)	✗
Hypertensive diseases (I10-I15)	✗
Diseases of veins, lymphatic vessels and lymph nodes, not elsewhere classified (I80-I89)	✗
Acute upper respiratory infections (J00-J06)	✓
Other acute lower respiratory infections (J20-J22)	✗
Other diseases of upper respiratory tract (J30-J39)	✗
Hernia (K40-K46)	✗
Other diseases of intestines (K55-K63)	✗
Arthropathies (M00-M25)	✗
Dorsopathies (M40-M54)	✗
Diseases of male genital organs (N40-N51)	✗
Noninflammatory disorders of female genital tract (N80-N98)	✗

Table S54: Inclusion and exclusion criteria for cluster 53. Female ratio: 50%, mean age of patients: 18, mortality: 0%.

Intestinal infectious diseases (A00-A09)	✗
Malignant neoplasms (C00-C97)	✗
Mental and behavioural disorders due to psychoactive substance use (F10-F19)	✗
Hypertensive diseases (I10-I15)	✗
Diseases of veins, lymphatic vessels and lymph nodes, not elsewhere classified (I80-I89)	✗
Acute upper respiratory infections (J00-J06)	✓
Other acute lower respiratory infections (J20-J22)	✓
Other diseases of upper respiratory tract (J30-J39)	✗
Hernia (K40-K46)	✗
Other diseases of intestines (K55-K63)	✗
Arthropathies (M00-M25)	✗
Dorsopathies (M40-M54)	✗
Diseases of male genital organs (N40-N51)	✗
Noninflammatory disorders of female genital tract (N80-N98)	✗

Table S55: Inclusion and exclusion criteria for cluster 54. Female ratio: 45%, mean age of patients: 10, mortality: 0%.

Intestinal infectious diseases (A00-A09)	✗
Malignant neoplasms (C00-C97)	✓
Neoplasms of uncertain or unknown behaviour (D37-D48)	✗
Mental and behavioural disorders due to psychoactive substance use (F10-F19)	✗
Hypertensive diseases (I10-I15)	✗
Diseases of veins, lymphatic vessels and lymph nodes, not elsewhere classified (I80-I89)	✗
Other diseases of upper respiratory tract (J30-J39)	✗
Hernia (K40-K46)	✗
Other diseases of intestines (K55-K63)	✗
Arthropathies (M00-M25)	✗
Dorsopathies (M40-M54)	✗
Diseases of male genital organs (N40-N51)	✗
Noninflammatory disorders of female genital tract (N80-N98)	✗

Table S56: Inclusion and exclusion criteria for cluster 55. Female ratio: 51%, mean age of patients: 61, mortality: 3%.

276

Intestinal infectious diseases (A00-A09)	✗
Malignant neoplasms (C00-C97)	✓
Neoplasms of uncertain or unknown behaviour (D37-D48)	✓
Mental and behavioural disorders due to psychoactive substance use (F10-F19)	✗
Hypertensive diseases (I10-I15)	✗
Diseases of veins, lymphatic vessels and lymph nodes, not elsewhere classified (I80-I89)	✗
Other diseases of upper respiratory tract (J30-J39)	✗
Hernia (K40-K46)	✗
Other diseases of intestines (K55-K63)	✗
Arthropathies (M00-M25)	✗
Dorsopathies (M40-M54)	✗
Diseases of male genital organs (N40-N51)	✗
Noninflammatory disorders of female genital tract (N80-N98)	✗

277 Table S57: Inclusion and exclusion criteria for cluster 56. Female ratio: 47%, mean age of patients: 58, mortality: 4%.

278

Intestinal infectious diseases (A00-A09)	✗
Mental and behavioural disorders due to psychoactive substance use (F10-F19)	✗
Hypertensive diseases (I10-I15)	✗
Diseases of veins, lymphatic vessels and lymph nodes, not elsewhere classified (I80-I89)	✗
Other diseases of upper respiratory tract (J30-J39)	✗
Diseases of oesophagus, stomach and duodenum (K20-K31)	✗
Hernia (K40-K46)	✓
Other diseases of intestines (K55-K63)	✗
Arthropathies (M00-M25)	✗
Dorsopathies (M40-M54)	✗
Diseases of male genital organs (N40-N51)	✗
Noninflammatory disorders of female genital tract (N80-N98)	✗

279 Table S58: Inclusion and exclusion criteria for cluster 57. Female ratio: 24%, mean age of patients: 43, mortality: 0%.

280

Intestinal infectious diseases (A00-A09)	✗
Mental and behavioural disorders due to psychoactive substance use (F10-F19)	✗
Hypertensive diseases (I10-I15)	✗
Diseases of veins, lymphatic vessels and lymph nodes, not elsewhere classified (I80-I89)	✗
Other diseases of upper respiratory tract (J30-J39)	✗
Diseases of oesophagus, stomach and duodenum (K20-K31)	✓
Hernia (K40-K46)	✓
Other diseases of intestines (K55-K63)	✗
Arthropathies (M00-M25)	✗
Dorsopathies (M40-M54)	✗
Diseases of male genital organs (N40-N51)	✗
Noninflammatory disorders of female genital tract (N80-N98)	✗

281 Table S59: Inclusion and exclusion criteria for cluster 58. Female ratio: 40%, mean age of patients: 51, mortality: 0%.

282

Intestinal infectious diseases (A00-A09)	✗
Mental and behavioural disorders due to psychoactive substance use (F10-F19)	✗
Hypertensive diseases (I10-I15)	✗
Diseases of veins, lymphatic vessels and lymph nodes, not elsewhere classified (I80-I89)	✗
Other diseases of upper respiratory tract (J30-J39)	✗
Other diseases of intestines (K55-K63)	✗
Arthropathies (M00-M25)	✗
Dorsopathies (M40-M54)	✗
Other diseases of urinary system (N30-N39)	✗
Diseases of male genital organs (N40-N51)	✓
Noninflammatory disorders of female genital tract (N80-N98)	✗

283 Table S60: Inclusion and exclusion criteria for cluster 59. Female ratio: 0%, mean age of patients: 30, mortality: 0%.

Intestinal infectious diseases (A00-A09)	✗
Mental and behavioural disorders due to psychoactive substance use (F10-F19)	✗
Hypertensive diseases (I10-I15)	✗
Diseases of veins, lymphatic vessels and lymph nodes, not elsewhere classified (I80-I89)	✗
Other diseases of upper respiratory tract (J30-J39)	✗
Other diseases of intestines (K55-K63)	✗
Arthropathies (M00-M25)	✗
Dorsopathies (M40-M54)	✗
Other diseases of urinary system (N30-N39)	✓
Diseases of male genital organs (N40-N51)	✓
Noninflammatory disorders of female genital tract (N80-N98)	✗

Table S61: Inclusion and exclusion criteria for cluster 60. Female ratio: 0%, mean age of patients: 54, mortality: 1%.

Intestinal infectious diseases (A00-A09)	✗
Mental and behavioural disorders due to psychoactive substance use (F10-F19)	✗
Hypertensive diseases (I10-I15)	✗
Diseases of veins, lymphatic vessels and lymph nodes, not elsewhere classified (I80-I89)	✓
Other diseases of upper respiratory tract (J30-J39)	✗
Diseases of oesophagus, stomach and duodenum (K20-K31)	✗
Other diseases of intestines (K55-K63)	✗
Arthropathies (M00-M25)	✗
Dorsopathies (M40-M54)	✗
Noninflammatory disorders of female genital tract (N80-N98)	✗

Table S62: Inclusion and exclusion criteria for cluster 61. Female ratio: 55%, mean age of patients: 48, mortality: 0%.

Intestinal infectious diseases (A00-A09)	✗
Mental and behavioural disorders due to psychoactive substance use (F10-F19)	✗
Hypertensive diseases (I10-I15)	✗
Diseases of veins, lymphatic vessels and lymph nodes, not elsewhere classified (I80-I89)	✓
Other diseases of upper respiratory tract (J30-J39)	✗
Diseases of oesophagus, stomach and duodenum (K20-K31)	✓
Other diseases of intestines (K55-K63)	✗
Arthropathies (M00-M25)	✗
Dorsopathies (M40-M54)	✗
Noninflammatory disorders of female genital tract (N80-N98)	✗

Table S63: Inclusion and exclusion criteria for cluster 62. Female ratio: 45%, mean age of patients: 57, mortality: 1%.

Intestinal infectious diseases (A00-A09)	✗
Metabolic disorders (E70-E90)	✗
Mental and behavioural disorders due to psychoactive substance use (F10-F19)	✗
Mood [affective] disorders (F30-F39)	✗
Hypertensive diseases (I10-I15)	✗
Other diseases of upper respiratory tract (J30-J39)	✗
Other diseases of intestines (K55-K63)	✗
Arthropathies (M00-M25)	✗
Dorsopathies (M40-M54)	✓
Noninflammatory disorders of female genital tract (N80-N98)	✗

Table S64: Inclusion and exclusion criteria for cluster 63. Female ratio: 48%, mean age of patients: 48, mortality: 0%.

Intestinal infectious diseases (A00-A09)	✗
Metabolic disorders (E70-E90)	✗
Mental and behavioural disorders due to psychoactive substance use (F10-F19)	✓
Mood [affective] disorders (F30-F39)	✗
Neurotic, stress-related and somatoform disorders (F40-F48)	✗
Hypertensive diseases (I10-I15)	✗
Other diseases of upper respiratory tract (J30-J39)	✗
Chronic lower respiratory diseases (J40-J47)	✗
Other diseases of intestines (K55-K63)	✗
Diseases of liver (K70-K77)	✗
Arthropathies (M00-M25)	✗
Noninflammatory disorders of female genital tract (N80-N98)	✗

Table S65: Inclusion and exclusion criteria for cluster 64. Female ratio: 30%, mean age of patients: 37, mortality: 0%.

Intestinal infectious diseases (A00-A09)	✗
Metabolic disorders (E70-E90)	✗
Mental and behavioural disorders due to psychoactive substance use (F10-F19)	✗
Mood [affective] disorders (F30-F39)	✓
Hypertensive diseases (I10-I15)	✗
Other diseases of upper respiratory tract (J30-J39)	✗
Other diseases of intestines (K55-K63)	✗
Arthropathies (M00-M25)	✗
Dorsopathies (M40-M54)	✓
Noninflammatory disorders of female genital tract (N80-N98)	✗

Table S66: Inclusion and exclusion criteria for cluster 65. Female ratio: 70%, mean age of patients: 52, mortality: 0%.

Intestinal infectious diseases (A00-A09)	✗
Metabolic disorders (E70-E90)	✓
Mental and behavioural disorders due to psychoactive substance use (F10-F19)	✗
Hypertensive diseases (I10-I15)	✗
Other diseases of upper respiratory tract (J30-J39)	✗
Other diseases of intestines (K55-K63)	✗
Arthropathies (M00-M25)	✗
Dorsopathies (M40-M54)	✓
Noninflammatory disorders of female genital tract (N80-N98)	✗

Table S67: Inclusion and exclusion criteria for cluster 66. Female ratio: 45%, mean age of patients: 57, mortality: 1%.

Intestinal infectious diseases (A00-A09)	✗
Metabolic disorders (E70-E90)	✓
Mental and behavioural disorders due to psychoactive substance use (F10-F19)	✓
Mood [affective] disorders (F30-F39)	✗
Neurotic, stress-related and somatoform disorders (F40-F48)	✗
Hypertensive diseases (I10-I15)	✗
Other diseases of upper respiratory tract (J30-J39)	✗
Chronic lower respiratory diseases (J40-J47)	✗
Other diseases of intestines (K55-K63)	✗
Diseases of liver (K70-K77)	✗
Arthropathies (M00-M25)	✗
Noninflammatory disorders of female genital tract (N80-N98)	✗

Table S68: Inclusion and exclusion criteria for cluster 67. Female ratio: 22%, mean age of patients: 50, mortality: 1%.

Intestinal infectious diseases (A00-A09)	✗
Mental and behavioural disorders due to psychoactive substance use (F10-F19)	✓
Mood [affective] disorders (F30-F39)	✗
Neurotic, stress-related and somatoform disorders (F40-F48)	✗
Hypertensive diseases (I10-I15)	✗
Other diseases of upper respiratory tract (J30-J39)	✗
Chronic lower respiratory diseases (J40-J47)	✓
Other diseases of intestines (K55-K63)	✗
Diseases of liver (K70-K77)	✗
Arthropathies (M00-M25)	✗
Noninflammatory disorders of female genital tract (N80-N98)	✗

Table S69: Inclusion and exclusion criteria for cluster 68. Female ratio: 28%, mean age of patients: 55, mortality: 2%.

Intestinal infectious diseases (A00-A09)	✗
Mental and behavioural disorders due to psychoactive substance use (F10-F19)	✓
Mood [affective] disorders (F30-F39)	✗
Neurotic, stress-related and somatoform disorders (F40-F48)	✓
Hypertensive diseases (I10-I15)	✗
Other diseases of upper respiratory tract (J30-J39)	✗
Other diseases of intestines (K55-K63)	✗
Diseases of liver (K70-K77)	✗
Arthropathies (M00-M25)	✗
Noninflammatory disorders of female genital tract (N80-N98)	✗

Table S70: Inclusion and exclusion criteria for cluster 69. Female ratio: 40%, mean age of patients: 36, mortality: 0%.

Intestinal infectious diseases (A00-A09)	✗
Mental and behavioural disorders due to psychoactive substance use (F10-F19)	✓
Mood [affective] disorders (F30-F39)	✗
Hypertensive diseases (I10-I15)	✗
Other diseases of upper respiratory tract (J30-J39)	✗
Other diseases of intestines (K55-K63)	✗
Diseases of liver (K70-K77)	✓
Arthropathies (M00-M25)	✗
Noninflammatory disorders of female genital tract (N80-N98)	✗

Table S71: Inclusion and exclusion criteria for cluster 70. Female ratio: 18%, mean age of patients: 52, mortality: 2%.

Intestinal infectious diseases (A00-A09)	✗
Mental and behavioural disorders due to psychoactive substance use (F10-F19)	✓
Mood [affective] disorders (F30-F39)	✓
Hypertensive diseases (I10-I15)	✗
Other diseases of upper respiratory tract (J30-J39)	✗
Other diseases of intestines (K55-K63)	✗
Arthropathies (M00-M25)	✗
Noninflammatory disorders of female genital tract (N80-N98)	✗

Table S72: Inclusion and exclusion criteria for cluster 71. Female ratio: 41%, mean age of patients: 43, mortality: 0%.

Intestinal infectious diseases (A00-A09)	✗
Malignant neoplasms (C00-C97)	✗
Hypertensive diseases (I10-I15)	✗
Diseases of veins, lymphatic vessels and lymph nodes, not elsewhere classified (I80-I89)	✗
Other diseases of upper respiratory tract (J30-J39)	✗
Diseases of oesophagus, stomach and duodenum (K20-K31)	✗
Hernia (K40-K46)	✗
Other diseases of intestines (K55-K63)	✓
Arthropathies (M00-M25)	✗
Noninflammatory disorders of female genital tract (N80-N98)	✗

Table S73: Inclusion and exclusion criteria for cluster 72. Female ratio: 47%, mean age of patients: 45, mortality: 0%.

Intestinal infectious diseases (A00-A09)	✗
Malignant neoplasms (C00-C97)	✗
Hypertensive diseases (I10-I15)	✗
Diseases of veins, lymphatic vessels and lymph nodes, not elsewhere classified (I80-I89)	✗
Other diseases of upper respiratory tract (J30-J39)	✗
Diseases of oesophagus, stomach and duodenum (K20-K31)	✗
Hernia (K40-K46)	✓
Other diseases of intestines (K55-K63)	✓
Arthropathies (M00-M25)	✗
Noninflammatory disorders of female genital tract (N80-N98)	✗

Table S74: Inclusion and exclusion criteria for cluster 73. Female ratio: 29%, mean age of patients: 58, mortality: 1%.

Intestinal infectious diseases (A00-A09)	✗
Malignant neoplasms (C00-C97)	✓
Hypertensive diseases (I10-I15)	✗
Diseases of veins, lymphatic vessels and lymph nodes, not elsewhere classified (I80-I89)	✗
Other diseases of upper respiratory tract (J30-J39)	✗
Diseases of oesophagus, stomach and duodenum (K20-K31)	✗
Other diseases of intestines (K55-K63)	✓
Arthropathies (M00-M25)	✗
Noninflammatory disorders of female genital tract (N80-N98)	✗

Table S75: Inclusion and exclusion criteria for cluster 74. Female ratio: 42%, mean age of patients: 68, mortality: 5%.

Intestinal infectious diseases (A00-A09)	✗
Hypertensive diseases (I10-I15)	✗
Diseases of veins, lymphatic vessels and lymph nodes, not elsewhere classified (I80-I89)	✓
Other diseases of upper respiratory tract (J30-J39)	✗
Diseases of oesophagus, stomach and duodenum (K20-K31)	✗
Other diseases of intestines (K55-K63)	✓
Arthropathies (M00-M25)	✗
Noninflammatory disorders of female genital tract (N80-N98)	✗

Table S76: Inclusion and exclusion criteria for cluster 75. Female ratio: 44%, mean age of patients: 55, mortality: 1%.

Intestinal infectious diseases (A00-A09)	✗
Hypertensive diseases (I10-I15)	✗
Other diseases of upper respiratory tract (J30-J39)	✗
Diseases of oesophagus, stomach and duodenum (K20-K31)	✓
Other diseases of intestines (K55-K63)	✓
Arthropathies (M00-M25)	✗
Noninflammatory disorders of female genital tract (N80-N98)	✗

Table S77: Inclusion and exclusion criteria for cluster 76. Female ratio: 42%, mean age of patients: 59, mortality: 1%.

Intestinal infectious diseases (A00-A09)	✓
Metabolic disorders (E70-E90)	✗
Hypertensive diseases (I10-I15)	✗
Acute upper respiratory infections (J00-J06)	✗
Other acute lower respiratory infections (J20-J22)	✗
Other diseases of upper respiratory tract (J30-J39)	✗
Arthropathies (M00-M25)	✗
Noninflammatory disorders of female genital tract (N80-N98)	✗

Table S78: Inclusion and exclusion criteria for cluster 77. Female ratio: 52%, mean age of patients: 21, mortality: 0%.

Intestinal infectious diseases (A00-A09)	✓
Metabolic disorders (E70-E90)	✗
Hypertensive diseases (I10-I15)	✗
Acute upper respiratory infections (J00-J06)	✗
Other acute lower respiratory infections (J20-J22)	✓
Other diseases of upper respiratory tract (J30-J39)	✗
Arthropathies (M00-M25)	✗
Noninflammatory disorders of female genital tract (N80-N98)	✗

Table S79: Inclusion and exclusion criteria for cluster 78. Female ratio: 42%, mean age of patients: 10, mortality: 0%.

Intestinal infectious diseases (A00-A09)	✓
Metabolic disorders (E70-E90)	✓
Hypertensive diseases (I10-I15)	✗
Acute upper respiratory infections (J00-J06)	✗
Other diseases of upper respiratory tract (J30-J39)	✗
Arthropathies (M00-M25)	✗
Noninflammatory disorders of female genital tract (N80-N98)	✗

Table S80: Inclusion and exclusion criteria for cluster 79. Female ratio: 51%, mean age of patients: 22, mortality: 1%.

Intestinal infectious diseases (A00-A09)	✓
Hypertensive diseases (I10-I15)	✗
Acute upper respiratory infections (J00-J06)	✓
Other diseases of upper respiratory tract (J30-J39)	✗
Arthropathies (M00-M25)	✗
Noninflammatory disorders of female genital tract (N80-N98)	✗

Table S81: Inclusion and exclusion criteria for cluster 80. Female ratio: 46%, mean age of patients: 9, mortality: 0%.

Benign neoplasms (D10-D36)	✗
Hypertensive diseases (I10-I15)	✗
Other diseases of upper respiratory tract (J30-J39)	✗
Arthropathies (M00-M25)	✗
Other diseases of urinary system (N30-N39)	✗
Inflammatory diseases of female pelvic organs (N70-N77)	✗
Noninflammatory disorders of female genital tract (N80-N98)	✓

Table S82: Inclusion and exclusion criteria for cluster 81. Female ratio: 100%, mean age of patients: 43, mortality: 0%.

Benign neoplasms (D10-D36)	✗
Hypertensive diseases (I10-I15)	✗
Other diseases of upper respiratory tract (J30-J39)	✗
Arthropathies (M00-M25)	✗
Other diseases of urinary system (N30-N39)	✓
Inflammatory diseases of female pelvic organs (N70-N77)	✗
Noninflammatory disorders of female genital tract (N80-N98)	✓

Table S83: Inclusion and exclusion criteria for cluster 82. Female ratio: 100%, mean age of patients: 51, mortality: 1%.

Benign neoplasms (D10-D36)	✗
Hypertensive diseases (I10-I15)	✗
Other diseases of upper respiratory tract (J30-J39)	✗
Arthropathies (M00-M25)	✗
Inflammatory diseases of female pelvic organs (N70-N77)	✓
Noninflammatory disorders of female genital tract (N80-N98)	✓

Table S84: Inclusion and exclusion criteria for cluster 83. Female ratio: 100%, mean age of patients: 39, mortality: 0%.

Benign neoplasms (D10-D36)	✓
Hypertensive diseases (I10-I15)	✗
Other diseases of upper respiratory tract (J30-J39)	✗
Arthropathies (M00-M25)	✗
Noninflammatory disorders of female genital tract (N80-N98)	✓

Table S85: Inclusion and exclusion criteria for cluster 84. Female ratio: 100%, mean age of patients: 48, mortality: 0%.

Metabolic disorders (E70-E90)	✗
Hypertensive diseases (I10-I15)	✗
Diseases of veins, lymphatic vessels and lymph nodes, not elsewhere classified (I80-I89)	✗
Other diseases of upper respiratory tract (J30-J39)	✗
Arthropathies (M00-M25)	✓
Dorsopathies (M40-M54)	✗
Soft tissue disorders (M60-M79)	✗
Osteopathies and chondropathies (M80-M94)	✗

Table S86: Inclusion and exclusion criteria for cluster 85. Female ratio: 47%, mean age of patients: 50, mortality: 0%.

Metabolic disorders (E70-E90)	✓
Hypertensive diseases (I10-I15)	✗
Diseases of veins, lymphatic vessels and lymph nodes, not elsewhere classified (I80-I89)	✗
Other diseases of upper respiratory tract (J30-J39)	✗
Arthropathies (M00-M25)	✓
Dorsopathies (M40-M54)	✗
Soft tissue disorders (M60-M79)	✗
Osteopathies and chondropathies (M80-M94)	✗

Table S87: Inclusion and exclusion criteria for cluster 86. Female ratio: 43%, mean age of patients: 61, mortality: 1%.

Hypertensive diseases (I10-I15)	✗
Diseases of veins, lymphatic vessels and lymph nodes, not elsewhere classified (I80-I89)	✓
Other diseases of upper respiratory tract (J30-J39)	✗
Arthropathies (M00-M25)	✓
Dorsopathies (M40-M54)	✗
Soft tissue disorders (M60-M79)	✗
Osteopathies and chondropathies (M80-M94)	✗

Table S88: Inclusion and exclusion criteria for cluster 87. Female ratio: 57%, mean age of patients: 59, mortality: 1%.

Hypertensive diseases (I10-I15)	✗
Other diseases of upper respiratory tract (J30-J39)	✗
Arthropathies (M00-M25)	✓
Dorsopathies (M40-M54)	✓
Soft tissue disorders (M60-M79)	✗
Osteopathies and chondropathies (M80-M94)	✗

Table S89: Inclusion and exclusion criteria for cluster 88. Female ratio: 54%, mean age of patients: 59, mortality: 0%.

Hypertensive diseases (I10-I15)	✗
Other diseases of upper respiratory tract (J30-J39)	✗
Arthropathies (M00-M25)	✓
Soft tissue disorders (M60-M79)	✓
Osteopathies and chondropathies (M80-M94)	✗

Table S90: Inclusion and exclusion criteria for cluster 89. Female ratio: 47%, mean age of patients: 52, mortality: 0%.

Hypertensive diseases (I10-I15)	✗
Other diseases of upper respiratory tract (J30-J39)	✗
Arthropathies (M00-M25)	✓
Soft tissue disorders (M60-M79)	✗
Osteopathies and chondropathies (M80-M94)	✓

Table S91: Inclusion and exclusion criteria for cluster 90. Female ratio: 55%, mean age of patients: 57, mortality: 1%.

Hypertensive diseases (I10-I15)	✗
Other diseases of upper respiratory tract (J30-J39)	✗
Arthropathies (M00-M25)	✓
Soft tissue disorders (M60-M79)	✓
Osteopathies and chondropathies (M80-M94)	✓

Table S92: Inclusion and exclusion criteria for cluster 91. Female ratio: 52%, mean age of patients: 56, mortality: 0%.

Intestinal infectious diseases (A00-A09)	✗
Benign neoplasms (D10-D36)	✗
Episodic and paroxysmal disorders (G40-G47)	✗
Diseases of middle ear and mastoid (H65-H75)	✗
Hypertensive diseases (I10-I15)	✗
Acute upper respiratory infections (J00-J06)	✗
Other diseases of upper respiratory tract (J30-J39)	✓
Chronic lower respiratory diseases (J40-J47)	✗
Diseases of oral cavity, salivary glands and jaws (K00-K14)	✗
Other disorders of the musculoskeletal system and connective tissue (M95-M99)	✗

Table S93: Inclusion and exclusion criteria for cluster 92. Female ratio: 46%, mean age of patients: 27, mortality: 0%.

Intestinal infectious diseases (A00-A09)	✗
Benign neoplasms (D10-D36)	✓
Episodic and paroxysmal disorders (G40-G47)	✗
Diseases of middle ear and mastoid (H65-H75)	✗
Hypertensive diseases (I10-I15)	✗
Acute upper respiratory infections (J00-J06)	✗
Other diseases of upper respiratory tract (J30-J39)	✓
Chronic lower respiratory diseases (J40-J47)	✗
Diseases of oral cavity, salivary glands and jaws (K00-K14)	✗
Other disorders of the musculoskeletal system and connective tissue (M95-M99)	✗

Table S94: Inclusion and exclusion criteria for cluster 93. Female ratio: 57%, mean age of patients: 40, mortality: 0%.

Intestinal infectious diseases (A00-A09)	✗
Episodic and paroxysmal disorders (G40-G47)	✗
Diseases of middle ear and mastoid (H65-H75)	✗
Hypertensive diseases (I10-I15)	✗
Acute upper respiratory infections (J00-J06)	✗
Other diseases of upper respiratory tract (J30-J39)	✓
Chronic lower respiratory diseases (J40-J47)	✗
Diseases of oral cavity, salivary glands and jaws (K00-K14)	✓
Other disorders of the musculoskeletal system and connective tissue (M95-M99)	✗

Table S95: Inclusion and exclusion criteria for cluster 94. Female ratio: 47%, mean age of patients: 28, mortality: 0%.

Intestinal infectious diseases (A00-A09)	✓
Episodic and paroxysmal disorders (G40-G47)	✗
Diseases of middle ear and mastoid (H65-H75)	✗
Hypertensive diseases (I10-I15)	✗
Acute upper respiratory infections (J00-J06)	✗
Other diseases of upper respiratory tract (J30-J39)	✓
Chronic lower respiratory diseases (J40-J47)	✗
Other disorders of the musculoskeletal system and connective tissue (M95-M99)	✗

Table S96: Inclusion and exclusion criteria for cluster 95. Female ratio: 48%, mean age of patients: 16, mortality: 0%.

Episodic and paroxysmal disorders (G40-G47)	✗
Diseases of middle ear and mastoid (H65-H75)	✗
Hypertensive diseases (I10-I15)	✗
Acute upper respiratory infections (J00-J06)	✗
Other diseases of upper respiratory tract (J30-J39)	✓
Chronic lower respiratory diseases (J40-J47)	✓
Other disorders of the musculoskeletal system and connective tissue (M95-M99)	✗

Table S97: Inclusion and exclusion criteria for cluster 96. Female ratio: 41%, mean age of patients: 34, mortality: 1%.

Episodic and paroxysmal disorders (G40-G47)	✗
Diseases of middle ear and mastoid (H65-H75)	✗
Hypertensive diseases (I10-I15)	✗
Acute upper respiratory infections (J00-J06)	✗
Other diseases of upper respiratory tract (J30-J39)	✓
Other disorders of the musculoskeletal system and connective tissue (M95-M99)	✓

Table S98: Inclusion and exclusion criteria for cluster 97. Female ratio: 43%, mean age of patients: 34, mortality: 0%.

Episodic and paroxysmal disorders (G40-G47)	✓
Diseases of middle ear and mastoid (H65-H75)	✗
Hypertensive diseases (I10-I15)	✗
Acute upper respiratory infections (J00-J06)	✗
Other diseases of upper respiratory tract (J30-J39)	✓

Table S99: Inclusion and exclusion criteria for cluster 98. Female ratio: 34%, mean age of patients: 38, mortality: 0%.

Diseases of middle ear and mastoid (H65-H75)	✓
Hypertensive diseases (I10-I15)	✗
Acute upper respiratory infections (J00-J06)	✗
Other diseases of upper respiratory tract (J30-J39)	✓

Table S100: Inclusion and exclusion criteria for cluster 99. Female ratio: 42%, mean age of patients: 10, mortality: 0%.

Diseases of middle ear and mastoid (H65-H75)	✗
Hypertensive diseases (I10-I15)	✗
Acute upper respiratory infections (J00-J06)	✓
Other diseases of upper respiratory tract (J30-J39)	✓

Table S101: Inclusion and exclusion criteria for cluster 100. Female ratio: 45%, mean age of patients: 21, mortality: 0%.

Diseases of middle ear and mastoid (H65-H75)	✓
Hypertensive diseases (I10-I15)	✗
Acute upper respiratory infections (J00-J06)	✓
Other diseases of upper respiratory tract (J30-J39)	✓

Table S102: Inclusion and exclusion criteria for cluster 101. Female ratio: 37%, mean age of patients: 9, mortality: 0%.

Malignant neoplasms (C00-C97)	✗
Diabetes mellitus (E10-E14)	✗
Obesity and other hyperalimentation (E65-E68)	✗
Metabolic disorders (E70-E90)	✗
Disorders of lens (H25-H28)	✗
Hypertensive diseases (I10-I15)	✓
Ischaemic heart diseases (I20-I25)	✗
Other forms of heart disease (I30-I52)	✗
Cerebrovascular diseases (I60-I69)	✗
Diseases of oesophagus, stomach and duodenum (K20-K31)	✗
Arthropathies (M00-M25)	✗
Dorsopathies (M40-M54)	✗

Table S103: Inclusion and exclusion criteria for cluster 102. Female ratio: 53%, mean age of patients: 61, mortality: 0%.

Malignant neoplasms (C00-C97)	✗
Diabetes mellitus (E10-E14)	✗
Obesity and other hyperalimentation (E65-E68)	✗
Metabolic disorders (E70-E90)	✗
Disorders of lens (H25-H28)	✓
Hypertensive diseases (I10-I15)	✓
Ischaemic heart diseases (I20-I25)	✗
Other forms of heart disease (I30-I52)	✗
Cerebrovascular diseases (I60-I69)	✗
Diseases of oesophagus, stomach and duodenum (K20-K31)	✗
Arthropathies (M00-M25)	✗
Dorsopathies (M40-M54)	✗

Table S104: Inclusion and exclusion criteria for cluster 103. Female ratio: 65%, mean age of patients: 77, mortality: 0%.

Malignant neoplasms (C00-C97)	✗
Diabetes mellitus (E10-E14)	✗
Obesity and other hyperalimentation (E65-E68)	✗
Metabolic disorders (E70-E90)	✗
Hypertensive diseases (I10-I15)	✓
Ischaemic heart diseases (I20-I25)	✗
Other forms of heart disease (I30-I52)	✗
Cerebrovascular diseases (I60-I69)	✗
Diseases of oesophagus, stomach and duodenum (K20-K31)	✓
Arthropathies (M00-M25)	✗
Dorsopathies (M40-M54)	✗

Table S105: Inclusion and exclusion criteria for cluster 104. Female ratio: 52%, mean age of patients: 64, mortality: 0%.

Malignant neoplasms (C00-C97)	✗
Diabetes mellitus (E10-E14)	✗
Obesity and other hyperalimentation (E65-E68)	✗
Metabolic disorders (E70-E90)	✗
Hypertensive diseases (I10-I15)	✓
Ischaemic heart diseases (I20-I25)	✓
Other forms of heart disease (I30-I52)	✗
Cerebrovascular diseases (I60-I69)	✗
Arthropathies (M00-M25)	✗
Dorsopathies (M40-M54)	✗

Table S106: Inclusion and exclusion criteria for cluster 105. Female ratio: 41%, mean age of patients: 69, mortality: 1%.

Malignant neoplasms (C00-C97)	✗
Diabetes mellitus (E10-E14)	✗
Obesity and other hyperalimentation (E65-E68)	✓
Metabolic disorders (E70-E90)	✗
Hypertensive diseases (I10-I15)	✓
Other forms of heart disease (I30-I52)	✗
Cerebrovascular diseases (I60-I69)	✗
Arthropathies (M00-M25)	✗
Dorsopathies (M40-M54)	✗

Table S107: Inclusion and exclusion criteria for cluster 106. Female ratio: 52%, mean age of patients: 54, mortality: 0%.

Malignant neoplasms (C00-C97)	✗
Diabetes mellitus (E10-E14)	✗
Metabolic disorders (E70-E90)	✗
Hypertensive diseases (I10-I15)	✓
Other forms of heart disease (I30-I52)	✗
Cerebrovascular diseases (I60-I69)	✓
Arthropathies (M00-M25)	✗
Dorsopathies (M40-M54)	✗

Table S108: Inclusion and exclusion criteria for cluster 107. Female ratio: 50%, mean age of patients: 73, mortality: 2%.

Malignant neoplasms (C00-C97)	✗
Diabetes mellitus (E10-E14)	✗
Metabolic disorders (E70-E90)	✗
Hypertensive diseases (I10-I15)	✓
Other forms of heart disease (I30-I52)	✗
Arthropathies (M00-M25)	✗
Dorsopathies (M40-M54)	✓

Table S109: Inclusion and exclusion criteria for cluster 108. Female ratio: 59%, mean age of patients: 64, mortality: 0%.

Malignant neoplasms (C00-C97)	✓
Diabetes mellitus (E10-E14)	✗
Metabolic disorders (E70-E90)	✗
Hypertensive diseases (I10-I15)	✓
Other forms of heart disease (I30-I52)	✗
Arthropathies (M00-M25)	✗

Table S110: Inclusion and exclusion criteria for cluster 109. Female ratio: 48%, mean age of patients: 71, mortality: 4%.

Diabetes mellitus (E10-E14)	✗
Metabolic disorders (E70-E90)	✗
Hypertensive diseases (I10-I15)	✓
Other forms of heart disease (I30-I52)	✗
Arthropathies (M00-M25)	✓
Dorsopathies (M40-M54)	✗

Table S111: Inclusion and exclusion criteria for cluster 110. Female ratio: 59%, mean age of patients: 68, mortality: 1%.

Diabetes mellitus (E10-E14)	✗
Metabolic disorders (E70-E90)	✗
Hypertensive diseases (I10-I15)	✓
Other forms of heart disease (I30-I52)	✗
Arthropathies (M00-M25)	✓
Dorsopathies (M40-M54)	✓

Table S112: Inclusion and exclusion criteria for cluster 111. Female ratio: 67%, mean age of patients: 70, mortality: 1%.

Diabetes mellitus (E10-E14)	✓
Obesity and other hyperalimentation (E65-E68)	✗
Metabolic disorders (E70-E90)	✗
Hypertensive diseases (I10-I15)	✓
Other forms of heart disease (I30-I52)	✗

Table S113: Inclusion and exclusion criteria for cluster 112. Female ratio: 49%, mean age of patients: 71, mortality: 2%.

Diabetes mellitus (E10-E14)	✗
Metabolic disorders (E70-E90)	✓
Hypertensive diseases (I10-I15)	✓
Ischaemic heart diseases (I20-I25)	✗
Other forms of heart disease (I30-I52)	✗
Cerebrovascular diseases (I60-I69)	✗
Diseases of oesophagus, stomach and duodenum (K20-K31)	✗
Dorsopathies (M40-M54)	✗

Table S114: Inclusion and exclusion criteria for cluster 113. Female ratio: 51%, mean age of patients: 65, mortality: 1%.

Diabetes mellitus (E10-E14)	✗
Metabolic disorders (E70-E90)	✗
Hypertensive diseases (I10-I15)	✓
Ischaemic heart diseases (I20-I25)	✗
Other forms of heart disease (I30-I52)	✓
Cerebrovascular diseases (I60-I69)	✗
Renal failure (N17-N19)	✗

Table S115: Inclusion and exclusion criteria for cluster 114. Female ratio: 55%, mean age of patients: 74, mortality: 2%.

Diabetes mellitus (E10-E14)	✗
Metabolic disorders (E70-E90)	✓
Hypertensive diseases (I10-I15)	✓
Ischaemic heart diseases (I20-I25)	✗
Other forms of heart disease (I30-I52)	✗
Cerebrovascular diseases (I60-I69)	✗
Diseases of oesophagus, stomach and duodenum (K20-K31)	✓
Dorsopathies (M40-M54)	✗

Table S116: Inclusion and exclusion criteria for cluster 115. Female ratio: 51%, mean age of patients: 65, mortality: 1%.

Diabetes mellitus (E10-E14)	✗
Metabolic disorders (E70-E90)	✓
Hypertensive diseases (I10-I15)	✓
Ischaemic heart diseases (I20-I25)	✗
Other forms of heart disease (I30-I52)	✗
Cerebrovascular diseases (I60-I69)	✗
Dorsopathies (M40-M54)	✓

Table S117: Inclusion and exclusion criteria for cluster 116. Female ratio: 59%, mean age of patients: 66, mortality: 1%.

Diabetes mellitus (E10-E14)	✗
Metabolic disorders (E70-E90)	✓
Hypertensive diseases (I10-I15)	✓
Ischaemic heart diseases (I20-I25)	✗
Other forms of heart disease (I30-I52)	✗
Cerebrovascular diseases (I60-I69)	✓

Table S118: Inclusion and exclusion criteria for cluster 117. Female ratio: 50%, mean age of patients: 72, mortality: 2%.

Diabetes mellitus (E10-E14)	✗
Metabolic disorders (E70-E90)	✓
Hypertensive diseases (I10-I15)	✓
Ischaemic heart diseases (I20-I25)	✓
Other forms of heart disease (I30-I52)	✗

Table S119: Inclusion and exclusion criteria for cluster 118. Female ratio: 36%, mean age of patients: 67, mortality: 1%.

Diabetes mellitus (E10-E14)	✓
Obesity and other hyperalimentation (E65-E68)	✓
Metabolic disorders (E70-E90)	✗
Hypertensive diseases (I10-I15)	✓
Other forms of heart disease (I30-I52)	✗

Table S120: Inclusion and exclusion criteria for cluster 119. Female ratio: 54%, mean age of patients: 63, mortality: 1%.

Diabetes mellitus (E10-E14)	✓
Metabolic disorders (E70-E90)	✓
Hypertensive diseases (I10-I15)	✓
Ischaemic heart diseases (I20-I25)	✗
Other forms of heart disease (I30-I52)	✗

Table S121: Inclusion and exclusion criteria for cluster 120. Female ratio: 50%, mean age of patients: 68, mortality: 1%.

Diabetes mellitus (E10-E14)	✓
Metabolic disorders (E70-E90)	✗
Hypertensive diseases (I10-I15)	✓
Ischaemic heart diseases (I20-I25)	✗
Other forms of heart disease (I30-I52)	✓
Cerebrovascular diseases (I60-I69)	✗
Renal failure (N17-N19)	✗

Table S122: Inclusion and exclusion criteria for cluster 121. Female ratio: 55%, mean age of patients: 77, mortality: 3%.

Metabolic disorders (E70-E90)	✓
Hypertensive diseases (I10-I15)	✓
Ischaemic heart diseases (I20-I25)	✗
Other forms of heart disease (I30-I52)	✓
Cerebrovascular diseases (I60-I69)	✗
Renal failure (N17-N19)	✗

Table S123: Inclusion and exclusion criteria for cluster 122. Female ratio: 55%, mean age of patients: 73, mortality: 2%.

Hypertensive diseases (I10-I15)	✓
Ischaemic heart diseases (I20-I25)	✗
Other forms of heart disease (I30-I52)	✓
Cerebrovascular diseases (I60-I69)	✓
Renal failure (N17-N19)	✗

Table S124: Inclusion and exclusion criteria for cluster 123. Female ratio: 59%, mean age of patients: 80, mortality: 5%.

Diabetes mellitus (E10-E14)	✗
Metabolic disorders (E70-E90)	✗
Hypertensive diseases (I10-I15)	✓
Ischaemic heart diseases (I20-I25)	✓
Other forms of heart disease (I30-I52)	✓
Cerebrovascular diseases (I60-I69)	✗
Renal failure (N17-N19)	✗

Table S125: Inclusion and exclusion criteria for cluster 124. Female ratio: 50%, mean age of patients: 77, mortality: 3%.

Diabetes mellitus (E10-E14)	✗
Metabolic disorders (E70-E90)	✓
Hypertensive diseases (I10-I15)	✓
Ischaemic heart diseases (I20-I25)	✓
Other forms of heart disease (I30-I52)	✓
Cerebrovascular diseases (I60-I69)	✗
Renal failure (N17-N19)	✗

Table S126: Inclusion and exclusion criteria for cluster 125. Female ratio: 43%, mean age of patients: 73, mortality: 2%.

Diabetes mellitus (E10-E14)	✓
Metabolic disorders (E70-E90)	✓
Hypertensive diseases (I10-I15)	✓
Ischaemic heart diseases (I20-I25)	✓
Other forms of heart disease (I30-I52)	✗

Table S127: Inclusion and exclusion criteria for cluster 126. Female ratio: 36%, mean age of patients: 69, mortality: 2%.

Diabetes mellitus (E10-E14)	✓
Hypertensive diseases (I10-I15)	✓
Ischaemic heart diseases (I20-I25)	✓
Other forms of heart disease (I30-I52)	✓
Cerebrovascular diseases (I60-I69)	✗
Renal failure (N17-N19)	✗

Table S128: Inclusion and exclusion criteria for cluster 127. Female ratio: 45%, mean age of patients: 75, mortality: 4%.

Hypertensive diseases (I10-I15)	✓
Ischaemic heart diseases (I20-I25)	✓
Other forms of heart disease (I30-I52)	✓
Cerebrovascular diseases (I60-I69)	✓
Renal failure (N17-N19)	✗

Table S129: Inclusion and exclusion criteria for cluster 128. Female ratio: 52%, mean age of patients: 80, mortality: 6%.

Organic, including symptomatic, mental disorders (F00-F09)	✗
Hypertensive diseases (I10-I15)	✓
Other forms of heart disease (I30-I52)	✓
Diseases of arteries, arterioles and capillaries (I70-I79)	✗
Renal failure (N17-N19)	✓

Table S130: Inclusion and exclusion criteria for cluster 129. Female ratio: 52%, mean age of patients: 80, mortality: 8%.

Organic, including symptomatic, mental disorders (F00-F09)	✓
Hypertensive diseases (I10-I15)	✓
Other forms of heart disease (I30-I52)	✓
Diseases of arteries, arterioles and capillaries (I70-I79)	✗
Renal failure (N17-N19)	✓

Table S131: Inclusion and exclusion criteria for cluster 130. Female ratio: 65%, mean age of patients: 86, mortality: 10%.

Hypertensive diseases (I10-I15)	✓
Other forms of heart disease (I30-I52)	✓
Diseases of arteries, arterioles and capillaries (I70-I79)	✓
Renal failure (N17-N19)	✓

Table S132: Inclusion and exclusion criteria for cluster 131. Female ratio: 49%, mean age of patients: 81, mortality: 11%.

			Absolute risk/%		
			50–59	60–69	70–79
Cluster ID	112	m	1.6 ± 0.2	2.5 ± 0.2	4.3 ± 0.2
		f	1.0 ± 0.2	1.7 ± 0.2	3.7 ± 0.2
	119	m	1.8 ± 0.3	3.2 ± 0.4	5.3 ± 0.7
		f	1.2 ± 0.3	2.6 ± 0.4	4.6 ± 0.5
	120	m	1.7 ± 0.2	2.6 ± 0.2	4.5 ± 0.3
		f	1.2 ± 0.2	2.2 ± 0.2	3.9 ± 0.3

Table S133: Absolute risk of stepping into the high cardiovascular mortality region within one year for patients in clusters 112, 119 and 120.

			Relative risk (all patients)			Relative risk (cluster 0)		
			50–59	60–69	70–79	50–59	60–69	70–79
Cluster ID	112	m	1.96 ± 0.11	1.57 ± 0.06	1.38 ± 0.05	1.58 ± 0.11	1.34 ± 0.06	1.20 ± 0.05
		f	2.60 ± 0.18	1.71 ± 0.10	1.49 ± 0.05	2.05 ± 0.18	1.46 ± 0.10	1.39 ± 0.06
	119	m	2.24 ± 0.19	1.99 ± 0.12	1.73 ± 0.12	1.81 ± 0.19	1.70 ± 0.12	1.50 ± 0.12
		f	2.93 ± 0.24	2.58 ± 0.14	1.86 ± 0.11	2.30 ± 0.24	2.21 ± 0.14	1.73 ± 0.11
	120	m	2.09 ± 0.12	1.58 ± 0.08	1.47 ± 0.07	1.68 ± 0.12	1.35 ± 0.08	1.28 ± 0.07
		f	3.04 ± 0.18	2.17 ± 0.10	1.59 ± 0.07	2.39 ± 0.18	1.86 ± 0.10	1.48 ± 0.07

Table S134: Relative risks for patients in clusters 112, 119 and 120 to step into the high cardiovascular mortality region within one year, compared with all patients of the same sex and age group who have not been assigned to the high cardiovascular mortality region (left) and patients of the same sex and age group in cluster 0 where patients have not been assigned any hospital diagnoses yet (right).

	$\|\mathbf{w} \circ \hat{\mathbf{p}}\|_1 / \|\mathbf{w} \circ \mathbf{p}\|_1$	$\|\Delta\mathbf{p}\|_1$	$\|\Delta\mathbf{p}\|_2$	f
Benchmark 1	84%	0.29	0.042	0.55
Benchmark 2	90%	0.18	0.033	0.48
DIVCLUS-T	96%	0.11	0.017	0.48

Table S135: Comparison of the performance of our approach to modeling patient trajectories based on clustering patients using DIVCLUS-T and two other benchmark algorithms. The meaning of the different quantities is explained in section S5.

Age gr.	Source	Target	Rate female>	Baseline<	Rate male<	Inclusion diag. source	New diag.
0	23	38	0.0196	0.0065	0.0097	N10–N16	N30–N39
1	4	41	0.0104	0.0044	0.0094	F90–F98	F40–F48
1	12	92	0.0109	0.0087	0.0109	B25–B34	J30–J39
1	52	63	0.0020	0.0016	0.0013	K35–K38	M40–M54
1	52	92	0.0088	0.0087	0.0061	K35–K38	J30–J39
1	53	100	0.0101	0.0087	0.0081	J00–J06	J30–J39
1	61	77	0.0043	0.0042	0.0042	I80–I89	A00–A09
1	64	69	0.0077	0.0044	0.0066	F10–F19	F40–F48
1	72	76	0.0030	0.0023	0.0027	K55–K63	K20–K31
2	35	52	0.0034	0.0027	0.0031	K20–K31	K35–K38
2	63	77	0.0026	0.0022	0.0019	M40–M54	A00–A09
2	77	80	0.0021	0.0017	0.0020	A00–A09	J00–J06
2	92	93	0.0028	0.0028	0.0020	J30–J39	D10–D36
2	92	94	0.0023	0.0019	0.0021	J30–J39	K00–K14
2	92	95	0.0022	0.0022	0.0021	J30–J39	A00–A09
2	102	106	0.0038	0.0015	0.0037	I10–I15	E65–E68
3	17	27	0.0055	0.0027	0.0052	F20–F29	F30–F39
3	27	41	0.0080	0.0028	0.0076	F30–F39	F40–F48
3	35	42	0.0028	0.0025	0.0026	K20–K31	K80–K87
3	63	65	0.0038	0.0027	0.0025	M40–M54	F30–F39
3	69	71	0.0159	0.0027	0.0147	F10–F19, F40–F48	F30–F39
3	72	75	0.0044	0.0041	0.0043	K55–K63	I80–I89

Age gr.	Source	Target	Rate female>	Baseline<	Rate male<	Inclusion diag. source	New diag.
3	92	93	0.0047	0.0046	0.0025	J30–J39	D10–D36
3	92	95	0.0014	0.0014	0.0013	J30–J39	A00–A09
4	27	41	0.0080	0.0038	0.0067	F30–F39	F40–F48
4	34	50	0.0092	0.0053	0.0077	G50–G59	M60–M79
4	50	63	0.0078	0.0077	0.0067	M60–M79	M40–M54
4	64	69	0.0051	0.0038	0.0035	F10–F19	F40–F48
4	64	71	0.0107	0.0049	0.0070	F10–F19	F30–F39
4	69	71	0.0226	0.0049	0.0205	F10–F19, F40–F48	F30–F39
4	70	71	0.0166	0.0049	0.0141	F10–F19, K70–K77	F30–F39
4	85	88	0.0081	0.0077	0.0076	M00–M25	M40–M54
4	85	89	0.0100	0.0053	0.0096	M00–M25	M60–M79
4	92	96	0.0022	0.0017	0.0021	J30–J39	J40–J47
4	102	108	0.0084	0.0077	0.0061	I10–I15	M40–M54
4	108	111	0.0145	0.0070	0.0144	I10–I15, M40–M54	M00–M25
4	110	111	0.0152	0.0077	0.0139	I10–I15, M00–M25	M40–M54
4	112	119	0.0144	0.0030	0.0100	E10–E14, I10–I15	E65–E68
5	34	50	0.0130	0.0094	0.0108	G50–G59	M60–M79
5	85	89	0.0127	0.0094	0.0126	M00–M25	M60–M79
5	85	90	0.0046	0.0046	0.0042	M00–M25	M80–M94
5	86	90	0.0077	0.0046	0.0057	E70–E90, M00–M25	M80–M94
5	88	89	0.0235	0.0094	0.0217	M00–M25, M40–M54	M60–M79
5	88	90	0.0087	0.0046	0.0061	M00–M25, M40–M54	M80–M94
5	89	91	0.0101	0.0046	0.0087	M00–M25, M60–M79	M80–M94
5	90	91	0.0205	0.0094	0.0175	M00–M25, M80–M94	M60–M79
5	108	111	0.0225	0.0149	0.0215	I10–I15, M40–M54	M00–M25
5	110	111	0.0164	0.0113	0.0157	I10–I15, M00–M25	M40–M54
5	112	119	0.0119	0.0055	0.0101	E10–E14, I10–I15	E65–E68
5	113	116	0.0141	0.0113	0.0133	E70–E90, I10–I15	M40–M54
6	88	90	0.0101	0.0080	0.0060	M00–M25, M40–M54	M80–M94
6	89	91	0.0111	0.0080	0.0066	M00–M25, M60–M79	M80–M94
6	108	111	0.0241	0.0190	0.0213	I10–I15, M40–M54	M00–M25
6	110	111	0.0156	0.0123	0.0135	I10–I15, M00–M25	M40–M54
6	111	116	0.0196	0.0173	0.0192	I10–I15, M00–M25, M40–M54	E70–E90
6	112	119	0.0084	0.0070	0.0075	E10–E14, I10–I15	E65–E68
6	113	116	0.0128	0.0123	0.0114	E70–E90, I10–I15	M40–M54
6	115	116	0.0177	0.0123	0.0163	E70–E90, I10–I15, K20–K31	M40–M54
7	15	45	0.0508	0.0248	0.0493	H30–H36	H25–H28
7	50	89	0.0218	0.0210	0.0210	M60–M79	M00–M25
7	88	90	0.0156	0.0140	0.0093	M00–M25, M40–M54	M80–M94
7	108	111	0.0295	0.0210	0.0270	I10–I15, M40–M54	M00–M25
7	110	111	0.0214	0.0160	0.0161	I10–I15, M00–M25	M40–M54
7	113	116	0.0177	0.0160	0.0120	E70–E90, I10–I15	M40–M54
7	115	116	0.0221	0.0160	0.0165	E70–E90, I10–I15, K20–K31	M40–M54
7	129	130	0.0125	0.0072	0.0123	I10–I15, I30–I52, N17–N19	F00–F09
8	88	90	0.0218	0.0199	0.0096	M00–M25, M40–M54	M80–M94
8	110	111	0.0183	0.0165	0.0165	I10–I15, M00–M25	M40–M54
8	124	128	0.0225	0.0220	0.0204	I10–I15, I20–I25, I30–I52	I60–I69
8	127	128	0.0249	0.0220	0.0231	E10–E14, I10–I15, I20–I25, I30–I52	I60–I69

Table S136: Cluster transitions which are significantly overrepresented in females compared with males. The columns ‘Source’ and ‘Target’ give the labels of the source and target cluster of the corresponding transition, ‘Rate female>’ gives the lower bound of the 95% confidence interval for the rate at which females of the corresponding age group step from the source cluster to the target cluster; ‘Rate male<’ gives the upper bound of the 95% confidence interval of the same rate for males. The column ‘Inclusion diag. source’ gives the inclusion criteria of the source cluster, ‘New diag.’ denotes the diagnoses which patients acquire when stepping from the source to the target cluster. The column ‘Baseline<’ gives the upper boundary of the 95% confidence interval of the incidence of the diagnosis block in the column ‘New diag.’ for women of the corresponding age group. To exclude trivial results, only transitions made by at least 20 male patients are listed.

Age gr.	Source	Target	Rate male>	Baseline<	Rate female<	Inclusion diag. source	New diag.
0	54	101	0.0171	0.0154	0.0156	J00–J06, J20–J22	J30–J39, H65–H75
0	78	99	0.0172	0.0154	0.0164	A00–A09, J20–J22	J30–J39, H65–H75
0	80	101	0.0228	0.0154	0.0185	A00–A09, J00–J06	J30–J39, H65–H75
2	0	52	0.0032	0.0031	0.0024	None	K35–K38
2	17	64	0.0149	0.0072	0.0091	F20–F29	F10–F19
2	23	40	0.0060	0.0016	0.0023	N10–N16	N20–N23
2	27	71	0.0125	0.0072	0.0091	F30–F39	F10–F19
2	50	89	0.0066	0.0053	0.0057	M60–M79	M00–M25
2	64	70	0.0010	0.0010	0.0010	F10–F19	K70–K77
2	64	71	0.0050	0.0023	0.0049	F10–F19	F30–F39
2	85	90	0.0031	0.0015	0.0025	M00–M25	M80–M94
3	23	40	0.0057	0.0026	0.0034	N10–N16	N20–N23

Age gr.	Source	Target	Rate male>	Baseline<	Rate female<	Inclusion diag. source	New diag.
3	27	71	0.0115	0.0067	0.0086	F30–F39	F10–F19
3	49	98	0.0087	0.0071	0.0042	G40–G47	J30–J39
3	64	70	0.0034	0.0024	0.0021	F10–F19	K70–K77
3	102	113	0.0056	0.0043	0.0055	I10–I15	E70–E90
4	27	71	0.0102	0.0085	0.0091	F30–F39	F10–F19
4	36	72	0.0070	0.0063	0.0068	K50–K52	K55–K63
4	49	98	0.0073	0.0052	0.0031	G40–G47	J30–J39
4	50	89	0.0126	0.0103	0.0126	M60–M79	M00–M25
4	64	70	0.0068	0.0043	0.0043	F10–F19	K70–K77
5	44	45	0.0092	0.0017	0.0073	H25–H28	H30–H36
5	49	98	0.0052	0.0040	0.0034	G40–G47	J30–J39
5	62	76	0.0153	0.0101	0.0126	I80–I89, K20–K31	K55–K63
5	67	118	0.0103	0.0101	0.0092	E70–E90, F10–F19	I10–I15, I20–I25
5	112	126	0.0107	0.0101	0.0089	E10–E14, I10–I15	E70–E90, I20–I25
5	114	129	0.0045	0.0022	0.0043	I10–I15, I30–I52	N17–N19
5	116	118	0.0102	0.0101	0.0085	E70–E90, I10–I15, M40–M54	I20–I25
5	117	118	0.0116	0.0101	0.0084	E70–E90, I10–I15, I60–I69	I20–I25
5	119	126	0.0103	0.0101	0.0101	E10–E14, E65–E68, I10–I15	E70–E90, I20–I25
5	120	126	0.0162	0.0101	0.0142	E10–E14, E70–E90, I10–I15	I20–I25
5	122	125	0.0145	0.0101	0.0141	E70–E90, I10–I15, I30–I52	I20–I25
6	20	56	0.0272	0.0167	0.0201	D37–D48	C00–C97
6	120	126	0.0167	0.0162	0.0158	E10–E14, E70–E90, I10–I15	I20–I25
6	127	131	0.0099	0.0054	0.0091	E10–E14, I10–I15, I20–I25, I30–I52	I70–I79, N17–N19
7	129	131	0.0232	0.0116	0.0232	I10–I15, I30–I52, N17–N19	I70–I79
8	103	109	0.0214	0.0213	0.0179	H25–H28, I10–I15	C00–C97

Table S137: Cluster transitions which are significantly overrepresented in males compared with females. The columns 'Source' and 'Target' give the labels of the source and target cluster of the corresponding transition, 'Rate male>' gives the lower bound of the 95% confidence interval for the rate at which males of the corresponding age group step from the source cluster to the target cluster; 'Rate female<' gives the upper bound of the 95% confidence interval of the same rate for females. The column 'Inclusion diag. source' gives the inclusion criteria of the source cluster, 'New diag.' denotes the diagnoses which patients acquire when stepping from the source to the target cluster. The column 'Baseline<' gives the upper boundary of the 95% confidence interval of the incidence of the diagnosis block in the column 'New diag.' for men of the corresponding age group. If two blocks are in the columns 'New diag', the smaller incidence value is given. To exclude trivial results, only transitions made by at least 20 female patients are listed.

References

[1] Marie Chavent, Yves Lechevallier, and Olivier Briant. DIVCLUS-T: A monothetic divisive hierarchical clustering method. *Computational Statistics and Data Analysis*, 52(2):687–701, 2007. ISSN 01679473. doi: 10.1016/j.csda.2007.03.013.

[2] Joe H Ward, Jr. Hierarchical grouping to optimize an objective function. *Journal of the American Statistical Association*, 58(301):236–244, 1963.

[3] David J. C. MacKay. *Information Theory, Inference, and Learning Algorithms.* Cambridge University Press, 2005.

[4] Christoper D. Manning, Prabhakar Raghavan, and Hinrich Schütze. *An Introduction to Information Retrieval.* Cambridge University Press, 2009.
